# Jiawei Buyang Huanwu Decoction modulates gut microbiota and metabolic profiles in a rat model of idiopathic pulmonary fibrosis

**DOI:** 10.3389/fmicb.2025.1704103

**Published:** 2025-12-02

**Authors:** Dandan Sun, Xinxin Cao, Jing Sun, Huimin Zhang, Yujun Liu

**Affiliations:** 1 National Engineering Laboratory for Tree Breeding, College of Biological Sciences and Biotechnology, Beijing Forestry University, Beijing, China; 2 Institute of Traditional Chinese Medicine Preparations, Shandong Academy of Chinese Medicine, Jinan, China; 3 Institute of Medicinal Plant Development, Peking Union Medical College and Chinese Academy of Medical Sciences, Beijing, China; 4 Department of Criminal Science and Technology, Liaoning Police College, Dalian, China; 5 Institute of Chinese Medicine Analysis, Shandong Academy of Chinese Medicine, Jinan, China

**Keywords:** idiopathic pulmonary fibrosis, Jiawei Buyang Huanwu Decoction, gut microbiota, metabolomics, gut–lung axis

## Abstract

**Introduction:**

Idiopathic pulmonary fibrosis (IPF) is a progressive lung disease with irreversible fibrosis and poor prognosis. Jiawei Buyang Huanwu Decoction (JBHD) has demonstrated therapeutic effects, but the exact mechanisms, particularly those mediated by the gut microbiota, remain largely unexplored. This study aimed to explore how JBHD modulates gut microbiota, and how these changes may influence host metabolic regulation in the context of IPF.

**Methods:**

The IPF model was established via intratracheal bleomycin injection. After 28 days of treatment, feces samples were obtained for 16S rRNA gene sequencing, whereas serum and urine samples were collected for metabolomic analyses.

**Results:**

Gut microbiota analysis showed that JBHD restored microbial dysbiosis in IPF rats. Differentially altered fecal microbes (DAFMs) reversed by JBHD included *Lactobacillus, Clostridium* sensu stricto 1, *Turicibacter*, and Christensenellaceae R-7 group at the genus level. The microbial functions reversed by JBHD in both KEGG Level 3 and COG analyses were related to amino acid metabolism, including Biosynthesis of amino acids (KEGG) and Amino acid transport and metabolism (COG). Serum and urine metabolomics showed that JBHD modified the metabolic profile of IPF rats. Among the differentially expressed metabolites (DEMs) altered by IPF, JBHD reversed 11 in serum and 13 in urine. Pathway analysis indicated that these DEMs were mainly associated with amino acid and lipid metabolism. The consistency between microbial functional predictions and host metabolomic findings in amino acid metabolism suggests that JBHD may influence host metabolic pathways through gut microbiota modulation. Functional prediction of the targets of reversed DEMs highlights signaling pathways related to immune regulation. Correlation and network analyses between DAFMs and DEMs reveal potential associations, implying that gut microbiota alterations may contribute to coordinated changes in host metabolism.

**Discussion:**

JBHD may act by reshaping specific microbial communities, which in turn could help restore related metabolic disturbances. These findings suggest a possible microbiota-mediated mechanism through which JBHD may exert its effects along the gut–lung axis.

## 1 Introduction

Idiopathic pulmonary fibrosis (IPF) is a progressive interstitial lung disease of unknown cause with poor prognosis (Raghu et al., 2022; Richeldi et al., 2025). It is driven by repeated alveolar epithelial injury, aberrant epithelial-fibroblast crosstalk, immune dysregulation, genetic and epigenetic susceptibility, fibroblast activation, myofibroblast differentiation, excessive extracellular matrix deposition, stem cell exhaustion, and impaired autophagy, ultimately leading to progressive lung fibrosis and irreversible loss of function (Mei et al., 2022). In addition to these mechanisms, growing evidence suggests that extrapulmonary systemic factors, especially those related to the gut microbiota, may also contribute to the progression of IPF by modulating immune homeostasis (Gurczynski et al., 2024).

Recent findings have revealed that gut microbiota and host metabolic alterations can profoundly influence pulmonary immune responses, suggesting a mechanistic link between microbiota-derived metabolites and IPF pathogenesis (Dong et al., 2024). In traditional Chinese medicine, the lung and large intestine are viewed as functionally connected—a concept increasingly supported by emerging evidence on the gut–lung axis, a notion further supported by clinical evidence showing associations between pulmonary and intestinal disease. In a cohort of 189 IPF patients, 23.3% were diagnosed with intestinal diseases, underscoring the role of the gut–lung axis (Stefania et al., 2023). Gut microbiota can influence IPF progression by enabling immune cells and cytokines to migrate from the intestine to the lungs through the circulation, and through microbiota-derived short-chain fatty acids like propionate, butyrate, and acetate, which bind to receptors on lung immune cells and shape pulmonary immune responses (Song et al., 2024). Emerging evidence has shown that gut microbiota can influence the progression of pulmonary fibrosis by modulating host metabolic pathways through their metabolites (Li et al., 2024). These observations highlight the importance of further investigating how gut microbiota-driven host metabolism influences pulmonary fibrosis progression.

Untargeted metabolomics is a powerful, unbiased approach for comprehensively profiling low-molecular-weight metabolites in biological systems, providing critical insights into drug mechanisms, biomarker discovery, and metabolic pathways (Gertsman and Barshop, 2018). By offering a holistic, system-level view of metabolic changes, it aligns well with the multi-component and multi-target nature of traditional Chinese medicine, making it an essential tool for elucidating its therapeutic mechanisms (Liao et al., 2025). Combined serum and urine untargeted metabolomics provides complementary insights into systemic metabolic alterations, as serum captures circulating metabolic profiles reflective of real-time physiological states, while urine represents filtered metabolic excretion, together offering a more comprehensive understanding of disease-related metabolic dysregulation (Du et al., 2023). Previous studies have shown that combined serum and urine untargeted metabolomics can effectively reveal metabolic dysregulation and potential biomarkers in various diseases, including autoimmune disorders (Du et al., 2023) and cancers (Chen et al., 2024), providing a solid foundation for its application in other complex conditions. Recent research has shown that combining gut microbiota data with serum and urine metabolite profiles provides meaningful insights—for example, causal links have been identified between gut microbes and blood metabolites (Liu et al., 2022), and valid associations have been found between microbial metabolism and urinary metabolites (Zheng et al., 2023). These results suggest that gut microbiota is not only closely connected to fecal metabolites but also significantly related to serum and urine metabolites, which are easier to collect and standardize in clinical practice. However, despite these advances, use of combined serum and urine untargeted metabolomics to investigate the multilevel therapeutic mechanisms of traditional Chinese medicine in IPF remains limited and requires further exploration.

Buyang Huanwu Decoction (BHD), documented in the Qing dynasty classic *Yi Lin Gai Cuo* by Wang Qingren, is a traditional Chinese medicine formula used to treat *qi* deficiency and blood stasis syndromes. It is composed of Astragali Radix (Huangqi), Paeoniae Radix Rubra (Chishao), Chuanxiong Rhizoma (Chuanxiong), Carthami Flos (Honghua), Pheretima (Dilong), Angelicae Sinensis Radix (Danggui), and Persicae Semen (Taoren). BHD has shown broad pharmacological activities, including treatment of chronic cerebral ischemia (Cao et al., 2024), attenuation of stroke-induced immunosuppression (Fu et al., 2024), anti-atherosclerotic effects (Fu et al., 2022), renal protection in diabetic nephropathy (Wu et al., 2021), and neuroprotection in intracerebral hemorrhage (Cai et al., 2023b). Importantly, recent studies have also demonstrated its therapeutic potential in IPF (Guo et al., 2024; Xing et al., 2024; Zhou et al., 2024), supporting its multi-target value in complex diseases. The Jiawei Buyang Huanwu Decoction (JBHD), formulated by adding Epimedii Folium (Yinyanghuo) and Curculiginis Rhizoma (Xianmao) to the original prescription, enhances its yang-tonifying effect and strengthens the combination of tonification and activation, thereby amplifying its efficacy in promoting blood circulation and dredging meridians. Both clinical studies and animal experiments have demonstrated that this modified formula exhibits greater therapeutic advantages over BHD in the treatment of IPF, showing promising therapeutic effects (Jiang, 2022). However, the precise mechanisms underlying JBHD’s therapeutic effects against IPF, particularly its influence on the gut microbiota and host metabolic alterations, remain largely unclear.

Taken together, this study aimed to elucidate the mechanisms through which JBHD acts on IPF. To this end, we integrated 16S rDNA gene sequencing with untargeted serum and urine metabolomics to probe gut microbiota–host metabolic crosstalk and its possible role in modulating the gut–lung axis in IPF rats. These efforts are expected to yield fresh insights into the multi-target therapeutic potential of JBHD against IPF, laying a solid groundwork for its clinical application and future investigations.

## 2 Materials and methods

### 2.1 Materials and chemicals

Astragali Radix (Huangqi, Lot No. 220201) prepared from dried roots of Astragalus membranaceus (Fisch.) Bge. var. Mongholicus (Bge.) Hsiao, Epimedii Folium (Yinyanghuo, Lot No. 200901) from dried leaves of Epimedium brevicornu Maxim., Paeoniae Radix Rubra (Chishao, Lot No. 220101) from dried roots of Paeonia lactiflora Pall., Chuanxiong Rhizoma (Chuanxiong, Lot No. 201002) from dried rhizome of Ligusticum chuanxiong Hort., Carthami Flos (Honghua, Lot No. 220302) from dried flowers of Carthamus tinctorius L., Pheretima (Dilong, Lot No. 210701) from dried body of Pheretima aspergillum (E. Perrier), and Curculiginis Rhizoma (Xianmao, Lot No. 201101) from dried rhizome of Curculigo orchioides Gaertn. were purchased from BaiWeiTang Chinese Herbal Medicine Drinks Slice Co., Ltd. (Jinan, Shandong, China). Persicae Semen (Taoren, Lot No. 2203200611) prepared from dried mature seeds of Prunus persica (L.) Batsch, and Angelicae Sinensis Radix (Dangguiwei, Lot No. 2203040032) from dried tail part of the roots of Angelica sinensis (Oliv.) Diels were purchased from Bozhou Huqiao Pharmaceutical Co., Ltd. (Bozhou, Anhui, China).

Bleomycin (Lot No. 2452851) was purchased from Thermo Fisher Scientific (Carlsbad, CA, United States). Pirfenidone capsules (PFD) (Lot No. 20220517) were purchased from Beijing Continent Pharmaceuticals Co., Ltd. (Beijing, China). Methanol, acetonitrile, and formic acid of chromatographic grade were bought from Thermo Fisher Scientific (Fairland, NJ, United States). Nine and twenty-one authentic standards were obtained from National Institutes for Food and Drug Control (Beijing, China) and Shanghai yuanye Bio-Technology Co., Ltd. (Shanghai, China), respectively (see details of the authentic standards in Supplementary File).

### 2.2 Preparation of JBHD and animal experiments

#### 2.2.1 Preparation of JBHD for animal administration

The nine herbs of the JBHD, as listed above, were mixed according to the prescribed ratio (Huangqi:Yinyanghuo:Xianmao: Chishao:Dangguiwei:Chuanxiong:Honghua:Taoren:Dilong = 30: 15:12:12:12:9:9:9:3). All herbs were soaked in purified water (1:10, w/v) for 30 min and then extracted in boiling water for 1 h. The filtrate was collected and the residue was re-extracted. The two filtrates were combined, then concentrated to obtain a solution containing decoction pieces at 1 g/mL for the low-dose JBHD group (JBHD-L). Using the same extraction procedure, another batch was concentrated to 2 g/mL for the high-dose JBHD group (JBHD-H). In addition, the chemical characterization of JBHD was conducted to identify representative compounds, and the sample preparation and LC–MS analytical parameters are described in Supplementary material.

#### 2.2.2 Animal treatment and sample collection

Specific-pathogen-free grade male Sprague–Dawley rats (180–200 g) were obtained from Jinan Pengyue Laboratory Animal Breeding Co. Ltd. (Jinan, China; License No. SCXK, 20190003). Rats were placed under a 12 h/12 h light/dark cycle and a constant environment (Temperature, 25 ± 2 °C; Relative humidity, 50–55%). Prior to the experiments, rats were allowed free access to food and water for 7 days to acclimate to the environment.

A total of thirty rats were randomly divided into five groups: Control, Model, PFD (dosed at 162 mg/kg), JBHD-L (dosed at 10 g/kg), and JBHD-H (dosed at 20 g/kg). The JBHD doses were based on previous studies (Yang, 2021; Jiang, 2022). In addition, rats in the Control and Model groups were given an equal volume of water orally. The IPF model was induced in the rats by a single intratracheal instillation of bleomycin (5 mg/kg). The Control group received intratracheal instillation of an equal amount of saline. The positive drug used in this study was PFD. Starting from the third day after modeling, the drug was given once a day by gavage for 4 weeks, and then all rats were put into metabolic cages (one per cage) for urine sample collection. Blood was collected from the abdominal aorta and then serum was extracted. All the samples were stored at −80 °C for later analysis.

### 2.3 16 S rRNA gene sequencing and analysis

16S rRNA gene sequencing was conducted by Majorbio Bio-Pharm Technology Co., Ltd. (Shanghai, China). Microbial genomic DNA was extracted from fecal samples using the PF Mag-Bind Soil DNA Kit (Omega Bio-Tek, Inc., Norcross, GA, United States), and DNA quality and concentration were assessed by 1% agarose gel electrophoresis and a NanoDrop^®^ ND-2000 spectrophotometer (Thermo Scientific, Inc., United States). The V3–V4 region of the bacterial 16S rRNA gene was amplified with barcoded primers 338F (5′-ACTCCTACGGGAGGCAGCAG-3′) and 806R (5′-GGACTACHVGGGTWTCTAAT-3′).(Liu et al., 2016) PCR products were purified, quantified using a Synergy HTX (BioTek Instruments, Inc., Winooski, VT, United States), and used for library preparation. Sequencing was performed on the Illumina NextSeq 2000 PE300 platform (Illumina, Inc., San Diego, CA, United States).

Raw sequencing data were processed on the Majorbio Cloud Platform,^1^ including demultiplexing, quality filtering, merging, and clustering into operational taxonomic units (OTUs) at 97% similarity using Usearch 11 (Stackebrandt and Goebel, 1994; Edgar, 2013). Taxonomic assignment was conducted using the RDP Classifier version 2.13 against the Silva v138 database (Wang et al., 2007). Functional profiles were predicted using PICRUSt2 (Douglas et al., 2020).

### 2.4 Metabolomics analysis

#### 2.4.1 Preparation of serum and urine samples

Each serum or urine sample was precipitated with three volumes of methanol. After vortexing for 3 min, the sample was left at −20 °C for 1 h, and then centrifuged (High-speed tabletop refrigerated centrifuge, 5804R, Eppendorf, Hamburg, Germany) at 13,000 rpm for 15 min. The supernatant was taken for analysis by the UPLC-Q-Exactive Orbitrap MS. The quality control (QC) sample was prepared by mixing equal aliquots from all serum samples and all urine samples, respectively. Five QC injections were run at the beginning of the batch for system equilibration, followed by one QC injection after every five sample injections to monitor instrument stability and analytical reproducibility.

#### 2.4.2 UPLC-Q-Exactive Orbitrap MS conditions

Mobile phase for chromatographic separation: (A) acetonitrile and (B) 0.1% formic acid. Elution program for serum: 0–2 min, 98% B; 2–3 min, from 98% B down to 60% B; 3–7 min, from 60% B down to 49% B; 7–11 min, from 49% B down to 40% B;11–18 min, 40% B; 18–22 min, from 40% B down to 2% B; and 22–24 min, 2% B. Elution program for urine: 0–2 min, 98% B; 2–3 min, from 98% B down to 80% B; 3–9 min, 80% B; 9–10 min, from 80% B down to 52% B;10–13 min, from 52%B down to 45% B; 13–20 min, from 45% B down to 37% B; and 20–23 min, from 37% B down to 0% B. Chromatographic separations were conducted on a Thermo Acclaim™ RSLC 120 C18 (2.1 mm × 100 mm, 2.2 μm, Thermo Fisher Scientific, Sunnyvale, CA, United States) for reversed phase separation. Injection volume, 3 μL; flow rate, 0.3 mL/min; and column temperature, 40 °C. Mass detection data were obtained both in positive and negative modes. Ion spray voltage, 3.8 kV (+) and 3.0 kV (−); capillary temperature, 350 °C; Sheath gas, 45 arb; Aux gas, 10 arb; S-Lens RF Level, 50; and mass range, 100–1,500 m/z.

#### 2.4.3 Metabolomics data analysis

Metabolites annotation of the MS data was performed with the Compound Discoverer program 3.1 (Thermo Fisher Scientific, San Jose, CA, United States). Pretreated data were uploaded into SIMCA-P (version 14.1, Umetrics, Umea, Sweden) for multivariate data analysis. Principal component analysis (PCA) and orthogonal projections to latent structures (OPLS-DA) were used to observe the similarities and differences between the groups.

Two hundred of recalculated permutations were used to evaluate the validity of the model. Differentially expressed metabolites (DEMs) between two groups were screened with the criteria of variable importance in projection (VIP) > 1, *p* < 0.05, and |log_2_(FC)| > 1. Subsequently, the human metabolic database (HMDB)^2^ and MzCloud^3^ were used for identification of DEMs, and the metaboanalyst^4^ was used for analysis of metabolic pathways.

The chemical structures of reversed DEMs were retrieved from the PubChem database^5^ in the form of SMILES strings. These were subsequently submitted to the SwissTargetPrediction platform^6^ to predict potential protein targets based on chemical similarity. The predicted targets were then subjected to pathway enrichment analysis using the Metascape platform,^7^ with a particular focus on the KEGG pathway database, to elucidate the biological pathways potentially modulated by the reversed DEMs.

### 2.5 Statistical analysis

Multivariate analysis of metabolomic data was conducted using SIMCA software. For gut microbiota data, two-group comparisons were performed using the two-tailed Wilcoxon rank-sum test with false discovery rate (FDR) correction. Multiple-group comparisons were analyzed using the Kruskal–Wallis test followed by FDR adjustment, and *post hoc* pairwise differences were assessed with Dunn’s test. Statistical significance was defined as *p* < 0.05. All experiments were conducted with six biological replicates (*n* = 6).

## 3 Results

### 3.1 Chemical components identification of JBHD

The chemical profile of JBHD was analyzed by UPLC-Q-Exactive Orbitrap MS using both the positive and negative ion modes (Supplementary Figure 1). In total, 30 major compounds (Supplementary Table 1) were identified by comparing them with authentic standards. The identified compounds were primarily classified into flavonoids (10 compounds: hydroxysafflor yellow A, calycosin 7-O-glucoside, hyperoside, ononin, calycosin, epimedin A, epimedin B, icariin, formononetin, and baohuoside I), terpenoids (7 compounds: oxypaeoniflorin, albiflorin, paeoniflorin, galloylpaeoniflorin, astragaloside IV, astragaloside II, and astragaloside I), organic acids (5 compounds: gallic acid, neochlorogenic acid, chlorogenic acid, cryptochlorogenic acid, and ferulic acid), nucleosides (3 compounds: adenine, hypoxanthine, and guanosine), phthalides (3 compounds: senkyunolide H, ligustilide, and levistolide A), cyanogenic glycosides (1 compound: amygdalin), phenolic glycosides (1 compound: curculigoside), reflecting the chemical diversity of JBHD.

### 3.2 JBHD intervention for gut microbiota modulation in IPF rats

#### 3.2.1 JBHD intervention restored gut microbiota balance and gut microbiota health

**FIGURE 1:**
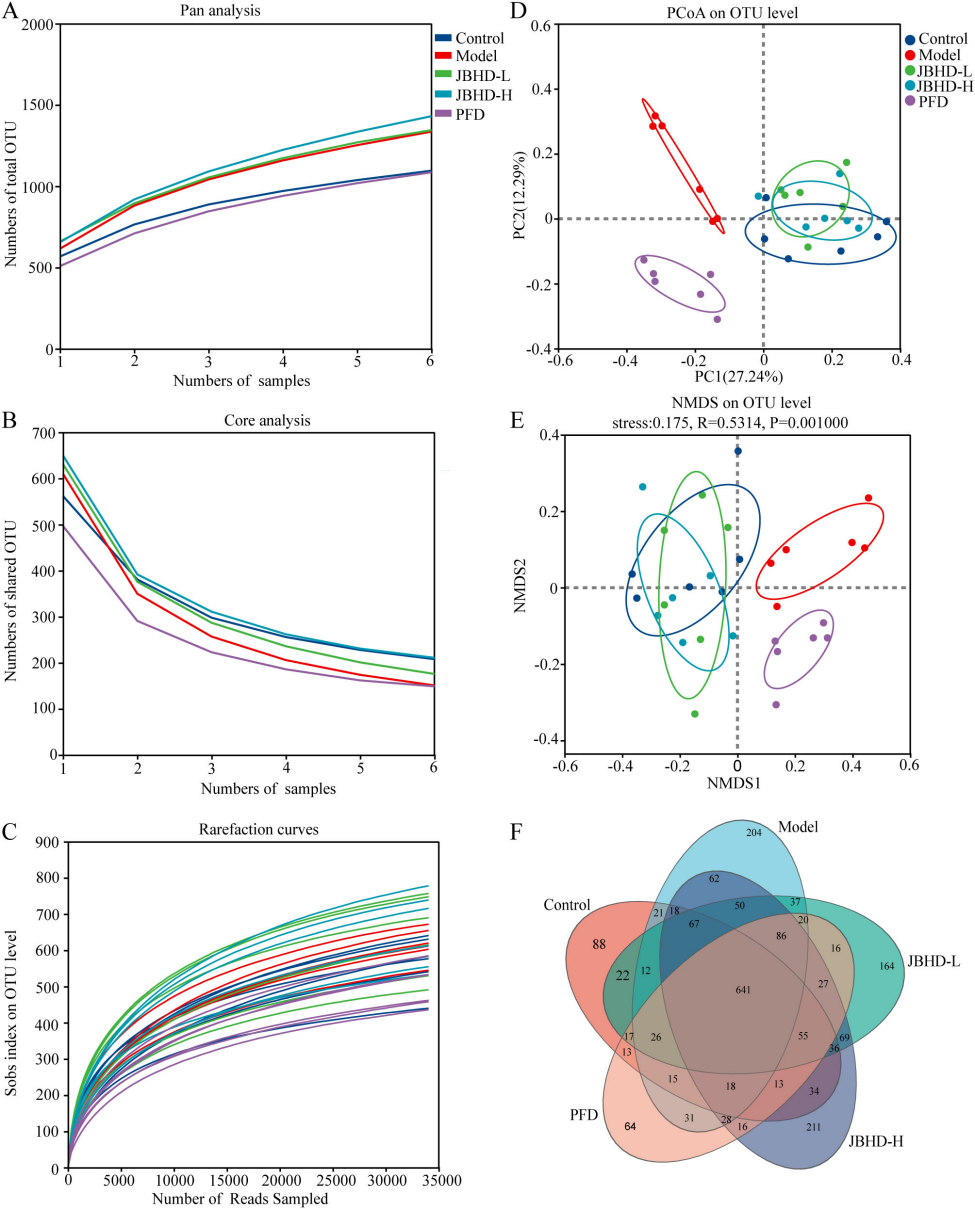
Comprehensive microbiota profiling based on OTU-level metrics (*n* = 6). **(A)** Pan analysis. **(B)** Core analysis. **(C)** Rarefaction curves. **(D)** PCoA analysis. **(E)** NMDS analysis, and **(F)** OTU compositional comparison among groups via Venn diagram. JBHD-L and JBHD-H, JiaweiBuyangHuanwu Decoction at low (10 g/kg) and high (20 g/kg) doses, respectively; PFD, Pirfenidone capsules.

16S rRNA sequencing was employed to detect changes in the gut microbiota among the Control, Model, JBHD-L, JBHD-H, and PFD groups. A total of 1 Domain, 1 Kingdom, 16 Phyla, 22 Classes, 53 Orders, 89 Families, 203 Genera, 399 Species, and 2,181 OTUs were identified. The pan and core OTU analyses were employed to evaluate the expansion of total species richness and the stabilization of the core microbiota composition with increasing sample size. The sequencing curve plateaued (Figures 1A,B), indicating that the sampling depth was sufficient to recover the full range of species present in the samples. Rarefaction curves based on the Sobs approached saturation for all samples (Figure 1C), indicating that the sequencing depth was sufficient to capture the microbial diversity within each sample. Alpha diversity indices (Ace, Chao, Shannon, Simpson, Sobs, and Coverage) showed no significant differences between groups (Supplementary Figure 2). Beta diversity analysis was employed to assess similarities and differences in overall community structure among groups. Among these analyses, PCoA (Figure 1D) revealed a clear separation between the Model and Control, while JBHD-L and JBHD-H partially overlapped with the Control, indicating that the gut microbial community structure was markedly altered in IPF rats and that JBHD treatments tended to restore it toward a normal state. Similarly, NMDS (Figure 1E) revealed a distinct separation between the Model and Control groups, with JBHD-treated groups positioned closer to the Control in ordination space, indicating a partial restoration of gut microbiota structure by JBHD. The stress value of 0.175 indicated an acceptable level of ordination reliability.

**FIGURE 2:**
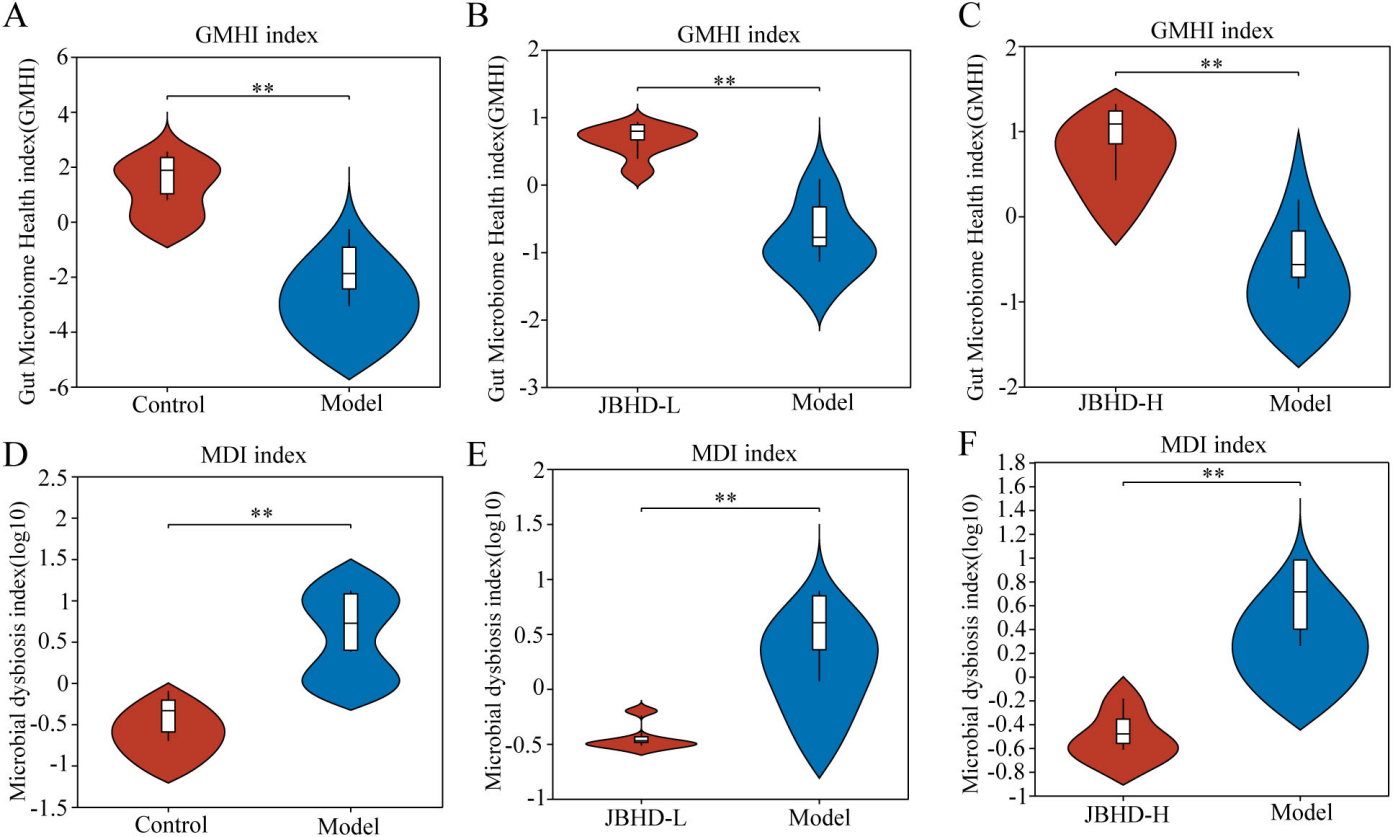
Comparative analysis of GMHI and MDI (*n* = 6). **(A–C)** GMHI comparisons and **(D–F)** MDI comparisons of Model vs. Control, JBHD-L vs. Model, and JBHD-H vs. Model, respectively. **p < 0.01 vs. Model group. JBHD-L and JBHD-H, JiaweiBuyangHuanwu Decoction at low (10 g/kg) and high (20 g/kg) doses, respectively.

The GMHI, a robust metric based on species-level taxonomic features of gut microbiota samples, is widely regarded as an indicator of gut microbiota health and dysbiosis. As shown in Figures 2A–C, the GMHI of the Model was significantly lower than that of the Control, while it was notably elevated in the JBHD-L and JBHD-H, suggesting that GMHI effectively distinguished between the Model and Control, and that JBHD treatments improved gut microbiome health in IPF rats. The MDI, which quantifies the extent of microbial imbalance, increases with the severity of dysbiosis (Figures 2D–F). The Model exhibited a significantly higher MDI compared to the Control, whereas JBHD treatments markedly reduced the MDI levels. These findings indicate that JBHD effectively alleviated gut microbial dysbiosis in IPF rats.

### 3.2.2 JBHD intervention altered microbial community composition

Venn diagram analysis was performed to assess differences in OTU distribution among the five groups. A total of 641 OTUs were shared across all groups. Specifically, the Control, Model, JBHD-L, JBHD-H, and PFD groups contained 88, 204, 164, 211, and 64 unique OTUs, respectively. Notably, JBHD-H exhibited the highest OTU richness, with 1,431 OTUs identified (Figure 1F). Supplementary Figure 3 shows the taxonomic composition of the Control, Model, JBHD-L, JBHD-H, and PFD groups, showing the top 10 phyla, top 20 genera, and top 20 species in terms of relative abundance.

**FIGURE 3:**
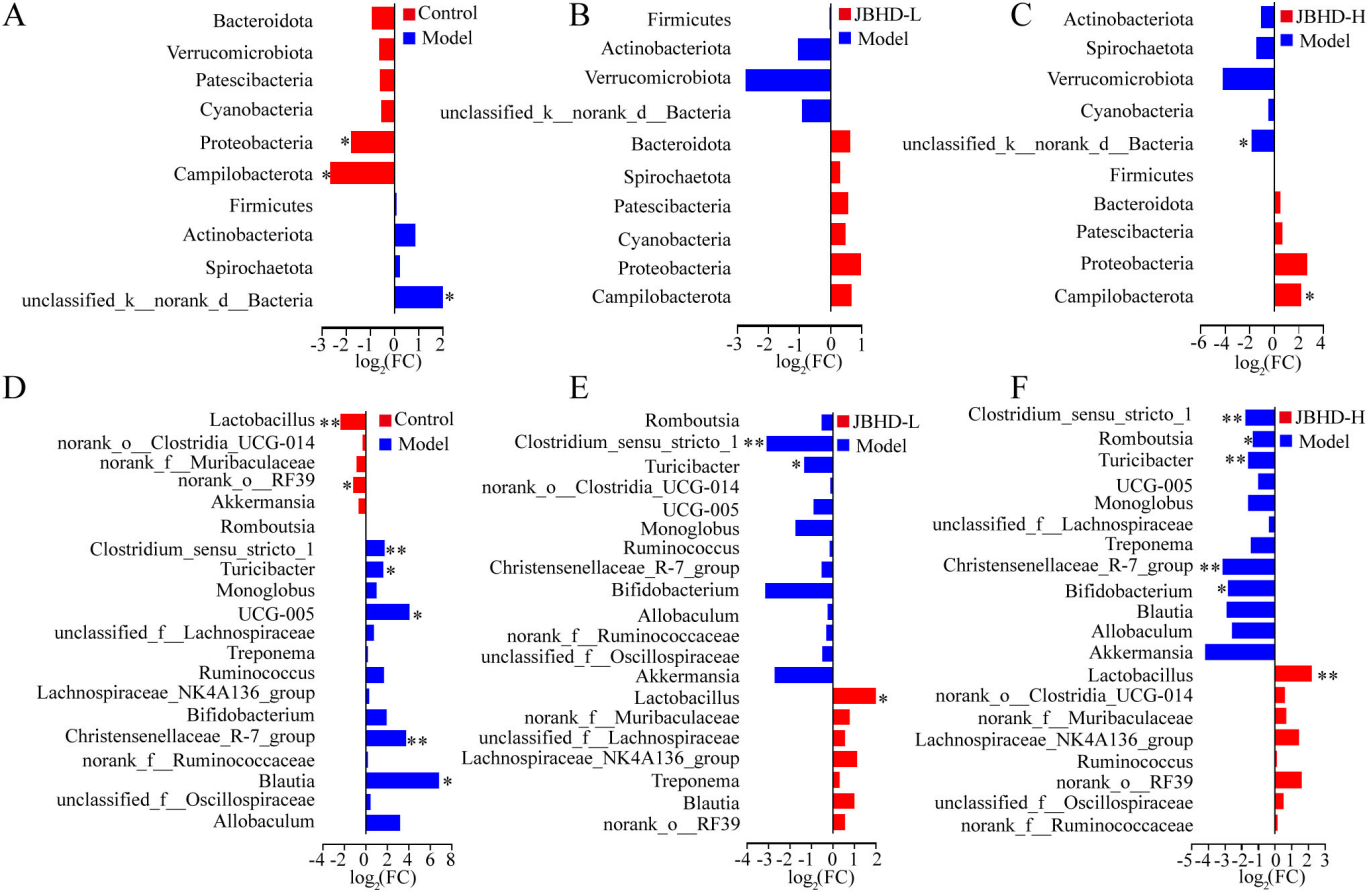
Differential analyses of the gut microbiota (*n* = 6). **(A–F)** Differential abundance comparisons at phylum and genus levels, respectively. *p < 0.05 and **p < 0.01 vs. Model group; log_2_(FC), log_2_(Fold Change); JBHD-L and JBHD-H, JiaweiBuyangHuanwu Decoction at low (10 g/kg) and high (20 g/kg) doses, respectively.

Differentially altered fecal microbes (DAFMs) were subsequently defined as microbial taxa with significant abundance differences between Model vs. Control, JBHD-L vs. Model, or JBHD-H vs. Model, at the phylum, genus, and species levels. At the phylum level, DAFMs included unclassified k__norank d__Bacteria, which was enriched in the Model, and Campilobacterota and Proteobacteria, both of which were reduced (Figure 3A). JBHD-L (Figure 3B) did not induce statistically significant change, whereas JBHD-H intervention (Figure 3C) notably reversed unclassified k__norank d_Bacteria and Campilobacterota, indicating a potential restorative effect. At the genus level, DAFMs comprised depleted *Lactobacillus* and norank o__RF39, alongside enriched *Clostridium* sensu stricto 1, *Turicibacter*, UCG-005, Christensenellaceae R-7 group and *Blautia* in the Model (Figure 3D). Both JBHD-L and JBHD-H significantly reversed the abundance of *Lactobacillus*, *Clostridium* sensu stricto 1, and *Turicibacter*, while JBHD-H also reversed the abundance of Christensenellaceae R-7 group (Figures 3E,F). Species-level differences of DAFMs among groups were illustrated in Supplementary Figure 4. Notably, JBHD reversed the abundance of *Lactobacillus murinus*, *Lactobacillus johnsonii*, uncultured bacterium g__*Turicibacter*, and uncultured bacterium g__*Clostridium* sensu stricto 1.

Collectively, these findings demonstrate that JBHD-H exerted a more pronounced regulatory effect on the gut microbiota than JBHD-L, particularly through the reversal of DAFMs, which may underlie its therapeutic efficacy by rebalancing the intestinal microbial ecosystem.

#### 3.2.3 JBHD intervention induced microbial biomarkers and functional alterations

**FIGURE 4:**
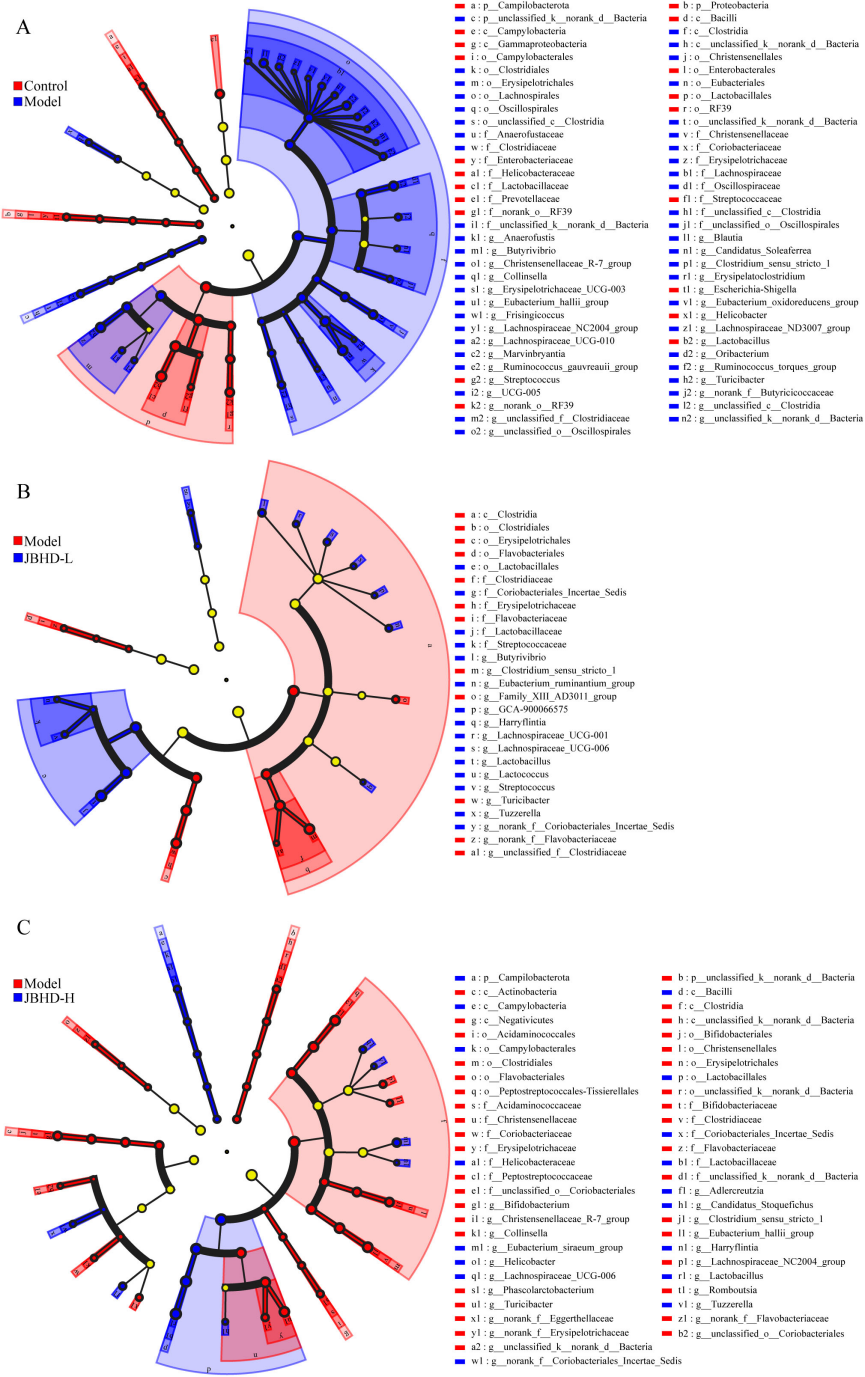
LEfSe analysis of gut microbiota (*n* = 6). **(A)** Model vs. Control, **(B)** JBHD-L vs. Model, and **(C)** JBHD-H vs. Model. Nodes in different colors represent microbial taxa that are significantly enriched in the corresponding groups and contribute notably to intergroup differences. Pale yellow nodes indicate taxa that show no significant differences among groups or have minimal impact on the observed group separation. JBHD-L and JBHD-H, JiaweiBuyangHuanwu Decoction at low (10 g/kg) and high (20 g/kg) doses, respectively.

LEfSe (Linear discriminant analysis effect size) analysis was conducted to identify microbial taxa exhibiting significant differences in relative abundance across multiple taxonomic levels, from phylum to genus, between the Model and the Control, as well as between the JBHD and the Model. This method not only enables hierarchical identification of discriminative taxa, but also quantifies their contribution to group differentiation through LDA scores. Taxa with high LDA values may play critical roles in disease development and progression, and thus serve as potential biomarkers that distinguish the respective comparison groups. Figure 4 shows the results of the LEfSe analysis, which identified 67 differential taxa between the Control and Model groups (Figure 4A), 27 between Model and JBHD-L (Figure 4B), and 54 between Model and JBHD-H (Figure 4C), at LDA > 2.5. Notably, unclassified k__norank d__Bacteria, *Turicibacter*, *Clostridium* sensu stricto 1, Christensenellaceae R-7 group, *Collinsella*, Lachnospiraceae NC2004 group, and *Eubacterium hallii* group were enriched in Model, whereas Campilobacterota, *Lactobacillus*, *Helicobacter*, *Streptococcus*, *Eubacterium ruminantium* group, Lachnospiraceae UCG-006, *Butyrivibrio*, Lachnospiraceae UCG-001, *Tuzzerella*, GCA-900066575, *Lactococcus*, *Harryflintia*, *Adlercreutzia*, Candidatus *Stoquefichus*, and *Eubacterium siraeum* group were more abundant in the two JBHD groups.

**FIGURE 5:**
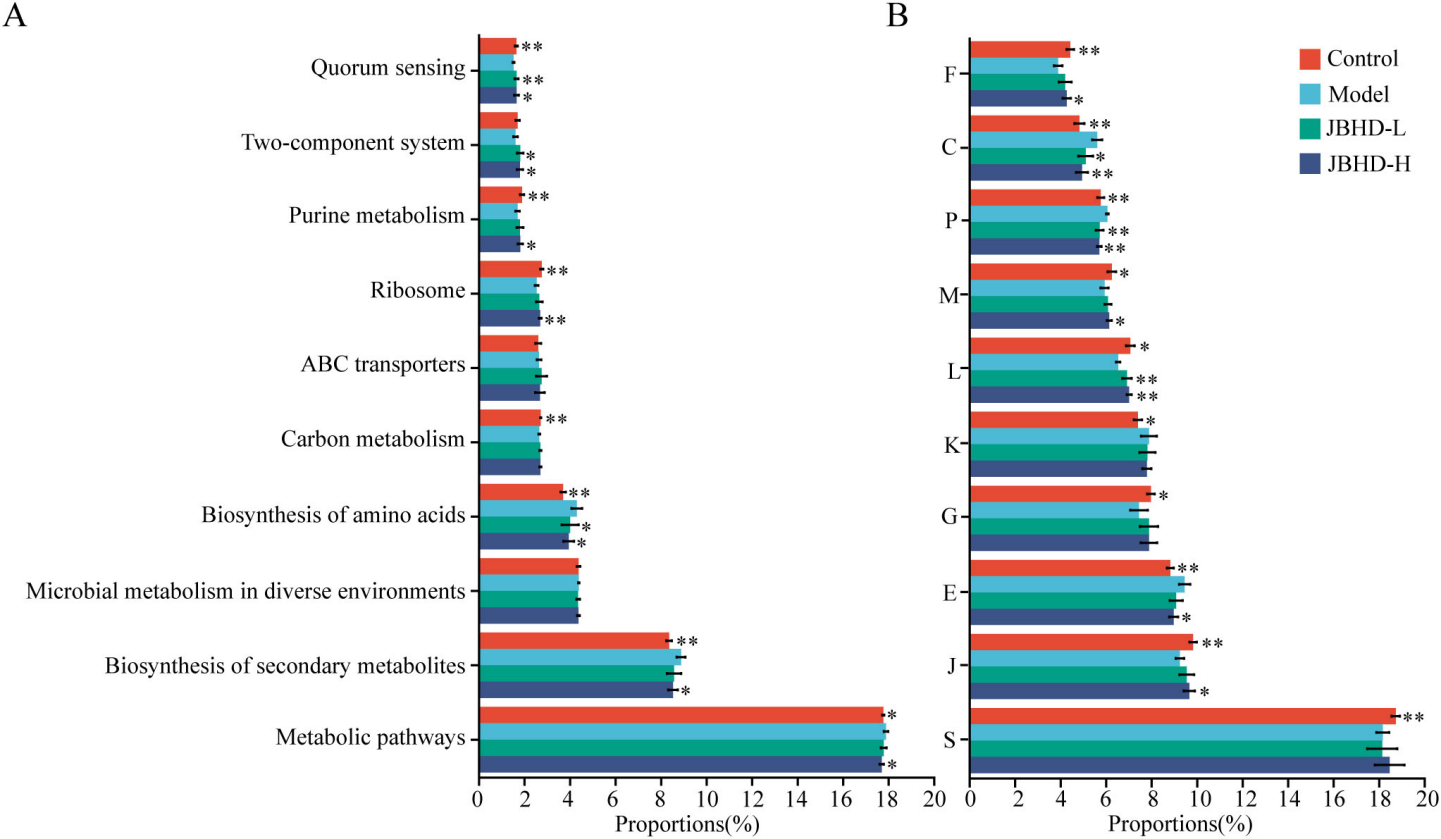
Predicted microbial functional profiles based on 16S rRNA gene sequencing using KEGG and COG annotations (*n* = 6). **(A)** KEGG pathways annotation at Level 3. **(B)** Functional annotation at the function level of the COG database. *p < 0.05 and **p < 0.01 vs. Model group. F, Nucleotide transport and metabolism; C, Energy production and conversion; P, Inorganic ion transport and metabolism; M, Cell wall/membrane/envelope biogenesis; L, Replication; recombination and repair; K, Transcription; G, Carbohydrate transport and metabolism; E, Amino acid transport and metabolism; J, Translation; ribosomal structure and biogenesis; S, Function unknown. Data were shown as mean ± SD. JBHD-L and JBHD-H, JiaweiBuyangHuanwu Decoction at low (10 g/kg) and high (20 g/kg) doses, respectively.

Based on 16S rRNA gene sequencing data, microbial functional profiles were inferred using PICRUSt2, with annotations mapped to both KEGG and COG databases to predict potential microbial functions. Figure 5A illustrates the top 10 abundant functions predicted at KEGG Level 3, revealing IPF-associated disruptions in multiple pathways. JBHD-L reversed functions related mainly to Biosynthesis of amino acids and Quorum sensing, whereas JBHD-H modulated a broader range of pathways, including Metabolic pathways, Biosynthesis of secondary metabolites, Biosynthesis of amino acids, Ribosome, Purine metabolism, and Quorum sensing. At the COG functional level (Figure 5B), JBHD administration significantly modulated microbial gene functions compared with the Model group, in which JBHD-L mainly affected categories L, P, and C, while JBHD-H modulated categories J, E, L, P, C, M, and F. Taken together, JBHD-H demonstrated a wider regulatory range than JBHD-L, highlighting the potential of JBHD to alleviate IPF through the reprogramming of microbial functional pathways.

### 3.3 JBHD intervention for metabolic modulation in IPF rats

#### 3.3.1 JBHD intervention modulated metabolic signatures in serum and urine

**FIGURE 6:**
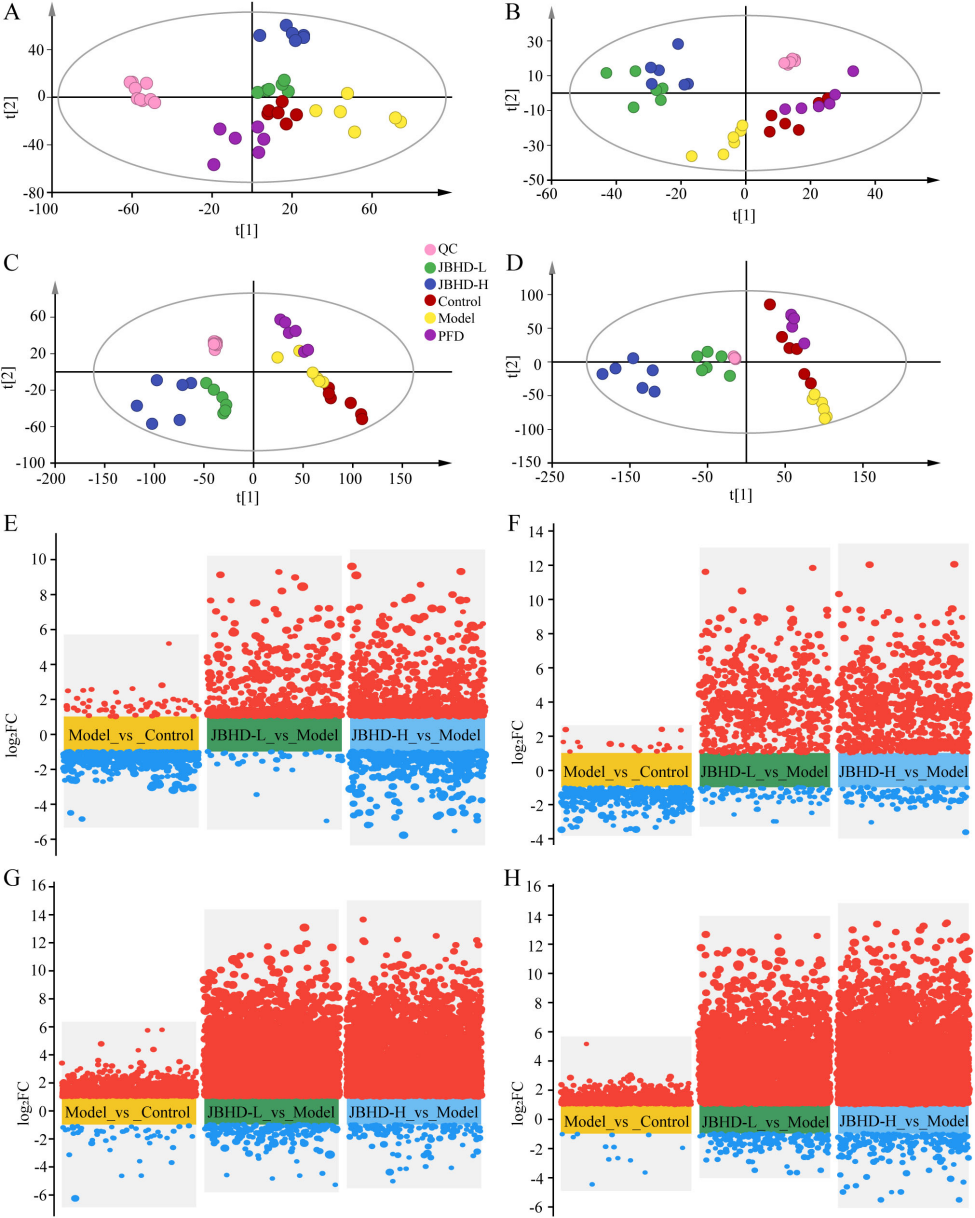
Multivariate statistical analysis and volcano plots of serum and urine metabolomics (*n* = 6). **(A,B)** PCA of serum samples in positive and negative ion modes, respectively. **(C,D)** PCA of urine samples in positive and negative ion modes, respectively. **(E,F)** Volcano plots of DEMs in serum under positive and negative ion modes, respectively. **(G,H)** Volcano plots of DEMs in urine under positive and negative ion modes, respectively. DEMs, differentially expressed metabolites; JBHD-L and JBHD-H, JiaweiBuyangHuanwu Decoction at low (10 g/kg) and high (20 g/kg) doses, respectively; PFD, Pirfenidone capsules.

Metabolomics studies of serum and urine were performed using UPLC-Q-Exactive Orbitrap MS technique, with one quality control (QC) sample inserted every five samples throughout the injection process to evaluate stability. Total ion chromatograms of QC samples are shown in Supplementary Figure 5. As shown in Figures 6A–D, all the principal component analysis (PCA) data show good separation among the five groups (i.e., Control, Model, JBHD-L, JBHD-H, and PFD), with excellent clustering within each group in both serum (Figures 6A,B) and urine samples (Figures 6C,D). Concurrently, the QC samples were also closely clustered together, strongly implying that the UPLC-Q-Exactive Orbitrap MS system stability was satisfactory.

In the metabolic profiles of serum samples (Figures 6A,B), the Model and Control groups were clearly separated, and the JBHD-L and JBHD-H groups exhibited greater divergence from the Model, suggesting their role in regulating the metabolic profile had already become apparent in serum. In the metabolic profiles of urine samples (Figures 6C,D), although the Model and Control were separated not as clearly as those in the serum samples, the JBHD-L and JBHD-H exhibited even clearer separation from the Model in both the positive and negative modes, indicating that their role in regulating the metabolic profile had become more obvious in urine. All the above PCA data demonstrate that JBHD significantly altered the metabolic profile of serum and urine in IPF rats.

Furthermore, OPLS-DA analyses, including OPLS-DA score plots, their permutation tests, and S-plots, were carried out to explore notable metabolic differences in serum (Supplementary Figure 6) and urine (Supplementary Figure 7). The OPLS-DA score plots showed that the metabolites between the Model vs. Control, JBHD-L vs. Model, and JBHD-H vs. Model group pairs were remarkably different both in the positive and negative ion modes. Permutation testing showed that the original R^2^ and Q^2^ values were substantially higher than those from the permuted models, with R^2^ consistently exceeding Q^2^. Together with the negative Y-intercept of Q^2^, these results confirmed the absence of overfitting and supported the model’s predictive reliability. S-plots derived from OPLS-DA models in both positive and negative ion modes were further analyzed to intuitively identify variables contributing significantly to classification, and they clearly demonstrated that variables at both ends of the distribution could be considered as important candidate biomarkers. Additionally, VIP scores derived from the OPLS-DA models were used to quantitatively evaluate each metabolite’s contribution to inter-group separation. A threshold of VIP > 1 was applied to identify metabolites with significant discriminatory power.

Finally, the above data also demonstrated that the Model was significantly different from the Control, and the JBHD-L and JBHD-H were apparently separated from the Model. All the developed OPLS-DA models in this work were stable, reliable, and free from overfitting.

#### 3.3.2 JBHD intervention affected both DEMs and reversed DEMs in serum and urine

Volcano plots (Figures 6E–H) visualized the overall distribution of various individual metabolites identified. Subsequently, VIP scores were used to assess the importance of DEMs, with VIP > 1, *p* < 0.05, and |log_2_(FC)| > 1 considered the criteria for screening the DEMs between a pair of groups for comparison. log_2_(FC) > 1 indicated an upward adjustment, and log_2_(FC) < −1 indicated a downward adjustment. The identified DEMs were listed in Supplementary Tables 2, 3.

In serum samples, 40, 32, and 34 DEMs in the pairs of Model *vs.* Control, JBHD-L *vs.* Model, and JBHD-H *vs.* Model were identified, and Lipids and lipid-like molecules and Organic acids and derivatives were the top-two superclasses in all the three comparison pairs, accounting for 80.00, 81.25, and 82.36% of the total, respectively (Supplementary Table 4). In urine samples, 62, 40, and 56 DEMs were identified in the Model vs. Control, JBHD-L vs. Model, and JBHD-H vs. Model pairs. These DEMs were predominantly concentrated in Organic acids and derivatives together with Organoheterocyclic compounds in the Model vs. Control and JBHD-H vs. Model pairs, and in Organic acids and derivatives together with Benzenoids in the JBHD-L vs. Model pair, accounting for 62.90, 50.00, and 52.50% of the total, respectively (Supplementary Table 5). Additionally, Benzenoids constituted 25.00 and 19.64% of the DEMs in the Model vs. JBHD-L and Model vs. JBHD-H pairs, respectively, further implying that Benzenoids represented a significant category of DEMs with the potential to facilitate therapeutic outcomes in IPF by the JBHD. Overall, a greater number of DEMs were detected in the JBHD-H vs. Model comparison than in the JBHD-L vs. Model comparison, particularly in urine, where JBHD-H yielded 16 additional DEMs relative to JBHD-L.

Furthermore, the reversed DEMs in serum and urine, defined as DEMs that underwent a significant change following bleomycin treatment in the Model and subsequently reversed to normal following JBHD intervention, were identified for special attention. As shown in Table 1, 11 and 13 DEMs were determined as reversed DEMs in serum and urine, respectively. In serum, JBHD could up-regulate 4 and down-regulate 7 reversed DEMs. In urine, JBHD could up-regulate 4 and down-regulate 9 reversed DEMs. The results of reversed DEMs suggested that the JBHD intervention was able to influence metabolism both in blood and urine. Combined analysis of the reversed DEMs in serum and urine (Table 1) revealed that JBHD exerted its effects on IPF rats through regulating Lipids and lipid-like molecules, Organic acids and derivatives, Organoheterocyclic compounds, Benzenoids, Nucleosides, nucleotides, and analogs, and Organic oxygen compounds.

**TABLE 1:** The reversed DEMs by JBHD in serum and urine of the IFP rats.

No.	Super class	Compound name	Model vs. control			JBHD-L vs. model			JBHD-H vs. model		
			log_2_(FC)/*p*/VIP			log_2_(FC)/*p*/VIP			log_2_(FC)/*p*/VIP		
**A: serum**											
1	Lipids and lipid-like molecules	LPC	1.33	2.64E-02	1.08	−1.31	3.42E-03	1.18	/	/	/
2		Platelet-activating factor	1.28	2.26E-04	6.91	−1.30	8.98E-03	4.04	−1.73	4.21E-06	12.85
3		Thromboxane B2	−1.29	3.27E-02	2.68	1.19	3.53E-03	1.89	/	/	/
4		Muricholic acid	1.04	3.17E-02	3.30	−1.07	1.55E-02	1.83	−1.47	1.33E-02	3.49
5		7-ketodeoxycholic acid	−1.92	1.92E-02	2.30	1.40	2.24E-03	1.27	1.67	1.57E-02	1.53
6		Cholic acid	1.17	1.54E-02	1.68	/	/	/	−1.12	1.24E-02	14.82
7		Deoxycholic acid	1.42	7.66E-03	1.05	−1.31	3.00E-02	1.88	−1.49	8.66E-03	1.15
8		Stearic acid	−2.06	4.62E-02	1.16	/	/	/	1.46	3.63E-04	1.47
9	Organic acids and derivatives	Citrulline	−1.25	3.63E-04	1.59	1.12	4.51E-05	2.05	1.37	1.86E-05	1.65
10		*L*-norleucine	1.12	3.36E-01	2.17	−1.28	2.44E-02	2.77	−1.31	1.82E-04	8.06
11	Organoheterocyclic compounds	Dihydrothymine	1.55	4.81E-04	1.15	−1.43	2.12E-03	1.03	/	/	/
**B: urine**											
1	Organoheterocyclic compounds	Pyridoxal	−1.20	1.47E-03	1.88	/	/	/	1.05	4.45E-04	1.23
2		Xanthurenic acid	1.23	3.91E-02	1.88	−1.47	4.54E-04	1.35	−1.13	5.40E-04	1.22
3		Uric acid	−1.57	1.12E-02	4.63	1.22	1.13E-06	1.52	1.15	4.82E-06	3.00
4		9-methyluric acid	1.41	2.43E-02	2.30	/	/	/	−1.06	1.85E-06	1.44
5		Xanthurenic acid 8-O-sulfate	1.26	8.36E-05	3.82	−1.02	6.83E-05	1.39	/	/	/
6		5-hydroxyindoleacetic acid	2.49	9.09E-03	1.27	−1.96	5.42E-06	1.55	−1.39	4.62E-07	1.61
7	Nucleosides, nucleotides, and analogs	3’-AMP	1.04	1.33E-02	1.09	−1.78	2.85E-04	1.02	/	/	/
8		Pseudouridine	1.23	1.37E-03	2.95	−1.22	1.48E-06	1.70	−1.62	9.54E-08	1.59
9	Organic oxygen compounds	*N*-acetylgalactosamine 4-sulfate	1.42	2.19E-02	3.13	−1.91	1.15E-07	3.41	−1.41	3.79E-07	1.20
10		*N*-acetylneuraminic acid	1.02	8.40E-04	2.23	−1.17	3.72E-08	1.59	/	/	/
11	Organic acids and derivatives	Homovanillic acid sulfate	1.00	3.40E-03	8.65	/	/	/	−1.01	1.47E-06	3.17
12		L-tyrosine	−2.69	1.32E-02	1.44	1.47	5.85E-03	1.02	1.92	8.55E-04	1.01
13	Benzenoids	Gentisic acid	−1.19	3.16E-03	2.65	1.59	1.38E-06	3.46	2.39	1.36E-07	3.66

JBHD-L and JBHD-H, JiaweiBuyangHuanwu Decoction at low (10 g/kg) and high (20 g/kg) doses, respectively; DEMs, differentially expressed metabolites; LPC, LysoPC [22:5(7Z,10Z,13Z,16Z,19Z)/0:0].

#### 3.3.3 JBHD intervention impacted pathways of DEMs and reversed DEMs in serum and urine

**FIGURE 7:**
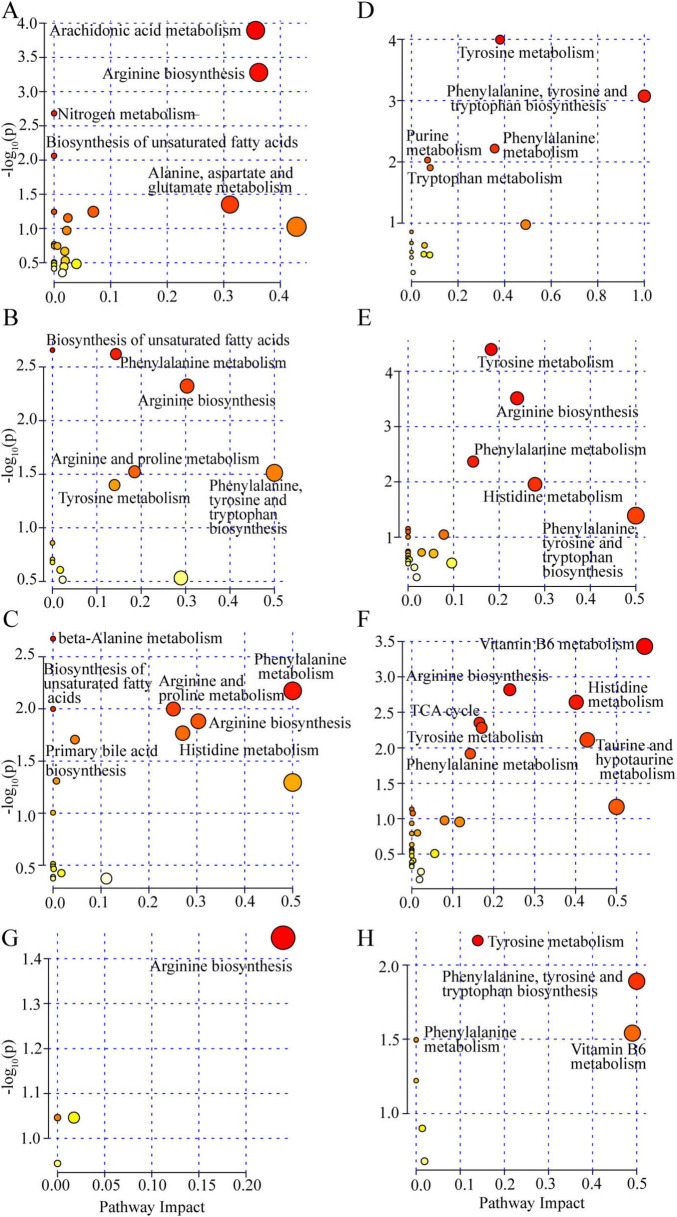
Enrichment analysis of metabolic pathways based on DEMs. **(A–C)** Significantly enriched serum metabolic pathways in the comparisons of Model vs. Control **(A)**, JBHD-L vs. Model **(B)**, and JBHD-H vs. Model **(C)**. **(D–F)** Significantly enriched urine metabolic pathways in the comparisons of Model vs. Control **(D)**, JBHD-L vs. Model **(E)**, and JBHD-H vs. Model **(F)**. **(G,H)** Key metabolic pathways associated with reversed DEMs in serum **(G)** and urine **(H)**. JBHD-L and JBHD-H, JiaweiBuyangHuanwu Decoction at low (10 g/kg) and high (20 g/kg) doses, respectively; DEMs, differentially expressed metabolites.

Figure 7 shows analysis of the metabolomics pathways conducted by MetaboAnalyst 6.0 for DEMs in serum and urine at the level of *p* < 0.05. In serum (Figures 7A–C), DEMs between the Model and Control groups were enriched in five pathways (Arachidonic acid metabolism, Arginine biosynthesis, Nitrogen metabolism, Biosynthesis of unsaturated fatty acids, and Alanine, aspartate and glutamate metabolism). Compared with Model, four pathways (Biosynthesis of unsaturated fatty acids, Phenylalanine metabolism, Arginine biosynthesis, and Arginine and proline metabolism) were commonly enriched based on the DEMs induced by both JBHD-L and JBHD-H, and two (Phenylalanine, tyrosine and tryptophan biosynthesis and Tyrosine metabolism) and three (Histidine metabolism, Primary bile acid biosynthesis and beta-Alanine metabolism) pathways specifically enriched by JBHD-L and JBHD-H, respectively. In the urine (Figures 7D–F), the DEMs in Model comparing to Control were enriched in five pathways (Tyrosine metabolism, Phenylalanine, tyrosine and tryptophan biosynthesis, Phenylalanine metabolism, Purine metabolism, and Tryptophan metabolism). Compared with Model, four pathways (Tyrosine metabolism, Arginine biosynthesis, Phenylalanine metabolism, Histidine metabolism) were commonly enriched based on the DEMs induced by the two JBHD treatments, one (Phenylalanine, tyrosine and tryptophan biosynthesis) by JBHD-L and three (Vitamin B6 metabolism, TCA cycle, and Taurine and hypotaurine metabolism) by JBHD-H.

Subsequently, further pathway analysis was conducted on the reversed DEMs identified in serum and urine. The pathways enriched for the reversed DEMs in serum was Arginine biosynthesis (Figure 7G), those in urine were Tyrosine metabolism, Phenylalanine, tyrosine and tryptophan biosynthesis, Vitamin B6 metabolism, and Phenylalanine metabolism (Figure 7H). The reversed metabolites in serum and urine were found to be enriched in completely different pathways, indicating that the various systems in the body had undergone different metabolic processes when the pathological state of IPF was present.

Clearly, JBHD intervention significantly influenced the metabolism of serum and urine, as evidenced by the enrichment of specific metabolic pathways associated with DEMs. Notably, DEMs identified in the JBHD-H vs. Model comparison were involved in a greater number of pathways than those in the JBHD-L vs. Model comparison. Overall, JBHD appears to exert a broad-spectrum effect on metabolic pathways, with a notable impact on Amino acid metabolism (Phenylalanine metabolism, Arginine biosynthesis, Phenylalanine, tyrosine and tryptophan biosynthesis, Arginine and proline metabolism, Tyrosine metabolism, Histidine metabolism), Lipid metabolism (Biosynthesis of unsaturated fatty acids, Primary bile acid biosynthesis, Glycerophospholipid metabolism), Metabolism of other amino acids (beta-Alanine metabolism), Metabolism of cofactors and vitamins (Vitamin B6 metabolism), Carbohydrate metabolism (TCA cycle), and Metabolism of other amino acids (Taurine and hypotaurine metabolism), which could have implications for understanding its therapeutic mechanisms.

**FIGURE 8:**
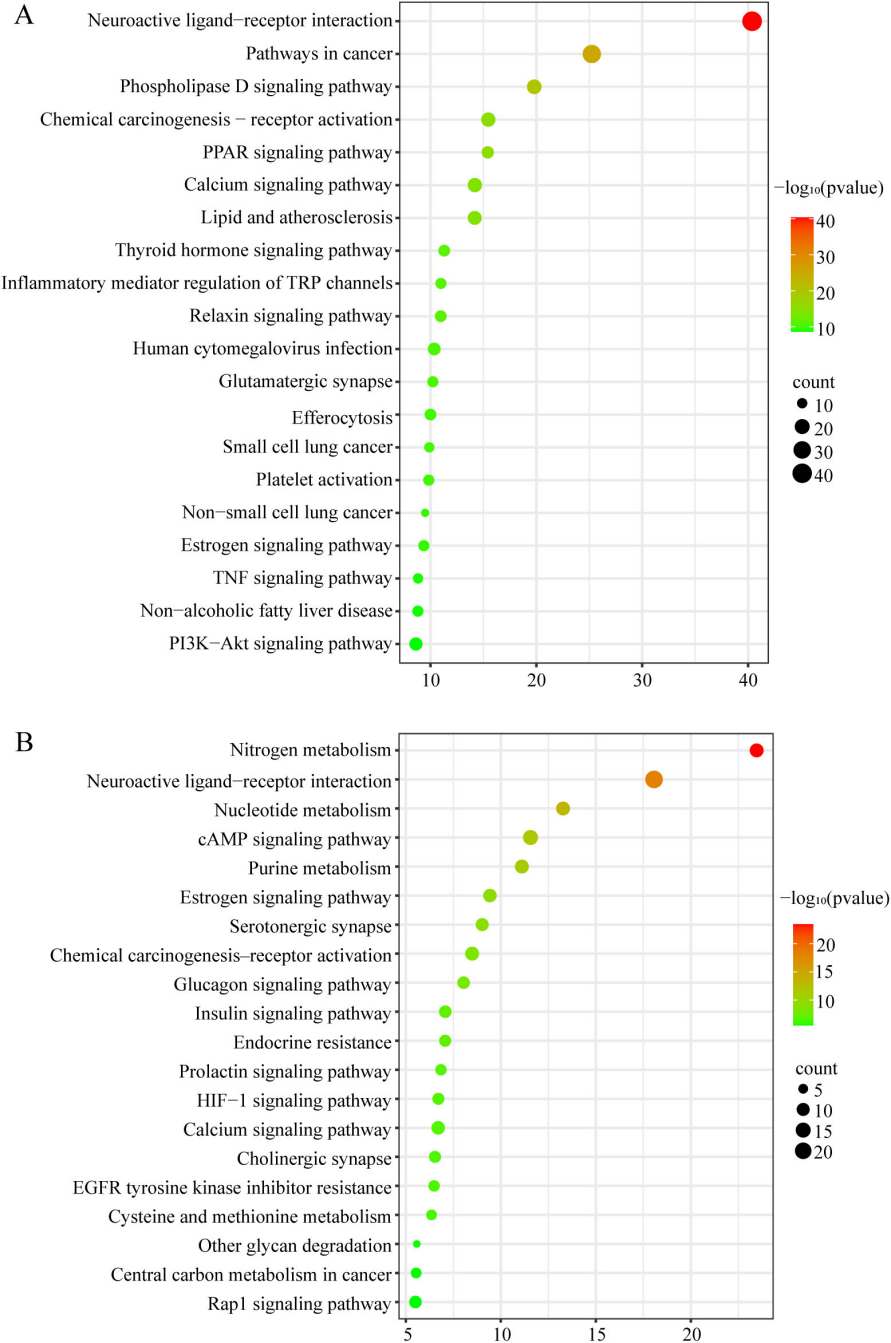
KEGG pathway enrichment analysis of the predicted targets of reversed DEMs in serum **(A)** and urine **(B)**. Dot color indicates statistical significance [- log_10_(*p*)], and dot size represents the number of associated genes.

Furthermore, KEGG pathway enrichment of the predicted targets of reversed DEMs in serum (Figure 8A) and urine (Figure 8B) via Metascape revealed potential signaling and regulatory pathways that they may influence from a functional perspective. The top 20 most significantly enriched pathways (*p* < 0.01) were visualized according to −log_10_(p) values. Notably, several shared pathways, including Neuroactive ligand–receptor interaction, Chemical carcinogenesis–receptor activation, Calcium signaling pathway, and Estrogen signaling pathway, are closely associated with immune regulation and pulmonary fibrosis. In addition, other key pathways involved in both immune processes and fibrotic progression included the TNF signaling pathway, PI3K-Akt signaling pathway, cAMP signaling pathway, and Purine metabolism.

### 3.4 Correlation and network analyses of DAFMs and DEMs in serum and urine

A Spearman correlation analysis for clustering was performed between the Model and Control groups of rats to explore the relationships between DAFMs (Figure 3D) at the genus level and DEMs in serum (Supplementary Table 2A) and urine (Supplementary Table 3A). The statistical significance of DAFMs and DEMs was screened based on the criteria of |r| > 0.5 and *p* < 0.05. As depicted in Supplementary Figure 8, significant correlations were observed between the 7 DAFMs and the majority of the DEMs, highlighting their substantial impact on the metabolic profile, which apparently resulted from bleomycin treatment. A total of 195 and 207 significant correlations were identified between the 7 DAFMs and 40 DEMs in serum (Supplementary Figure 8A) and 62 DEMs in urine (Supplementary Figure 8B), respectively.

**FIGURE 9:**
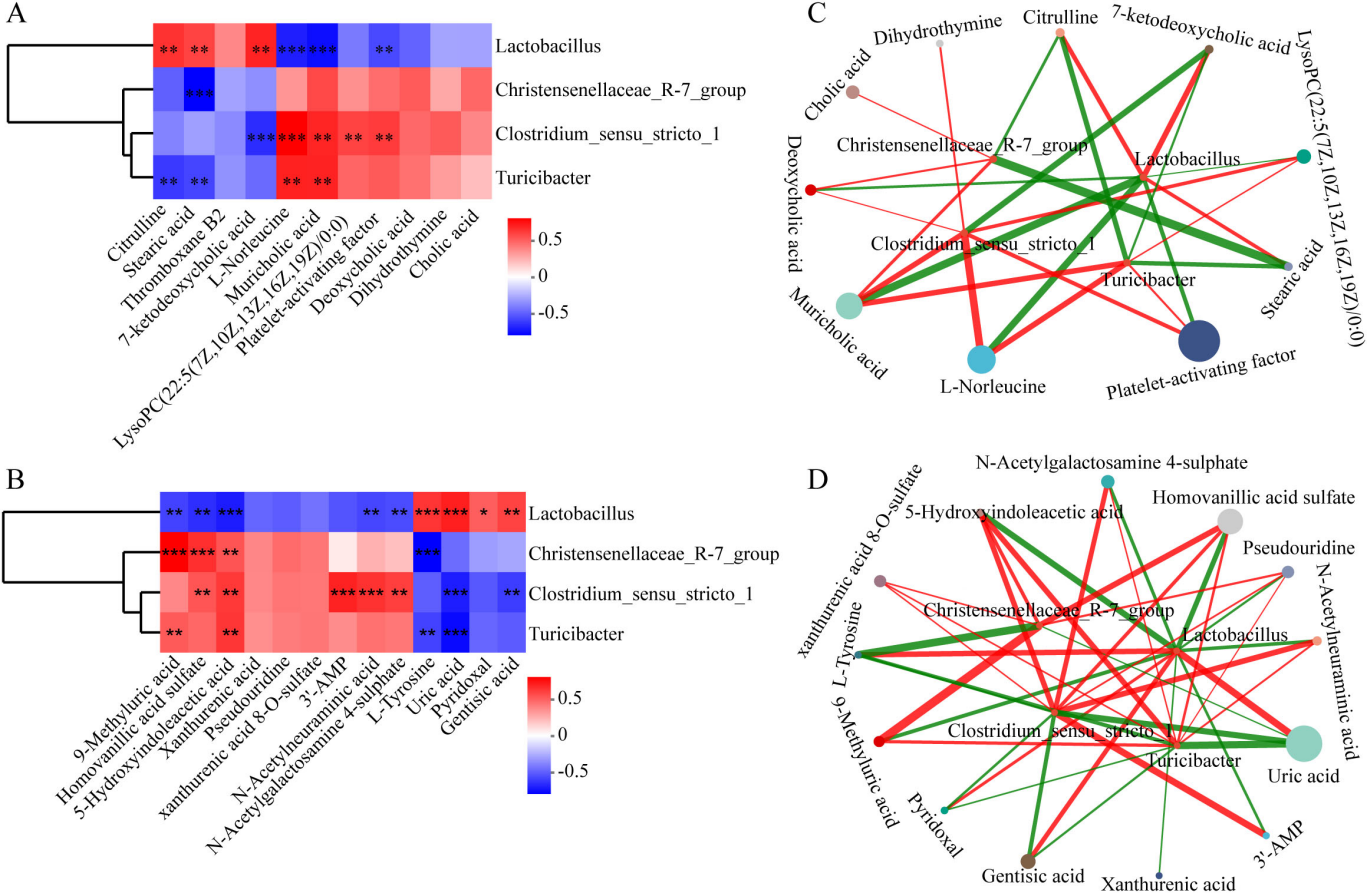
The Spearman correlation analysis on reversed DAMFs and reversed DEMs in serum **(A,C)** and urine **(B,D)**. **(A,B)** Red and blue indicate positive and negative correlations, respectively. *p < 0.05, **p < 0.01, ***p < 0.001. **(C,D)** Node size reflects the relative abundance of microbial genera or metabolite levels; node colors represent different microbial taxa or metabolite types; edge color denotes correlation direction (red: positive; green: negative), and edge thickness corresponds to the absolute value of Spearman’s correlation coefficient, nodes with more connections imply stronger interactions within the network.

The correlation analysis between DAFMs and DEMs revealed a strong connection between them in Model *vs.* Control. Therefore, a clustering Spearman correlation analysis was further performed on the reversed 4 DAFMs and the reversed 11 and 13 DEMs by JBHD in serum and urine, respectively (Figures 9A,B). This analysis aimed to provide a more comprehensive understanding of the interactions between gut microbiota and metabolites during the progression of IPF and to infer whether JBHD intervention modulates specific metabolites through certain microbial communities. The threshold for identifying reversed DAFMs and DEMs with statistical significance was also |r| > 0.5 and *p* < 0.05.

A total of 16 and 24 significant microbiota-metabolite correlations were determined between the reversed 4 DAFMs and the reversed 11 and 13 DEMs in serum (Figure 9A) and urine (Figure 9B) by JBHD, respectively. As shown in Figure 9A, *Lactobacillus* was positively associated with citrulline, stearic acid, and 7-ketodeoxycholic acid, but negatively linked to L-norleucine, muricholic acid and platelet-activating factor. Christensenellaceae R-7 group displayed an inverse correlation with stearic acid. *Clostridium* sensu stricto 1 correlated positively with L-norleucine, muricholic acid, LysoPC [22:5(7Z,10Z,13Z,16Z,19Z)/0:0], and platelet-activating factor, yet showed a negative relationship with 7-ketodeoxycholic acid. *Turicibacter* was positively related to L-norleucine and muricholic acid, while negatively correlated with citrulline and stearic acid.

In Figure 9B as well, *Lactobacillus* demonstrated positive correlations with L-tyrosine, uric acid, pyridoxal, and gentisic acid, but negative associations with 9-methyluric acid, homovanillic acid sulfate, 5-hydroxyindoleacetic acid, N-acetylneuraminic acid, and N-acetylgalactosamine 4-sulfate. Christensenellaceae R-7 group was positively associated with 9-methyluric acid, homovanillic acid sulfate, and 5-hydroxyindoleacetic acid, but negatively associated with L-tyrosine. *Clostridium* sensu stricto 1 showed positive correlations with homovanillic acid sulfate, 5-hydroxyindoleacetic acid, 3’-AMP, N-acetylneuraminic acid, and N-acetylgalactosamine 4-sulfate, while was negatively correlated with uric acid and gentisic acid. *Turicibacter* was positively linked to 9-methyluric acid and 5-hydroxyindoleacetic acid, but negatively associated with L-tyrosine and uric acid. These correlations suggest a complex interaction between the gut microbiome and metabolites, with implications for modulating microbial communities to promote health.

To investigate the interplay between the gut microbiota and host metabolism, we further constructed correlation networks between reversed DAFMs and reversed DEMs in serum and urine, respectively. The networks were generated based on Spearman correlations, and only associations with *p* < 0.05 were retained for visualization (Figures 9C,D). Topological analyses were further performed to characterize network properties and identify hub nodes, with the detailed centrality measures summarized in Supplementary Tables 6, 7.

In the reversed DAFMs—serum reversed DEMs network (Figure 9C and Supplementary Table 6), *Lactobacillus* exhibited the highest degree centrality (0.6154) and closeness centrality (0.6500), together with a relatively high betweenness centrality (0.2248), suggesting its pivotal role within the network. *Clostridium* sensu stricto 1 and *Turicibacter* also showed relatively high degree centrality values (0.5385 each). Notably, *Clostridium* sensu stricto 1 displayed the highest betweenness centrality (0.2536), indicating its potential bridging role. Among reversed DEMs, muricholic acid ranked highest for degree (0.3077), closeness (0.5909), and betweenness centrality (0.1004), indicating its broad connectivity with reversed DAFMs and its role as a central node linking distinct clusters.

In the reversed DAFMs—urine reversed DEMs network (Figure 9D and Supplementary Table 7), *Lactobacillus* exhibited the highest degree centrality (0.75), closeness centrality (0.7619), and betweenness centrality (0.3165), confirming its role as the dominant hub genus. *Clostridium* sensu stricto 1 and *Turicibacter* also displayed relatively high degree values (0.6875 each) and betweenness centrality (0.1947 and 0.1802, respectively), whereas Christensenellaceae R-7 group showed lower centrality measures. Among reversed DEMs, uric acid, pseudouridine, L-tyrosine, homovanillic acid sulfate, and 5-Hydroxyindoleacetic acid exhibited identical centrality measures, all of which were higher than those of the other reversed DEMs. Taken together, these findings indicate that *Lactobacillus* acts as a universal hub genus across both networks, underscoring its pivotal role in shaping host microbiota – metabolite interactions.

## 4 Discussion

IPF is a progressive interstitial lung disease marked by irreversible alveolar damage and excessive extracellular matrix accumulation. Increasing evidence suggests that both gut microbiota dysbiosis and host metabolic disturbances are implicated in its pathogenesis. Our ongoing work, presented in another manuscript currently under peer review, has demonstrated that JBHD exerts therapeutic effects against IPF, with no significant difference in efficacy compared with PFD. In the present study, we further elucidated the underlying mechanisms of JBHD, focusing on its modulatory effects along the gut–lung axis.

### 4.1 JBHD may modulate gut microbiota to influence gut-lung axis immune homeostasis

The lung and gut microbiota are functionally linked via the gut-lung axis, a bidirectional communication system mediated by blood and lymphatic circulation (Dong et al., 2024). Recent evidence suggests that dysbiosis of the gut microbiome may contribute to pulmonary injury (Cai et al., 2023a). Bleomycin-induced pulmonary fibrosis disrupts intestinal barrier integrity, resulting in gut microbiota dysbiosis and triggering immune-inflammatory responses (Quan et al., 2022). Growing evidence have further indicated that alterations in the composition and metabolic function of intestinal microbiota are closely associated with the initiation and progression of IPF (Sun et al., 2024). Therefore, modulation of gut microbial abundance and diversity may offer a promising therapeutic strategy for IPF.

In this study, we identified several DAFMs that were significantly modulated following JBHD treatment. Among these, *Lactobacillus*, sharply reduced in the Model, was restored in the JBHD groups (Figure 3); LEfSe confirmed its significant enrichment after JBHD (Figures 4B,C), suggesting it may participate in host–microbiota modulation. Previous studies have also reported that bleomycin-induced IPF decreases the relative abundance of *Lactobacillus*, which may contribute to the exacerbation of fibrosis, suggesting that probiotic *Lactobacillus* could exert beneficial effects in mitigating pulmonary fibrosis (Quan et al., 2022; Hu et al., 2023; Song et al., 2024). Functionally, *Lactobacillus* is well recognized for its ability to modulate gut and mucosal immunity, promote anti-inflammatory responses, enhance barrier integrity, and maintain microbial homeostasis (Heczko et al., 2024).

*Turicibacter*, another DAFM that was enriched in the Model, was reversed following JBHD intervention (Figure 3). This genus is closely associated with host immune regulation and has shown strong negative correlations with antioxidant enzyme activities and pro-inflammatory cytokines in immunosuppressed mice, suggesting its involvement in immune dysregulation (Tang et al., 2022). Additionally, clinical studies reveal that higher abundances of *Turicibacter* are linked to both improved responses to immune checkpoint inhibitor therapy and increased risk of immune-related adverse events in cancer patients, suggesting its dual role in enhancing immune activation and modulating immunotherapy outcomes (Hamada et al., 2023). Recent evidence suggests that *Turicibacter* is involved in the gut–lung axis and may act as a risk factor in lung diseases such as the acute exacerbation of obstructive pulmonary disease, and the significant differential abundance observed in our study highlights its potential relevance in the pathogenesis and modulation of pulmonary conditions (Li et al., 2021).

Other DAFMs reversed by JBHD included *Clostridium* sensu stricto 1, which has been reported to significantly correlate with the inflammatory marker C-reactive protein, indicating its potential role in the inflammatory phase of pulmonary fibrosis and its involvement in gut–lung axis interactions contributing to disease progression (Hou et al., 2024). In addition, Christensenellaceae R-7 group, which was also enriched in the Model and significantly reduced following JBHD-H treatment (Figures 3D,F), has been associated with shifts in key inflammatory and immunoregulatory cytokines, such as TNF-α, IL-1β, TGF-β, and IL-10, as well as changes in short-chain fatty acid profiles, indicating its potential role in modulating the intestinal immune microenvironment (Li et al., 2022); moreover, its differential abundance has been observed in lung cancer patients, suggesting a possible involvement in gut–lung axis interactions and respiratory disease progression (Chen et al., 2023).

In addition to changes in microbial composition, JBHD also modulated microbial functions. KEGG-based analysis indicated that both JBHD-L and JBHD-H affected amino acid metabolism and quorum sensing, pathways that are fundamental for microbial activity and have well-recognized links to host immune regulation. Amino acid metabolism plays a pivotal role in shaping immune cell functions (Yang et al., 2023), while quorum sensing signals, such as 2’-aminoacetophenone, have been shown to modulate host immune metabolism by reprogramming cellular bioenergetics (Chakraborty et al., 2024). Notably, JBHD-H appeared to affect additional pathways, including purine metabolism, which is increasingly recognized as contributors to immune homeostasis (Yang et al., 2025). COG analysis further corroborated functional findings, showing that JBHD-H was enriched in immune-relevant pathways. Notably, the involvement of amino acid metabolism in both KEGG and COG (E) underscores its central role in orchestrating immune cell functions. Additionally, COG category M has also been implicated in immune regulation (Yin et al., 2023).

Taken together, these findings suggest that JBHD intervention may influence key microbial taxa and functions along the gut-lung axis to improve immune homeostasis in IPF. Notably, JBHD-H showed a broader range of species and function level reverse than JBHD-L, implying that the observed differences may be attributed to dosage, with higher doses exerting stronger regulatory effects.

### 4.2 JBHD may influence host metabolic homeostasis through modulation of amino acid and lipid metabolism

**FIGURE 10:**
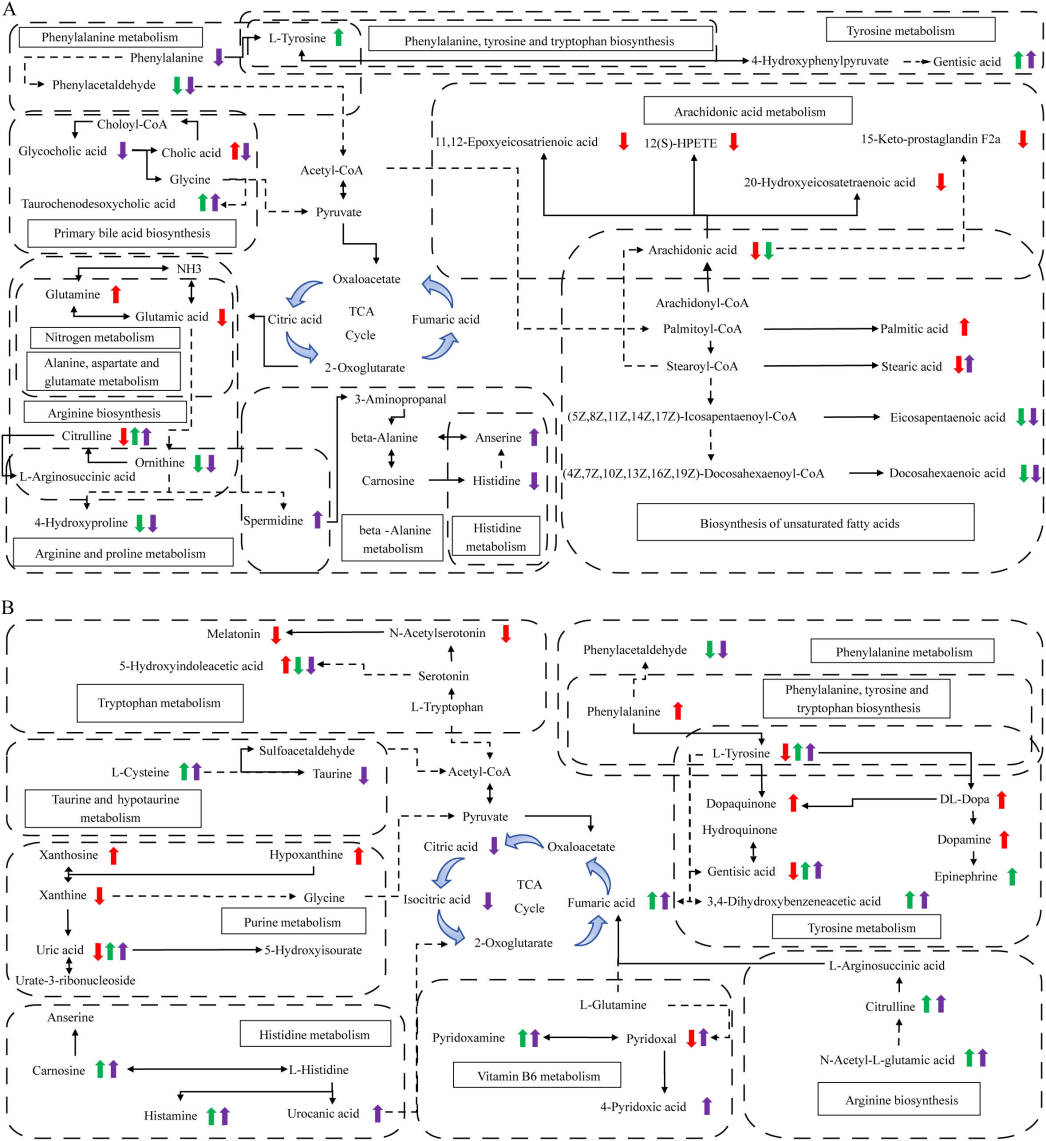
Diagram of the main potential metabolic pathways network in serum **(A)** and urine **(B)**. Metabolites changes are indicated by colored arrows representing relative abundance shifts among experimental groups: Red, green, and purple arrows denote metabolites in Model vs. Control, JBHD-L vs. Model, and JBHD-H vs. Model pairs, respectively. JBHD-L and JBHD-H, JiaweiBuyangHuanwu Decoction at low (10 g/kg) and high (20 g/kg) doses, respectively.

Metabolomics technology is an effective method of exploring the overall metabolic changes that occur in an organism under a specific condition. In this study, multi-metabolomic analyses of serum and urine were conducted, the related pathways and DEMs were used to construct the metabolic pathway network (Figure 10). Through this network, JBHD was found to be associated with changes in multiple metabolic pathways. These alterations suggest that JBHD may have a broad and multi-faceted influence on systemic metabolism in IPF. Notably, amino acid metabolism and lipid metabolism were identified as key pathways potentially mediating the therapeutic effects of JBHD.

Liu F. et al. (2024) reported that regulation of amino acid metabolism can alleviate the progression of pulmonary fibrosis. In this study, citrulline was identified as a reversed DEM following JBHD intervention and was involved in the Arginine biosynthesis. Notably, citrulline has been shown to improve certain pulmonary diseases, such as asthma, acute respiratory distress syndrome, and post-operative pulmonary hypertension, by enhancing nitric oxide production, which promotes alveolar development and reduces inflammation (Summar, 2024). In addition, citrulline may be efficacious in decreasing early lung inflammation and oxidative stress mitigating progression to bronchopulmonary dysplasia (Ivanovski et al., 2023). Moreover, co-supplementation of citrulline and L-glutamine has been demonstrated to alleviate pulmonary fibrosis and central nervous system impairment in mice (Ming et al., 2023). Spermidine, a key metabolite derived from the arginine and proline metabolic pathway, exerts antioxidant and anti-inflammatory effects and regulates physiological processes such as cell proliferation, differentiation, and apoptosis, with exogenous supplementation shown to protect against pulmonary fibrosis (Liu J. Q. et al., 2024). In this study, JBHD-H treatment resulted in a significant elevation of spermidine levels compared to the Model group, and its levels were partly regulated by gut microbiota (Yu et al., 2024), suggesting JBHD may indirectly influence its production through microbiota modulation.

Recent studies have shown that dysregulated lipid metabolism, including alterations in bioactive lipids, plays a crucial role in the initiation and progression of pulmonary fibrosis (Chen and Dai, 2023). Specifically, disturbances in Primary bile acid biosynthesis, including repeated microaspiration of major bile acids such as chenodeoxycholic acid, deoxycholic acid, and lithocholic acid, may exacerbate fibrotic processes by stimulating fibrogenic mediators, activating TGF-β1/Smad3 signaling, and enhancing farnesoid X receptor expression in lung tissue (Chen et al., 2017). Stearic acid, a DEM reversed by JBHD and classified under the Biosynthesis of unsaturated fatty acids, has been demonstrated to possess anti-inflammatory, antioxidant, and antifibrotic effects by inhibiting fibroblast activation, epithelial–mesenchymal transition, TGF-β1-induced Smad2/3 signaling, and reactive oxygen species generation (Kim et al., 2021). In addition, platelet-activating factor, another DEM reversed by JBHD, is a potent phospholipid-derived mediator that promotes immune cell activation, vascular permeability, and inflammatory signaling cascades (Upton et al., 2022). Notably, increased expression of its receptor has been observed in the small airway epithelium, type 2 pneumocytes, and alveolar macrophages in the lungs of patients with IPF (Shahzad et al., 2024), suggesting a role in aggravating the inflammatory microenvironment.

Notably, the metabolic regulation differed between the JBHD-L and JBHD-H treatments. Compared with JBHD-L, JBHD-H was associated with more alterations in metabolites and pathways. This dose-related divergence is also consistent with the microbiome results, in which JBHD-H displayed broader regulatory effects compared than JBHD-L. Such parallel patterns between metabolomic and microbial modulation could support the view that JBHD may exert its anti-fibrotic benefits through a systemic, dose-responsive rebalancing of host homeostasis.

### 4.3 JBHD may reconstruct host homeostasis via gut microbiota-associated systemic metabolic modulation

To investigate the mechanistic basis of JBHD’s influence on the gut–lung axis via gut microbiota–host interactions, a comprehensive microbiota–metabolite co-network was established using Spearman correlation combined with network topology analysis. The results highlighted that JBHD-induced shifts in intestinal microbial composition were closely associated with changes in host metabolic profiles, suggesting that JBHD may contribute to gut microbiota–centered remodeling of immunometabolic balance.

*Lactobacillus* emerged as a dominant hub genus within the co-network, exhibiting the highest degree and centrality metrics. It showed strong positive correlations with anti-inflammatory metabolites such as citrulline and stearic acid. Notably, *Lactobacillus murinus*, a species within this genus, can promote the synthesis of L-citrulline, which in turn contributes to the attenuation of inflammation (Long et al., 2025). The observed positive correlation between *Turicibacter* and muricholic acid may result from the capacity of *Turicibacter* strains to alter host bile acid composition and lipid metabolism (Lynch et al., 2023). *Clostridium* sensu stricto 1 served as a “bridge node” in the co-network, being positively associated with platelet-activating factor, and was implicated in immune cell activation and inflammatory signaling. Its topological position may reflect an association with host immune and inflammatory responses, possibly mediated by microbiota–metabolite interactions.

Further supporting these microbial influences on host metabolism, KEGG enrichment of the reversed DEMs (Figure 8) indicated involvement of multiple immune-related signaling pathways, including TNF, PI3K-Akt, and cAMP signaling, among others. Importantly, most of these metabolites with putative immunomodulatory functions were also found to be significantly correlated with specific microbial taxa in the JBHD group (Figure 9), particularly *Lactobacillus*, *Turicibacter*, and *Clostridium* sensu stricto 1, supporting the possibility that gut microbial alterations are associated with changes in host immunometabolism.

To evaluate the functional implications of microbiota remodeling, both KEGG Level 3 and COG analyses indicated notable enrichment in amino acid related pathways, including Biosynthesis of amino acids (KEGG) and Amino acid transport and metabolism (COG). Consistently, metabolomic KEGG annotation of the reversed DEMs also revealed significant enrichment in amino acid metabolism. These multi-omics findings collectively suggest that JBHD may be linked to modulation of amino acid metabolism via gut microbiota–host interactions, potentially contributing to the reestablishment of immunometabolic homeostasis.

Collectively, these findings suggest that JBHD may be involved in modulating immune responses not merely through isolated alterations in gut microbiota or host metabolites. Instead, it may act through coordination of a microbiota-associated metabolic network, which might influence systemic immune signaling and contribute to maintaining gut–lung axis homeostasis.

## 5 Conclusion

In conclusion, this study provides preliminary insights into a potential mechanism through which JBHD may influence the progression of IPF by modulating gut microbiota and host metabolic profiles. JBHD intervention partially reversed IPF-induced gut dysbiosis. Amino acid metabolism was identified as a commonly enriched function in JBHD-regulated microbiota, as revealed by both KEGG Level 3 and COG analyses. Consistently, untargeted metabolomic analysis of serum and urine showed alterations in amino acid metabolism, along with additional modifications in lipid metabolism. The observed correlations between DAFMs and DEMs suggest that microbial changes may be associated with systemic metabolic regulation. Functional annotation of reversed metabolites pointed toward possible involvement in immune-related signaling, supporting the view that JBHD may play a role in regulating immune balance via the gut–lung axis. Overall, this work provides preliminary correlations linking microbiota changes to host metabolic and immune responses and lays the groundwork for further investigation of JBHD as a potential multi-target strategy in IPF research. However, this study has certain limitations. Future investigations will employ metagenomic sequencing to validate microbial functional predictions and fecal microbiota transplantation to elucidate the causal roles of key taxa and metabolites in host immune–metabolic regulation. Moreover, large-scale studies applying rigorous statistical approaches are required to confirm the robustness and reproducibility of the metabolomic findings. In parallel, further studies are needed to verify the safety of JBHD in the treatment of IPF.

## Data Availability

The datasets presented in this study can be found in online repositories. The names of the repository/repositories and accession number(s) can be found at: https://www.ncbi.nlm.nih.gov/, PRJNA1273883.

## Footnotes

1
https://cloud.majorbio.com

2
http://www.hmdb.ca/

3
https://www.mzcloud.org

4
http://www.metaboanalyst.ca/

5
https://pubchem.ncbi.nlm.nih.gov/

6
http://www.swisstargetprediction.ch/

7
https://metascape.org/gp/index.html#/main/step1
