# Supplementary material for: Jiawei Buyang Huanwu Decoction modulates gut microbiota and metabolic profiles in a rat model of idiopathic pulmonary fibrosis
Source: Front Microbiol. 2025 Dec 2;16:1704103. doi: 10.3389/fmicb.2025.1704103 (PMC12707051; doi:10.3389/fmicb.2025.1704103)
Supplement: Supplementary file 1 [file Data_Sheet_1.docx]

**Details of Authentic Standards**

Hydroxysafflor yellow A (Lot No. 111637-202111), ferulic acid (Lot No. 110773-201915), hyperoside (Lot No. 111521-201406), astragaloside IV (Lot No. 110781-202219), paeoniflorin (Lot No. 110736-202145), baohuoside I (Lot No. 111852-201603), amygdalin (Lot No. 110820-201808), icariin (Lot No. 110737-202017), gallic acid (Lot No. 110831-201906) were purchased from National Institutes for Food and Drug Control (Beijing, China). Ligustilide (Lot No. A23GB158724), formononetin (Lot No. H06S9Z69494), adenine (Lot No. D09S10S97125), chlorogenic acid (Lot No. H06S9Z69494), Levistilide A (Lot No. J04HB184105), curculigoside (Lot No. C27J9G66557), albiflorin (Lot No. O31GB166251), oxypaeoniflorin (Lot No. N26GB169459), epimedin B (Lot No. G19A11L121806), epimedin A (Lot No. J12HB184811), cryptochlorogenic acid (Lot No. J01GB147635), astragaloside II (Lot No. J07HB173633), neochlorogenic acid (Lot No. D23GB172337), calycosin (Lot No. A03GB156838), astragaloside I (Lot No. Y23N10H104072), ononin (Lot No. O18GB164369), calycosin 7-O-glucoside (Lot No. G19A11L121806), galloylpaeoniflorin (Lot No. J01HB186722), senkyunolide H (Lot No. S25GB162334), hypoxanthine (Lot No. T13J11X107944), guanosine (Lot No. J09GB154303) were purchased from Shanghai yuanye Bio-Technology Co., Ltd. (Shanghai, China).

**Sample Preparation for Chemical Composition Analysis of JBHD**

JBHD (5.55 g) was soaked in 111 mL distilled water for 30 min, and then extracted by reflux extraction for 1 h. After cooling to room temperature, the extract was centrifuged at 4000 rpm for 15 min. The resulting supernatant was concentrated to 33.3 mL. An aliquot of 3 mL concentrate was transferred to a 10.0 mL volumetric flask and diluted to volume with methanol. The solution was sonicated for 20 min, cooled to room temperature, and adjusted to the mark if necessary. Finally, the sample was filtered through a 0.22-μm membrane filter before analysis by UPLC-Q-Exactive Orbitrap MS (Thermo Fisher Scientific, San Jose, CA, USA)

**UPLC-Q-Exactive Orbitrap MS conditions for the chemical components analysis of JBHD**

Chromatographic separation was performed using a Thermo Acclaim™ RSLC 120 C18 (2.1 mm×100 mm, 2.2 μm). The mobile phase consisted of acetonitrile (A phase) and 0.1% formic acid solution (B phase). The gradient elution procedure was as follows: 95% B (0–3 min), 95%–78% B (3–5 min), 78% B (5–13 min), 78%–50% B (13–14 min), 50%–46% B (14–15 min), 46% B (15–22 min), 46% B–10% B (22–32 min), 10% B (32–33 min), 10%–5% B (33–34 min), 5% B (34–35 min). The flow rate was set at 0.3 mL/min, the injection volume was 1 μL,and the column temperature was 35 ℃. Mass spectrometric detection was carried out in both positive and negative modes using a H-ESI source. Sheath gas and aux gas were set 40 and 10 arb, respectively. The spray voltage was set at 3.0 kV for positive mode and 2.8 kV for negative mode. Additional parameters included a capillary temperature of 325 ℃, S-lens RF level of 60, probe heater temperature of 350 ℃, and a scan range of m/z 80–1200. Data acquisition was performed at collision energies of 20, 40, and 60 eV.

Supplementary Table 1 Identified of chemical composition of JBHD

| No. | Compound name | Formula | Adduct ion | Observed m/z | ppm | Fragment ions m/z |
| --- | --- | --- | --- | --- | --- | --- |
| 1 | Adenine | C_5_H_5_N_5_ | [M+H]⁺ | 136.06181 | 0.28 | 119.03518, 94.04028 |
| 2 | Hypoxanthine | C_5_H_4_N_4_O | [M+H]⁺ | 137.04584 | 0.38 | 119.03514, 110.03490, 94.04063 |
| 3 | Guanosine | C_10_H_13_N_5_O_5_ | [M+H]⁺ | 284.09897 | 0.09 | 152.05646, 135.03020, 110.03490 |
| 4 | Gallic acid | C_7_H_6_O_5_ | [M−H]⁻ | 169.01317 | 0.12 | 125.02306, 122.89243, 107.01242, 97.02810, 81.03311, 69.03306 |
| 5 | Neochlorogenic acid | C_16_H_18_O_9_ | [M−H]⁻ | 353.08780 | -0.02 | 191.05508, 179.03391, 161.02309, 135.04381, 85.02811 |
| 6 | Oxypaeoniflorin | C_23_H_28_O_12_ | [M−H]⁻ | 495.15091 | 0.22 | 137.02316, 93.03301 |
| 7 | Chlorogenic acid | C_16_H_18_O_9_ | [M−H]⁻ | 353.08762 | -0.52 | 191.05515, 179.03391, 135.04739, 85.02811 |
| 8 | hydroxysafflor yellow A | C_27_H_32_O_16_ | [M−H]⁻ | 611.16144 | -0.52 | 119.04894, 491.11945, 325.07144, 403.10291, 109.02807, 163.00235, 283.06137, 85.02805, 473.10791, 593.15009 |
| 9 | Cryptochlorogenic acid | C_16_H_18_O_9_ | [M−H]⁻ | 353.08762 | -0.52 | 191.05513, 85.02807, 179.03349 |
| 10 | Amygdalin | C20H27NO11 | [M+HCOO]⁻ | 502.15689 | 0.55 | 323.09839, 89.02309, 101.02319 |
| 11 | Albiflorin | C_23_H_28_O_11_ | [M+H]⁺ | 481.16995 | -1.01 | 197.08087, 133.06477, 121.06506, 105.03380 |
| 12 | Paeoniflorin | C_23_H_28_O_11_ | [M+HCOO]⁻ | 525.16064 | -1.38 | 449.14395, 121.02814 |
| 13 | Calycosin 7-O-glucoside | C_22_H_22_O_10_ | [M+H]⁺ | 447.12817 | -0.90 | 285.07526, 270.05167, 137.02301 |
| 14 | [Hyperoside](https://www.chembk.com/en/chem/2-(3,4-dihydroxyphenyl)-3-(%CE%B2-D-glucofuranosyloxy)-5,7-dihydroxy-4H-1-benzopyran-4-one" \o "https://www.chembk.com/en/chem/2-(3,4-dihydroxyphenyl)-3-(%CE%B2-D-glucofuranosyloxy)-5,7-dihydroxy-4H-1-benzopyran-4-one) | C_21_H_20_O_12_ | [M+H]⁺ | 465.10266 | -0.20 | 303.04941, 153.01775, 229.04919, 257.04489, 285.03860, 201.05450 |
| 15 | [Galloylpaeoniflorin](https://www.chembk.com/en/chem/Galloylpaeoniflorin) | C_30_H_32_O_15_ | [M−H]⁻ | 631.16711 | 0.42 | 169.01311, 125.02309, 107.01259, 151.00237, 211.02357, 313.05646, 271.04572 |
| 16 | [Ferulic acid](https://www.chembk.com/en/chem/4-Hydroxy-3-methoxycinnamic%20acid) | C_10_H_10_O_4_ | [M+H]⁺ | 195.06502 | -0.85 | 177.05441, 145.02823, 117.03359, 149.05960, 89.03899 |
| 17 | Curculigoside | C22H26O11 | [M−H]⁻ | 465.14011 | -0.27 | 283.08221, 181.04962, 163.03894, 145.02863, 137.05956, 121.02814，93.03320 |
| 18 | Ononin | C_22_H_22_O_9_ | [M+H]⁺ | 431.13327 | -0.90 | 269.08023, 254.05695, 213.09068, 118.04135, 154.02541 |
| 19 | [Senkyunolide H](https://www.chembk.com/en/chem/SenkyunolideH) | C_12_H_16_O_4_ | [M+Na]⁺ | 247.09393 | -0.61 | 131.87175, 71.31339 |
| 20 | [Calycosin](https://www.chembk.com/en/chem/7-hydroxy-3-(3-hydroxy-4-methoxyphenyl)-4H-chromen-4-one" \o "https://www.chembk.com/en/chem/7-hydroxy-3-(3-hydroxy-4-methoxyphenyl)-4H-chromen-4-one) | C_16_H_12_O_5_ | [M+H]⁺ | 285.07578 | 0.11 | 270.05188, 253.04881, 225.05411, 197.05948, 137.02309 |
| 21 | Epimedin A | C39H50O20 | [M−H]⁻ | 837.28326 | 1.19 | 675.22906, 366.11005, 351.08588, 323.09250 |
| 22 | Epimedin B | C_38_H_48_O_19_ | [M+H]⁺ | 809.28534 | -1.13 | 531.18549, 369.13232, 313.06982, 85.02883 |
| 23 | [Icariin](https://www.chembk.com/en/chem/Icariin) | C_33_H_40_O_15_ | [M+H]⁺ | 677.24316 | -1.24 | 531.18475, 369.13220, 313.06973, 85.02879 |
| 24 | Astragaloside IV | C41H68O14 | [M+HCOO]⁻ | 829.45996 | 1.03 | 783.45648, 179.05432, 119.03336, 89.02287, 71.01244 |
| 25 | Astragaloside II | C_43_H_70_O_15_ | [M+HCOO]⁻ | 871.46979 | 0.13 | 825.46820, 89.02311 |
| 26 | Formononetin | C_16_H_12_O_4_ | [M+H]⁺ | 269.08032 | -1.92 | 213.09032, 237.05396, 253.04880, 107.04922, 118.04128, 137.02327, 154.02586, 181.06468, 197.05960 |
| 27 | [Baohuoside I](https://www.chembk.com/en/chem/baohuoside%20I) | C_27_H_30_O_10_ | [M−H]⁻ | 513.17670 | 0.16 | 366.11057, 351.08710, 323.09219, 279.02939, 217.04984 |
| 28 | Astragaloside I | C_45_H_72_O_16_ | [M+Na]⁺ | 891.46979 | -1.65 | 711.40387, 203.05667 |
| 29 | [Ligustilide](https://www.chembk.com/en/chem/3-butylphthalide) | C_12_H_14_O_2_ | [M+H]⁺ | 191.10648 | -0.92 | 173.09593, 163.11172, 149.05963, 145.10085, 117.06994, 105.07011, 91.05467 |
| 30 | Levistilide A | C_24_H_28_O_4_ | [M+H]⁺ | 381.20578 | -0.67 | 191.10637, 135.04410, 149.05974, 173.09593, 91.05466, 79.05461 |

Notes: JBHD, Jiawei Buyang Huanwu Decoction

Supplementary Table 2 The identified DEMs of serum samples

| No. | Compound name | HMDB ID | Adduct ion | RT/min | Observed m/z | Mass error (ppm) | Formula | Log_2_(FC) | P | VIP | Metabolite Superclass |
| --- | --- | --- | --- | --- | --- | --- | --- | --- | --- | --- | --- |
|  | A：Model *vs.* Control |  |  |  |  |  |  |  |  |  |  |
| 1 | L-Acetylcarnitine | HMDB0000201 | [M+H]⁺ | 0.90 | 204.12296 | -0.37 | C_9_H_17_NO_4_ | 1.03 | 1.38E-02 | 5.72 | Lipids and lipid-like molecules |
| 2 | 15-Keto-prostaglandin F2a | HMDB0004240 | [M+H]⁺ | 6.11 | 353.23224 | -0.03 | C_20_H_32_O_5_ | -1.39 | 9.73E-04 | 1.38 | Lipids and lipid-like molecules |
| 3 | 8-HETE | HMDB0004679 | [M−H]⁻ | 15.39 | 319.22751 | 2.31 | C_20_H_32_O_3_ | -1.47 | 4.41E-02 | 1.74 | Lipids and lipid-like molecules |
| 4 | LysoPC(22:5(7Z,10Z,13Z,16Z,19Z)/0:0) | HMDB0010403 | [M+H]⁺ | 13.35 | 570.35559 | 0.31 | C_30_H_52_NO_7_P | 1.33 | 2.64E-02 | 1.08 | Lipids and lipid-like molecules |
| 5 | Platelet-activating factor | HMDB0062195 | [M+H]⁺ | 20.20 | 524.37109 | 0.05 | C_26_H_54_NO_7_P | 1.28 | 2.26E-04 | 6.91 | Lipids and lipid-like molecules |
| 6 | Propionylcarnitine | HMDB0000824 | [M+H]⁺ | 1.75 | 218.13875 | 0.30 | C_10_H_19_NO_4_ | 1.25 | 2.90E-02 | 1.2 | Lipids and lipid-like molecules |
| 7 | Sphinganine 1-phosphate | HMDB0001383 | [M+H]⁺ | 10.88 | 382.27176 | 0.19 | C_18_H_40_NO_5_P | -1.74 | 1.10E-03 | 3.31 | Lipids and lipid-like molecules |
| 8 | 11(R)-HETE | HMDB0004682 | [M−H]⁻ | 15.81 | 319.22760 | 2.60 | C_20_H_32_O_3_ | -1.47 | 2.78E-02 | 2.22 | Lipids and lipid-like molecules |
| 9 | Cervonoyl ethanolamide | HMDB0013627 | [M+H]⁺ | 8.01 | 373.27335 | -1.00 | C_24_H_36_O_3_ | -1.15 | 6.04E-03 | 1.76 | Lipids and lipid-like molecules |
| 10 | 9-cis-Retinal | HMDB0006218 | [M+H]⁺ | 15.41 | 285.22129 | 0.83 | C_20_H_28_O | -1.18 | 8.46E-04 | 2.3 | Lipids and lipid-like molecules |
| 11 | Docosapentaenoic acid (22n-3) | HMDB0006528 | [M+H]⁺ | 20.08 | 331.26288 | -0.84 | C_22_H_34_O_2_ | -1.13 | 3.93E-03 | 1.32 | Lipids and lipid-like molecules |
| 12 | Palmitic acid | HMDB0000220 | [M−H]⁻ | 13.99 | 255.23224 | 1.50 | C_16_H_32_O_2_ | 1.15 | 1.27E-02 | 1.43 | Lipids and lipid-like molecules |
| 13 | Stearic acid | HMDB0000827 | [M−H]⁻ | 20.18 | 283.26392 | 2.69 | C_18_H_36_O_2_ | -2.06 | 4.62E-02 | 1.16 | Lipids and lipid-like molecules |
| 14 | 12(S)-HPETE | HMDB0004243 | [M−H]⁻ | 12.30 | 335.22284 | 3.44 | C_20_H_32_O_4_ | -1.17 | 4.95E-02 | 1.84 | Lipids and lipid-like molecules |
| 15 | Thromboxane B2 | HMDB0003252 | [M−H]⁻ | 6.10 | 369.22839 | 3.32 | C_20_H_34_O_6_ | -1.29 | 3.27E-02 | 2.68 | Lipids and lipid-like molecules |
| 16 | Muricholic acid | HMDB0000865 | [M−H]⁻ | 6.51 | 407.28018 | 2.40 | C_24_H_40_O_5_ | 1.04 | 3.17E-02 | 3.3 | Lipids and lipid-like molecules |
| 17 | Deoxycholic acid | HMDB0000626 | [M−H]⁻ | 8.43 | 391.28558 | 3.31 | C_24_H_40_O_4_ | 1.42 | 7.66E-03 | 1.05 | Lipids and lipid-like molecules |
| 18 | 11,12-Epoxyeicosatrienoic acid | HMDB0004673 | [M−H]⁻ | 15.81 | 319.22760 | 2.60 | C_20_H_32_O_3_ | -1.3 | 1.07E-03 | 23.33 | Lipids and lipid-like molecules |
| 19 | Nutriacholic acid | HMDB0000467 | [M−H]⁻ | 10.23 | 389.27005 | 3.63 | C_24_H_38_O_4_ | -1.37 | 3.85E-02 | 1.43 | Lipids and lipid-like molecules |
| 20 | Arachidonic acid | HMDB0001043 | [M−H]⁻ | 23.10 | 303.23279 | 3.08 | C_20_H_32_O_2_ | -1.35 | 1.04E-02 | 5.06 | Lipids and lipid-like molecules |
| 21 | 7-ketodeoxycholic acid | HMDB0000391 | [M−H]⁻ | 7.01 | 405.26505 | 3.70 | C_24_H_38_O_5_ | -1.92 | 1.92E-02 | 2.3 | Lipids and lipid-like molecules |
| 22 | Cholic acid | HMDB0000619 | [M−H]⁻ | 8.04 | 407.27991 | 1.74 | C_24_H_40_O_5_ | 1.17 | 1.54E-02 | 1.68 | Lipids and lipid-like molecules |
| 23 | 20-Hydroxyeicosatetraenoic acid | HMDB0005998 | [M−H]⁻ | 15.39 | 319.22751 | 2.31 | C_20_H_32_O_3_ | -1.54 | 8.10E-04 | 1.39 | Lipids and lipid-like molecules |
| 24 | Glutamine | HMDB0000641 | [M+H]⁺ | 0.82 | 147.07646 | 0.28 | C_5_H_10_N_2_O_3_ | 1.17 | 6.41E-04 | 1.24 | Organic acids and derivatives |
| 25 | L-Norleucine | HMDB0001645 | [M+H]⁺ | 1.62 | 132.10207 | 1.25 | C_6_H_13_NO_2_ | 1.12 | 3.36E-01 | 2.17 | Organic acids and derivatives |
| 26 | Symmetric dimethylarginine | HMDB0003334 | [M+H]⁺ | 0.85 | 203.15001 | -1.19 | C_8_H_18_N_4_O_2_ | 1.31 | 3.05E-03 | 1.61 | Organic acids and derivatives |
| 27 | Citrulline | HMDB0000904 | [M+H]⁺ | 0.83 | 176.10298 | 0.07 | C_6_H_13_N_3_O_3_ | -1.25 | 3.63E-04 | 1.59 | Organic acids and derivatives |
| 28 | Creatine | HMDB0000064 | [M+H]⁺ | 0.87 | 132.07680 | 0.36 | C_4_H_9_N_3_O_2_ | -1.3 | 2.39E-02 | 6.14 | Organic acids and derivatives |
| 29 | Taurine | HMDB0000251 | [M+H]⁺ | 0.85 | 126.02213 | 1.50 | C_2_H_7_NO_3_S | -1.38 | 2.04E-03 | 1.56 | Organic acids and derivatives |
| 30 | Glutaminylglutamic acid | HMDB0028796 | [M−H]⁻ | 0.89 | 274.10403 | 2.44 | C_10_H_17_N_3_O_6_ | 1.61 | 2.04E-03 | 1.5 | Organic acids and derivatives |
| 31 | Glutamic acid | HMDB0000148 | [M−H]⁻ | 0.84 | 146.04460 | -1.26 | C_5_H_9_NO_4_ | -1.67 | 3.99E-02 | 2.42 | Organic acids and derivatives |
| 32 | Glutamylglutamine | HMDB0028817 | [M−H]⁻ | 0.89 | 274.10403 | 2.44 | C_10_H_17_N_3_O_6_ | 1.61 | 2.29E-03 | 1.23 | Organic acids and derivatives |
| 33 | D-Tryptophan | HMDB0013609 | [M+H]⁺ | 4.54 | 205.09718 | 0.13 | C_11_H_12_N_2_O_2_ | -1.34 | 4.36E-02 | 5.97 | Organoheterocyclic compounds |
| 34 | Indoleacrylic acid | HMDB0000734 | [M+H]⁺ | 4.54 | 188.07076 | 0.82 | C_11_H_9_NO_2_ | -1.32 | 6.29E-04 | 1.03 | Organoheterocyclic compounds |
| 35 | Dihydrothymine | HMDB0000079 | [M+H]⁺ | 0.86 | 129.06593 | 0.59 | C_5_H_8_N_2_O_2_ | 1.55 | 4.81E-04 | 1.15 | Organoheterocyclic compounds |
| 36 | Uric acid | HMDB0000289 | [M−H]⁻ | 1.30 | 167.01997 | 0.02 | C_5_H_4_N_4_O_3_ | -1.35 | 4.69E-02 | 2.4 | Organoheterocyclic compounds |
| 37 | Ascorbic acid 2-sulfate | HMDB0060649 | [M−H]⁻ | 1.30 | 254.98123 | 2.75 | C_6_H_8_O_9_S | 1.39 | 2.71E-02 | 1.3 | Organoheterocyclic compounds |
| 38 | Indolelactic acid | HMDB0000671 | [M−H]⁻ | 5.20 | 204.06598 | 2.26 | C_11_H_11_NO_3_ | -1.82 | 4.90E-03 | 1.23 | Organoheterocyclic compounds |
| 39 | L-Carnitine | HMDB0000062 | [M+H]⁺ | 0.82 | 162.11243 | -0.25 | C_7_H_15_NO_3_ | 1.52 | 8.12E-03 | 10 | Organic nitrogen compounds |
| 40 | Sphingosine | HMDB0000252 | [M+H]⁺ | 8.97 | 300.28976 | 0.18 | C_18_H_37_NO_2_ | -1.32 | 1.77E-02 | 1.23 | Organic nitrogen compounds |
|  | B：JBHD-L *vs.* Model |  |  |  |  |  |  |  |  |  |  |
| 1 | Taurochenodesoxycholic acid | HMDB0000951 | [M−H]⁻ | 6.63 | 498.28967 | 2.58 | C_26_H_45_NO_6_S | 1.1 | 2.70E-02 | 2.53 | Lipids and lipid-like molecules |
| 2 | 3-Carboxy-4-methyl-5-propyl-2-furanpropionic acid | HMDB0061112 | [M−H−H_2_O]⁻ | 7.30 | 221.08131 | 2.15 | C_12_H_16_O_5_ | 4.85 | 7.19E-08 | 1.04 | Lipids and lipid-like molecules |
| 3 | 12-Hydroxystearic acid | HMDB0061706 | [M−H]⁻ | 19.47 | 299.25894 | 2.90 | C_18_H_36_O_3_ | -1.49 | 1.80E-02 | 1.18 | Lipids and lipid-like molecules |
| 4 | Arachidonic acid | HMDB0001043 | [M−H]⁻ | 23.10 | 303.23279 | 3.08 | C_20_H_32_O_2_ | -1.02 | 7.12E-03 | 3.39 | Lipids and lipid-like molecules |
| 5 | Docosahexaenoic Acid | HMDB0002183 | [M−H]⁻ | 22.95 | 327.23267 | 2.49 | C_22_H_32_O_2_ | -1.35 | 1.03E-03 | 5.03 | Lipids and lipid-like molecules |
| 6 | 9(S)-HPODE | HMDB0006940 | [M−H]⁻ | 13.14 | 311.22253 | 2.71 | C_18_H_32_O_4_ | -1.66 | 9.82E-04 | 2.89 | Lipids and lipid-like molecules |
| 7 | Muricholic acid | HMDB0000865 | [M−H]⁻ | 6.51 | 407.28018 | 2.40 | C_24_H_40_O_5_ | -1.07 | 1.55E-02 | 1.83 | Lipids and lipid-like molecules |
| 8 | Deoxycholic acid | HMDB0000626 | [M−H]⁻ | 8.43 | 391.28558 | 3.31 | C_24_H_40_O_4_ | -1.31 | 3.00E-02 | 1.88 | Lipids and lipid-like molecules |
| 9 | 16-Hydroxyhexadecanoic acid | HMDB0006294 | [M−H]⁻ | 22.29 | 271.22787 | 4.05 | C_16_H_32_O_3_ | -1.1 | 3.50E-04 | 1.76 | Lipids and lipid-like molecules |
| 10 | Docosapentaenoic acid (22n-3) | HMDB0006528 | [M+H]⁺ | 20.08 | 331.26288 | -0.84 | C_22_H_34_O_2_ | -1.17 | 3.83E-02 | 1.24 | Lipids and lipid-like molecules |
| 11 | Eicosapentaenoic acid | HMDB0001999 | [M−H]⁻ | 22.16 | 301.21716 | 3.16 | C_20_H_30_O_2_ | -1.25 | 5.05E-03 | 1.3 | Lipids and lipid-like molecules |
| 12 | Thromboxane B2 | HMDB0003252 | [M−H]⁻ | 6.10 | 369.22839 | 3.32 | C_20_H_34_O_6_ | 1.19 | 3.53E-03 | 1.89 | Lipids and lipid-like molecules |
| 13 | 7-ketodeoxycholic acid | HMDB0000391 | [M−H]⁻ | 7.01 | 405.26505 | 3.70 | C_24_H_38_O_5_ | 1.4 | 2.24E-03 | 1.27 | Lipids and lipid-like molecules |
| 14 | Nutriacholic acid | HMDB0000467 | [M−H]⁻ | 10.23 | 389.27005 | 3.63 | C_24_H_38_O_4_ | -1.43 | 2.99E-02 | 1.26 | Lipids and lipid-like molecules |
| 15 | Platelet-activating factor | HMDB0062195 | [M+H]⁺ | 20.20 | 524.37109 | 0.05 | C_26_H_54_NO_7_P | -1.3 | 8.98E-03 | 4.04 | Lipids and lipid-like molecules |
| 16 | LysoPC(22:5(7Z,10Z,13Z,16Z,19Z)/0:0) | HMDB0010403 | [M+H]⁺ | 13.35 | 570.35559 | 0.31 | C_30_H_52_NO_7_P | -1.31 | 3.42E-03 | 1.18 | Lipids and lipid-like molecules |
| 17 | Ornithine | HMDB0000214 | [M−H]⁻ | 0.85 | 131.08130 | -1.56 | C_5_H_12_N_2_O_2_ | -1.06 | 2.80E-05 | 1.94 | Organic acids and derivatives |
| 18 | D-Pipecolic acid | HMDB0005960 | [M+H]⁺ | 0.74 | 130.08640 | 1.11 | C_6_H_11_NO_2_ | 1.35 | 6.80E-04 | 3.66 | Organic acids and derivatives |
| 19 | Pipecolic acid | HMDB0000070 | [M+H]⁺ | 0.68 | 130.08646 | 1.57 | C_6_H_11_NO_2_ | 1.18 | 2.90E-06 | 3.37 | Organic acids and derivatives |
| 20 | N2,N5-Diacetylornithine | HMDB0240345 | [M+H]⁺ | 4.98 | 217.11737 | -0.30 | C_9_H_16_N_2_O_4_ | 2.05 | 2.46E-03 | 1.37 | Organic acids and derivatives |
| 21 | Leucylproline | HMDB0011175 | [M+H]⁺ | 0.90 | 229.15460 | -0.30 | C_11_H_20_N_2_O_3_ | 3.49 | 3.84E-06 | 1.15 | Organic acids and derivatives |
| 22 | Lysylglutamine | HMDB0028949 | [M+H]⁺ | 0.89 | 275.17120 | -0.66 | C_11_H_22_N_4_O_4_ | 1.24 | 1.24E-02 | 1.08 | Organic acids and derivatives |
| 23 | Citrulline | HMDB0000904 | [M+H]⁺ | 0.83 | 176.10298 | 0.07 | C_6_H_13_N_3_O_3_ | 1.12 | 4.51E-05 | 2.05 | Organic acids and derivatives |
| 24 | 4-Hydroxyproline | HMDB0000725 | [M+H]⁺ | 0.82 | 132.06561 | 0.68 | C_5_H_9_NO_3_ | -1.28 | 9.04E-05 | 1.3 | Organic acids and derivatives |
| 25 | L-Norleucine | HMDB0001645 | [M+H]⁺ | 1.62 | 132.10207 | 1.25 | C_6_H_13_NO_2_ | -1.28 | 2.44E-02 | 2.77 | Organic acids and derivatives |
| 26 | L-Tyrosine | HMDB0000158 | [M+H]⁺ | 1.46 | 182.08122 | 0.28 | C_9_H_11_NO_3_ | 1.54 | 2.51E-03 | 1.11 | Organic acids and derivatives |
| 27 | Phenylacetaldehyde | HMDB0006236 | [M−H]⁻ | 4.89 | 119.04890 | -2.03 | C_8_H_8_O | -1.21 | 5.18E-06 | 1.59 | Benzenoids |
| 28 | Gentisic acid | HMDB0000152 | [M−H]⁻ | 5.24 | 153.01828 | 0.29 | C_7_H_6_O_4_ | 4.12 | 7.51E-09 | 3.13 | Benzenoids |
| 29 | Hippuric acid | HMDB0000714 | [M+H]⁺ | 4.81 | 180.06567 | 1.28 | C_9_H_9_NO_3_ | 1.02 | 2.95E-02 | 1.94 | Benzenoids |
| 30 | Dihydrothymine | HMDB0000079 | [M+H]⁺ | 0.86 | 129.06593 | 0.59 | C_5_H_8_N_2_O_2_ | -1.43 | 2.12E-03 | 1.03 | Organoheterocyclic compounds |
| 31 | Cytosine | HMDB0000630 | [M+H]⁺ | 1.31 | 112.05091 | 3.32 | C_4_H_5_N_3_O | -1.03 | 3.93E-04 | 1.09 | Organoheterocyclic compounds |
| 32 | Indole | HMDB0000738 | [M+H]⁺ | 4.54 | 118.06547 | 2.91 | C_8_H_7_N | -1.2 | 2.97E-02 | 1.34 | Organoheterocyclic compounds |
|  | C：JBHD-H *vs.* Model |  |  |  |  |  |  |  |  |  |  |
| 1 | Taurochenodesoxycholic acid | HMDB0000951 | [M−H]⁻ | 6.63 | 498.28967 | 2.58 | C_26_H_45_NO_6_S | 1.57 | 1.05E-02 | 2.44 | Lipids and lipid-like molecules |
| 2 | 3a,6b,7b-Trihydroxy-5b-cholanoic acid | HMDB0000415 | [M−H]⁻ | 6.63 | 498.28967 | 2.58 | C_26_H_45_NO_6_S | 1.68 | 9.75E-04 | 18.09 | Lipids and lipid-like molecules |
| 3 | Glycocholic acid | HMDB0000138 | [M−H]⁻ | 5.38 | 464.30182 | 2.49 | C_26_H_43_NO_6_ | -1.43 | 5.60E-03 | 4.82 | Lipids and lipid-like molecules |
| 4 | Deoxycholic acid | HMDB0000626 | [M−H]⁻ | 8.43 | 391.28558 | 3.31 | C_24_H_40_O_4_ | -1.49 | 8.66E-03 | 1.15 | Lipids and lipid-like molecules |
| 5 | 16-Hydroxyhexadecanoic acid | HMDB0006294 | [M−H]⁻ | 22.29 | 271.22787 | 4.05 | C_16_H_32_O_3_ | -1.14 | 7.35E-03 | 1.44 | Lipids and lipid-like molecules |
| 6 | Stearic acid | HMDB0000827 | [M−H]⁻ | 20.18 | 283.26392 | 2.69 | C_18_H_36_O_2_ | 1.46 | 3.63E-04 | 1.47 | Lipids and lipid-like molecules |
| 7 | Eicosapentaenoic acid | HMDB0001999 | [M−H]⁻ | 22.16 | 301.21716 | 3.16 | C_20_H_30_O_2_ | -1.23 | 2.57E-03 | 1.05 | Lipids and lipid-like molecules |
| 8 | Cholic acid | HMDB0000619 | [M−H]⁻ | 8.04 | 407.27991 | 1.74 | C_24_H_40_O_5_ | -1.12 | 1.24E-02 | 14.82 | Lipids and lipid-like molecules |
| 9 | 7-ketodeoxycholic acid | HMDB0000391 | [M−H]⁻ | 7.01 | 405.26505 | 3.70 | C_24_H_38_O_5_ | 1.67 | 1.57E-02 | 1.53 | Lipids and lipid-like molecules |
| 10 | Muricholic acid | HMDB0000865 | [M−H]⁻ | 6.51 | 407.28018 | 2.40 | C_24_H_40_O_5_ | -1.47 | 1.33E-02 | 3.49 | Lipids and lipid-like molecules |
| 11 | LysoPC(20:2(11Z,14Z)/0:0) | HMDB0010392 | [M+H]⁺ | 16.47 | 548.37122 | 0.28 | C_28_H_54_NO_7_P | -1.15 | 6.62E-05 | 3.12 | Lipids and lipid-like molecules |
| 12 | Platelet-activating factor | HMDB0062195 | [M+H]⁺ | 20.20 | 524.37109 | 0.05 | C_26_H_54_NO_7_P | -1.73 | 4.21E-06 | 12.85 | Lipids and lipid-like molecules |
| 13 | Docosahexaenoic Acid | HMDB0002183 | [M−H]⁻ | 22.95 | 327.23267 | 2.49 | C_22_H_32_O_2_ | -1.02 | 7.56E-04 | 3.01 | Lipids and lipid-like molecules |
| 14 | 9(S)-HPODE | HMDB0006940 | [M−H]⁻ | 13.14 | 311.22253 | 2.71 | C_18_H_32_O_4_ | -1.01 | 2.28E-02 | 1.23 | Lipids and lipid-like molecules |
| 15 | Docosapentaenoic acid (22n-3) | HMDB0006528 | [M+H]⁺ | 20.08 | 331.26288 | -0.84 | C_22_H_34_O_2_ | -1.42 | 1.61E-02 | 1.23 | Lipids and lipid-like molecules |
| 16 | Ornithine | HMDB0000214 | [M−H]⁻ | 0.85 | 131.08130 | -1.56 | C_5_H_12_N_2_O_2_ | -1.4 | 1.12E-05 | 1.65 | Organic acids and derivatives |
| 17 | Histidine | HMDB0000177 | [M−H]⁻ | 0.79 | 154.06114 | 0.24 | C_6_H_9_N_3_O_2_ | -1.02 | 2.05E-04 | 1.01 | Organic acids and derivatives |
| 18 | Pipecolic acid | HMDB0000070 | [M+H]⁺ | 0.68 | 130.08646 | 1.57 | C_6_H_11_NO_2_ | 1.55 | 3.20E-05 | 3.21 | Organic acids and derivatives |
| 19 | Anserine | HMDB0000194 | [M+H]⁺ | 0.79 | 241.12920 | -1.31 | C_10_H_16_N_4_O_3_ | 1.4 | 4.20E-02 | 1.05 | Organic acids and derivatives |
| 20 | N2,N5-Diacetylornithine | HMDB0240345 | [M+H]⁺ | 4.98 | 217.11737 | -0.30 | C_9_H_16_N_2_O_4_ | 2.96 | 8.08E-08 | 1.67 | Organic acids and derivatives |
| 21 | gamma-Glutamylglycine | HMDB0011667 | [M+H]⁺ | 0.89 | 205.08116 | -3.60 | C_7_H_12_N_2_O_5_ | 6.61 | 4.23E-03 | 1.16 | Organic acids and derivatives |
| 22 | Leucylproline | HMDB0011175 | [M+H]⁺ | 0.90 | 229.15460 | -0.30 | C_11_H_20_N_2_O_3_ | 3.85 | 5.46E-06 | 1.02 | Organic acids and derivatives |
| 23 | Leucine | HMDB0000687 | [M+H]⁺ | 1.62 | 132.10191 | 1.25 | C_6_H_13_NO_2_ | -1.03 | 3.04E-04 | 8.19 | Organic acids and derivatives |
| 24 | L-Norleucine | HMDB0001645 | [M+H]⁺ | 1.62 | 132.10207 | 1.25 | C_6_H_13_NO_2_ | -1.31 | 1.82E-04 | 8.06 | Organic acids and derivatives |
| 25 | Phenylalanine | HMDB0000159 | [M+H]⁺ | 3.22 | 166.08627 | 0.09 | C_9_H_11_NO_2_ | -1.43 | 5.32E-07 | 10.52 | Organic acids and derivatives |
| 26 | Citrulline | HMDB0000904 | [M+H]⁺ | 0.83 | 176.10298 | 0.07 | C_6_H_13_N_3_O_3_ | 1.37 | 1.86E-05 | 1.65 | Organic acids and derivatives |
| 27 | 4-Hydroxyproline | HMDB0000725 | [M+H]⁺ | 0.82 | 132.06561 | 0.68 | C_5_H_9_NO_3_ | -2.07 | 5.67E-08 | 1.31 | Organic acids and derivatives |
| 28 | gamma-Glutamyltyrosine | HMDB0011741 | [M+H]⁺ | 4.38 | 311.12396 | 0.63 | C_14_H_18_N_2_O_6_ | -2.31 | 1.93E-05 | 1.14 | Organic acids and derivatives |
| 29 | Gentisic acid | HMDB0000152 | [M−H]⁻ | 5.24 | 153.01828 | 0.29 | C_7_H_6_O_4_ | 3.64 | 1.82E-04 | 3.49 | Benzenoids |
| 30 | Phenylacetaldehyde | HMDB0006236 | [M−H]⁻ | 4.89 | 119.04890 | -2.03 | C_8_H_8_O | -1.19 | 1.67E-05 | 1.52 | Benzenoids |
| 31 | spermidine | HMDB0001257 | [M+H]⁺ | 0.69 | 146.16537 | 1.34 | C_7_H_19_N_3_ | 1.51 | 1.60E-04 | 1.77 | Organic nitrogen compounds |
| 32 | Phytosphingosine | HMDB0004610 | [M+H]⁺ | 6.72 | 318.30032 | 0.16 | C_18_H_39_NO_3_ | -1.2 | 1.35E-03 | 1.66 | Organic nitrogen compounds |
| 33 | Glucosamine 6-phosphate | HMDB0001254 | [M+H]⁺ | 0.95 | 260.05310 | 0.46 | C_6_H_14_NO_8_P | 1.46 | 7.30E-03 | 1.05 | Organic oxygen compounds |
| 34 | 6-Methylnicotinamide | HMDB0013704 | [M+H]⁺ | 0.88 | 151.08701 | 2.78 | C_8_H_10_N_2_O | -1.13 | 8.31E-03 | 1.41 | Organoheterocyclic compounds |

Abbreviations: DEMs, differentially expressed metabolites; JBHD-L and JBHD-H, Jiawei Buyang Huanwu Decoction at low (10 g/kg) and high (20 g/kg) doses, respectively.

Supplementary Table 3 The identified DEMs of urine samples

| No. | Compound name | HMDB ID | Adduct ion | RT | Observed m/z | Mass error (ppm) | Formula | Log_2_(FC) | P | VIP | Metabolite Superclass |
| --- | --- | --- | --- | --- | --- | --- | --- | --- | --- | --- | --- |
|  | A：Model *vs.* Control |  |  |  |  |  |  |  |  |  |  |
| 1 | Homovanillic acid sulfate | HMDB0011719 | [M−H]⁻ | 5.07 | 261.00699 | -1.75 | C_9_H_10_O_7_S | 1 | 3.40E-03 | 8.65 | Organic acids and derivatives |
| 2 | Isovalerylglycine | HMDB0000678 | [M−H]⁻ | 5.59 | 158.08176 | -3.20 | C_7_H_13_NO_3_ | 1.05 | 3.53E-02 | 2.59 | Organic acids and derivatives |
| 3 | Threonyltyrosine | HMDB0029073 | [M−H]⁻ | 7.51 | 281.11435 | 0.20 | C_13_H_18_N_2_O_5_ | 1.2 | 1.81E-02 | 2 | Organic acids and derivatives |
| 4 | Nicotinuric acid | HMDB0003269 | [M+H]⁺ | 5.13 | 181.06041 | -1.98 | C_8_H_8_N_2_O_3_ | 1.39 | 1.60E-02 | 4.39 | Organic acids and derivatives |
| 5 | Creatinine | HMDB0000562 | [M+H]⁺ | 0.83 | 114.06631 | 1.07 | C_4_H_7_N_3_O | 1.11 | 3.79E-02 | 3.85 | Organic acids and derivatives |
| 6 | Phenylalanine | HMDB0000159 | [M+H]⁺ | 3.39 | 166.08601 | -1.48 | C_9_H_11_NO_2_ | 1.13 | 2.61E-03 | 3.16 | Organic acids and derivatives |
| 7 | N-a-Acetyl-L-arginine | HMDB0004620 | [M+H]⁺ | 0.91 | 217.12895 | -2.61 | C_8_H_16_N_4_O_3_ | 1.34 | 1.47E-02 | 2.05 | Organic acids and derivatives |
| 8 | L-Norleucine | HMDB0001645 | [M+H]⁺ | 0.88 | 132.10182 | -0.65 | C_6_H_13_NO_2_ | 1.08 | 2.29E-02 | 1.91 | Organic acids and derivatives |
| 9 | Valylproline | HMDB0029135 | [M+H]⁺ | 1.43 | 215.13841 | -2.83 | C_10_H_18_N_2_O_3_ | 1.47 | 2.81E-02 | 1.8 | Organic acids and derivatives |
| 10 | N5-Acetylornithine | HMDB0240589 | [M+H]⁺ | 2.08 | 175.10757 | -0.85 | C_7_H_14_N_2_O_3_ | 1.46 | 1.42E-02 | 1.46 | Organic acids and derivatives |
| 11 | L,L-Cyclo(leucylprolyl) | HMDB0034276 | [M+H]⁺ | 6.13 | 211.14375 | -1.68 | C_11_H_18_N_2_O_2_ | 1.67 | 4.64E-02 | 1.18 | Organic acids and derivatives |
| 12 | Prolyl-Glutamate | HMDB0029016 | [M+H]⁺ | 1.50 | 245.11324 | 0.17 | C_10_H_16_N_2_O_5_ | 1.27 | 3.80E-02 | 1.02 | Organic acids and derivatives |
| 13 | Norepinephrine sulfate | HMDB0002062 | [M+H]⁺ | 2.60 | 250.03809 | 0.42 | C_8_H_11_NO_6_S | 1.01 | 4.56E-02 | 1.32 | Organic acids and derivatives |
| 14 | Leucylleucine | HMDB0028933 | [M+H]⁺ | 5.15 | 245.18546 | -2.08 | C_12_H_24_N_2_O_3_ | 1.16 | 2.24E-02 | 1.23 | Organic acids and derivatives |
| 15 | N-Acetylhistamine | HMDB0013253 | [M+H]⁺ | 0.92 | 154.09712 | -2.39 | C_7_H_11_N_3_O | 1.43 | 6.34E-03 | 1.38 | Organic acids and derivatives |
| 16 | Valyllysine | HMDB0029132 | [M+H]⁺ | 0.79 | 246.18082 | -1.62 | C_11_H_23_N_3_O_3_ | 1.3 | 1.90E-02 | 1.01 | Organic acids and derivatives |
| 17 | DL-Dopa | HMDB0000609 | [M+H]⁺ | 5.11 | 198.07590 | -0.93 | C_9_H_11_NO_4_ | 1.41 | 5.84E-03 | 2.4 | Organic acids and derivatives |
| 18 | L-Tyrosine | HMDB0000158 | [M+H]⁺ | 5.25 | 182.08089 | -1.54 | C_9_H_11_NO_3_ | -2.69 | 1.32E-02 | 1.44 | Organic acids and derivatives |
| 19 | Valyltyrosine | HMDB0029139 | [M+H]⁺ | 3.24 | 281.14923 | -1.26 | C_14_H_20_N_2_O_4_ | 1.12 | 2.39E-02 | 1.51 | Organic acids and derivatives |
| 20 | Valylglutamine | HMDB0029125 | [M+H]⁺ | 1.79 | 246.14452 | -1.27 | C_10_H_19_N_3_O_4_ | 1.66 | 2.46E-03 | 1.46 | Organic acids and derivatives |
| 21 | Dopaquinone | HMDB0001229 | [M+H−H2O]⁺ | 5.02 | 178.04953 | -1.91 | C_9_H_9_NO_4_ | 1.26 | 3.40E-03 | 1.1 | [Organic acids and derivatives](http://classyfire.wishartlab.com/tax_nodes/C0000264) |
| 22 | 3-Dehydroquinic acid | HMDB0012710 | [M+H]⁺ | 1.38 | 191.05457 | -2.33 | C_7_H_10_O_6_ | 1.04 | 9.83E-03 | 1.06 | [Organic acids and derivatives](http://classyfire.wishartlab.com/tax_nodes/C0000264) |
| 23 | Uric acid | HMDB0000289 | [M−H]⁻ | 1.82 | 167.02040 | -3.97 | C_5_H_4_N_4_O_3_ | -1.57 | 1.12E-02 | 4.63 | Organoheterocyclic compounds |
| 24 | xanthurenic acid 8-O-sulfate | HMDB0059741 | [M−H]⁻ | 5.16 | 283.98706 | 0.05 | C_10_H_7_NO_7_S | 1.26 | 8.36E-05 | 3.82 | Organoheterocyclic compounds |
| 25 | 9-Methyluric acid | HMDB0001973 | [M−H]⁻ | 2.52 | 181.03609 | -3.44 | C_6_H_6_N_4_O_3_ | 1.41 | 2.43E-02 | 2.3 | Organoheterocyclic compounds |
| 26 | Xanthurenic acid | HMDB0000881 | [M−H]⁻ | 6.52 | 204.02942 | -2.99 | C_10_H_7_NO_4_ | 1.23 | 3.91E-02 | 1.88 | Organoheterocyclic compounds |
| 27 | 5-Hydroxyindoleacetic acid | HMDB0000763 | [M+H]⁺ | 6.10 | 192.06537 | -0.78 | C_10_H_9_NO_3_ | 2.49 | 9.09E-03 | 1.27 | Organoheterocyclic compounds |
| 28 | 5-Methylcytosine | HMDB0002894 | [M+H]⁺ | 0.91 | 126.06614 | -0.38 | C_5_H_7_N_3_O | 1.41 | 3.28E-02 | 3.96 | Organoheterocyclic compounds |
| 29 | 1-Methylguanine | HMDB0003282 | [M+H]⁺ | 1.34 | 166.07199 | -2.09 | C_6_H_7_N_5_O | 1.06 | 1.19E-02 | 2.96 | Organoheterocyclic compounds |
| 30 | Cytosine | HMDB0000630 | [M+H]⁺ | 1.32 | 112.05066 | 1.09 | C_4_H_5_N_3_O | 1.51 | 3.60E-02 | 2.37 | Organoheterocyclic compounds |
| 31 | 1-Methyluric acid | HMDB0003099 | [M+H]⁺ | 2.85 | 183.05113 | -0.75 | C_6_H_6_N_4_O_3_ | 1.46 | 6.41E-03 | 2.34 | Organoheterocyclic compounds |
| 32 | 6-Methyladenine | HMDB0002099 | [M+H]⁺ | 0.91 | 150.07706 | -2.41 | C_6_H_7_N_5_ | 1.56 | 2.88E-03 | 2.03 | Organoheterocyclic compounds |
| 33 | Xanthine | HMDB0000292 | [M+H]⁺ | 3.49 | 153.04059 | -0.73 | C_5_H_4_N_4_O_2_ | -1.16 | 1.54E-02 | 1.78 | [Organoheterocyclic compounds](http://classyfire.wishartlab.com/tax_nodes/C0000002) |
| 34 | N-Acetylserotonin | HMDB0001238 | [M+H]⁺ | 4.94 | 219.11226 | -2.48 | C_12_H_14_N_2_O_2_ | -1.3 | 3.85E-02 | 1.19 | [Organoheterocyclic compounds](http://classyfire.wishartlab.com/tax_nodes/C0000002) |
| 35 | Hypoxanthine | HMDB0000157 | [M+H]⁺ | 4.61 | 137.04565 | -1.00 | C_5_H_4_N_4_O | 2.52 | 7.41E-03 | 1.17 | Organoheterocyclic compounds |
| 36 | 6-Hydroxynicotinic acid | HMDB0002658 | [M+H]⁺ | 1.79 | 140.03412 | -0.71 | C_6_H_5_NO_3_ | 1.43 | 1.29E-02 | 1.04 | Organoheterocyclic compounds |
| 37 | Melatonin | HMDB0001389 | [M+H]⁺ | 5.75 | 233.12805 | -1.73 | C_13_H_16_N_2_O_2_ | -1.18 | 1.55E-03 | 1.06 | [Organoheterocyclic compounds](http://classyfire.wishartlab.com/tax_nodes/C0000002) |
| 38 | Pyridoxal | HMDB0001545 | [M+H]⁺ | 4.27 | 168.06531 | -1.25 | C_8_H_9_NO_3_ | -1.2 | 1.47E-03 | 1.88 | Organoheterocyclic compounds |
| 39 | Ascorbic acid | HMDB0000044 | [M−H]⁻ | 1.32 | 175.02371 | -6.29 | C_6_H_8_O_6_ | 1.01 | 7.14E-03 | 2.07 | [Organoheterocyclic compounds](http://classyfire.wishartlab.com/tax_nodes/C0000002) |
| 40 | Pimelic acid | HMDB0000857 | [M−H]⁻ | 5.47 | 159.06520 | -6.80 | C_7_H_12_O_4_ | 1.13 | 1.49E-02 | 3.24 | Lipids and lipid-like molecules |
| 41 | Palmitoleic Acid | HMDB0003229 | [M+H]⁺ | 13.95 | 255.23053 | -5.20 | C_16_H_30_O_2_ | 1.03 | 5.00E-02 | 2.07 | Lipids and lipid-like molecules |
| 42 | L-Acetylcarnitine | HMDB0000201 | [M+H]⁺ | 0.92 | 204.12256 | -2.32 | C_9_H_17_NO_4_ | 1.34 | 4.50E-03 | 1.06 | Lipids and lipid-like molecules |
| 43 | Estriol | HMDB0000153 | [M+H]⁺ | 5.72 | 289.17886 | -3.32 | C_18_H_24_O_3_ | 1.11 | 2.35E-02 | 4.15 | Lipids and lipid-like molecules |
| 44 | Prostaglandin B1 | HMDB0002982 | [M+H]⁺ | 11.91 | 337.23669 | -1.92 | C_20_H_32_O_4_ | 1.15 | 1.08E-02 | 2.26 | Lipids and lipid-like molecules |
| 45 | 3-Carboxy-4-methyl-5-propyl-2-furanpropionic acid | HMDB0061112 | [M−H]⁻ | 11.78 | 239.09241 | 5.50 | C_12_H_16_O_5_ | 1.93 | 2.62E-02 | 1.15 | Lipids and lipid-like molecules |
| 46 | Gentisic acid | HMDB0000152 | [M−H]⁻ | 6.48 | 153.01817 | -7.46 | C_7_H_6_O_4_ | -1.19 | 3.16E-03 | 2.65 | Benzenoids |
| 47 | Dopamine | HMDB0000073 | [M+H]⁺ | 1.50 | 154.08597 | -1.85 | C_8_H_11_NO_2_ | 1.12 | 2.79E-02 | 1.29 | Benzenoids |
| 48 | Salicyluric acid | HMDB0000840 | [M+H]⁺ | 4.99 | 196.06013 | -1.55 | C_9_H_9_NO_4_ | 1.51 | 1.94E-02 | 2.63 | Benzenoids |
| 49 | Pseudouridine | HMDB0000767 | [M−H]⁻ | 1.27 | 243.06232 | 0.25 | C_9_H_12_N_2_O_6_ | 1.23 | 1.37E-03 | 2.95 | Nucleosides, nucleotides, and analogues |
| 50 | Succinyladenosine | HMDB0000912 | [M−H]⁻ | 4.73 | 382.10025 | -0.49 | C_14_H_17_N_5_O_8_ | 1.19 | 1.59E-02 | 1.64 | Nucleosides, nucleotides, and analogues |
| 51 | Xanthosine | HMDB0000299 | [M−H]⁻ | 3.47 | 283.06860 | 0.68 | C_10_H_12_N_4_O_6_ | 1.63 | 2.45E-02 | 1.03 | Nucleosides, nucleotides, and analogues |
| 52 | 5-Methyldeoxycytidine | HMDB0002224 | [M+H]⁺ | 1.41 | 242.11292 | -2.53 | C_10_H_15_N_3_O_4_ | 1.13 | 4.32E-02 | 3.33 | Nucleosides, nucleotides, and analogues |
| 53 | Cyclic AMP | HMDB0000058 | [M+H]⁺ | 1.50 | 330.05978 | -0.05 | C_10_H_12_N_5_O_6_P | 1.13 | 1.02E-02 | 2.25 | Nucleosides, nucleotides, and analogues |
| 54 | 7-Methylguanosine | HMDB0001107 | [M+H]⁺ | 4.68 | 299.12283 | 1.37 | C_11_H_16_N_5_O_5_ | 1.4 | 2.10E-02 | 2.05 | Nucleosides, nucleotides, and analogues |
| 55 | N4-Acetylcytidine | HMDB0005923 | [M+H]⁺ | 4.23 | 286.10361 | 0.87 | C_11_H_15_N_3_O_6_ | 1.76 | 5.68E-03 | 1.69 | Nucleosides, nucleotides, and analogues |
| 56 | 3'-AMP | HMDB0003540 | [M+H]⁺ | 1.40 | 348.07028 | -0.23 | C_10_H_14_N_5_O_7_P | 1.04 | 1.33E-02 | 1.09 | Nucleosides, nucleotides, and analogues |
| 57 | Porphobilinogen | HMDB0000245 | [M−H]⁻ | 4.59 | 225.08788 | -0.89 | C_10_H_14_N_2_O_4_ | 1.1 | 2.42E-02 | 1.28 | Organic nitrogen compounds |
| 58 | Sphinganine | HMDB0000269 | [M+H]⁺ | 22.23 | 302.30511 | -0.81 | C_18_H_39_NO_2_ | 1.03 | 2.70E-02 | 2.8 | Organic nitrogen compounds |
| 59 | Spermine | HMDB0001256 | [M+H]⁺ | 0.71 | 203.22273 | -1.44 | C_10_H_26_N_4_ | 1.08 | 3.74E-02 | 1.57 | Organic nitrogen compounds |
| 60 | N-Acetylgalactosamine 4-sulphate | HMDB0000781 | [M−H]⁻ | 11.04 | 300.03949 | 0.05 | C_8_H_15_NO_9_S | 1.42 | 2.19E-02 | 3.13 | Organic oxygen compounds |
| 61 | N-Acetylneuraminic acid | HMDB0000230 | [M−H]⁻ | 1.06 | 308.09906 | 1.15 | C_11_H_19_NO_9_ | 1.02 | 8.40E-04 | 2.23 | Organic oxygen compounds |
| 62 | Creatine riboside | HMDB0240254 | [M+H−H2O]⁺ | 3.38 | 246.10846 | 0.05 | C_9_H_17_N_3_O_6_ | 1.02 | 4.58E-02 | 1.52 | Organic oxygen compounds |
|  | B：JBHD-L *vs.* Model |  |  |  |  |  |  |  |  |  |  |
| 1 | D-Pipecolic acid | HMDB0005960 | [M+H]⁺ | 1.32 | 130.08609 | -1.27 | C_6_H_11_NO_2_ | 2.2 | 6.87E-09 | 8.64 | Organic acids and derivatives |
| 2 | Carnosine | HMDB0000033 | [M+H]⁺ | 4.52 | 227.11328 | -2.58 | C_9_H_14_N_4_O_3_ | 10.1 | 8.28E-09 | 2.15 | Organic acids and derivatives |
| 3 | L-Cysteine | HMDB0000574 | [M+H]⁺ | 12.58 | 122.02711 | 0.69 | C_3_H_7_NO_2_S | 8.93 | 3.93E-05 | 1.27 | [Organic acids and derivatives](http://classyfire.wishartlab.com/tax_nodes/C0000264) |
| 4 | L-Tyrosine | HMDB0000158 | [M+H]⁺ | 5.25 | 182.08089 | -1.54 | C_9_H_11_NO_3_ | 1.47 | 5.85E-03 | 1.02 | Organic acids and derivatives |
| 5 | 3-Hydroxysebacic acid | HMDB0000350 | [M+H]⁺ | 4.03 | 219.12236 | -1.55 | C_10_H_18_O_5_ | 8.11 | 4.17E-04 | 1.34 | Organic acids and derivatives |
| 6 | N-Benzoylaspartic acid | HMDB0034251 | [M+H]⁺ | 5.93 | 238.07104 | 0.17 | C_11_H_11_NO_5_ | 2.83 | 7.31E-07 | 1.12 | Organic acids and derivatives |
| 7 | Citrulline | HMDB0000904 | [M+H]⁺ | 0.90 | 176.10262 | -1.98 | C_6_H_13_N_3_O_3_ | 1.34 | 7.64E-05 | 1.1 | Organic acids and derivatives |
| 8 | Pipecolic acid | HMDB0000070 | [M+H]⁺ | 1.32 | 130.08609 | -1.27 | C_6_H_11_NO_2_ | 1.4 | 2.36E-05 | 1.28 | Organic acids and derivatives |
| 9 | N-Acetyl-L-glutamic acid | HMDB0001138 | [M−H]⁻ | 6.31 | 188.05656 | 0.61 | C_7_H_11_NO_5_ | 4.32 | 1.91E-07 | 2.74 | Organic acids and derivatives |
| 10 | 3-Hydroxysuberic acid | HMDB0000325 | [M−H]⁻ | 6.39 | 189.07591 | -4.95 | C_8_H_14_O_5_ | 4.04 | 7.06E-11 | 1.62 | Organic acids and derivatives |
| 11 | Fumaric acid | HMDB0000134 | [M−H]⁻ | 0.90 | 115.00237 | -1.87 | C_4_H_4_O_4_ | 1.14 | 8.75E-03 | 1.01 | Organic acids and derivatives |
| 12 | 2-Methoxybenzoic acid | HMDB0032604 | [M+H]+ | 6.16 | 153.05446 | -1.05 | C_8_H_8_O_3_ | 2.7 | 3.74E-12 | 2.49 | Benzenoids |
| 13 | Alpha-Hydroxyhippuric acid | HMDB0002404 | [M+H]⁺ | 6.72 | 196.06015 | -1.45 | C_9_H_9_NO_4_ | 3.9 | 1.57E-09 | 1.89 | Benzenoids |
| 14 | 2-Methylhippuric acid | HMDB0011723 | [M+H]⁺ | 6.04 | 194.08076 | -2.11 | C_10_H_11_NO_3_ | 2.25 | 2.26E-07 | 1.87 | Benzenoids |
| 15 | Phenylacetaldehyde | HMDB0006236 | [M−H]⁻ | 5.61 | 119.04893 | -10.99 | C_8_H_8_O | -1 | 7.44E-03 | 2.64 | Benzenoids |
| 16 | Epinephrine | HMDB0000068 | [M−H]⁻ | 6.52 | 182.08177 | -2.73 | C_9_H_13_NO_3_ | 5.26 | 4.47E-03 | 1.62 | Benzenoids |
| 17 | Gentisic acid | HMDB0000152 | [M−H]⁻ | 6.48 | 153.01817 | -7.46 | C_7_H_6_O_4_ | 1.59 | 1.38E-06 | 3.46 | Benzenoids |
| 18 | 2-Methylbenzoic acid | HMDB0002340 | [M−H]⁻ | 9.05 | 135.04395 | -8.91 | C_8_H_8_O_2_ | 2.35 | 1.36E-03 | 1.48 | Benzenoids |
| 19 | 3,4-Dihydroxybenzeneacetic acid | HMDB0001336 | [M−H]⁻ | 5.65 | 167.03391 | -6.42 | C_8_H_8_O_4_ | 8.26 | 5.84E-08 | 2.58 | [Benzenoids](http://classyfire.wishartlab.com/tax_nodes/C0002448) |
| 20 | Gallic acid | HMDB0005807 | [M−H]⁻ | 2.54 | 169.01341 | -4.95 | C_7_H_6_O_5_ | 7.73 | 5.47E-03 | 1.26 | [Benzenoids](http://classyfire.wishartlab.com/tax_nodes/C0002448) |
| 21 | Mandelic acid | HMDB0000703 | [M−H]⁻ | 5.38 | 151.03894 | -7.46 | C_8_H_8_O_3_ | 2.78 | 3.35E-05 | 1.35 | Benzenoids |
| 22 | Pyridoxamine | HMDB0001431 | [M+H]⁺ | 3.91 | 169.09686 | -1.74 | C_8_H_12_N_2_O_2_ | 1.12 | 4.55E-05 | 3.74 | Organoheterocyclic compounds |
| 23 | Kojic acid | HMDB0032923 | [M+H]⁺ | 4.39 | 143.03375 | -0.95 | C_6_H_6_O_4_ | 1.49 | 4.41E-06 | 2.63 | Organoheterocyclic compounds |
| 24 | Picolinic acid | HMDB0002243 | [M+H]⁺ | 4.48 | 124.03940 | 0.77 | C_6_H_5_NO_2_ | 1.52 | 2.28E-07 | 3.03 | Organoheterocyclic compounds |
| 25 | 5-Hydroxyindoleacetic acid | HMDB0000763 | [M+H]⁺ | 6.10 | 192.06537 | -0.78 | C_10_H_9_NO_3_ | -1.96 | 5.42E-06 | 1.55 | Organoheterocyclic compounds |
| 26 | 2-Furoic acid | HMDB0000617 | [M−H]⁻ | 0.97 | 111.00742 | -12.14 | C_5_H_4_O_3_ | 1.33 | 2.80E-03 | 5.35 | Organoheterocyclic compounds |
| 27 | Xanthurenic acid | HMDB0000881 | [M−H]⁻ | 6.52 | 204.02942 | -2.99 | C_10_H_7_NO_4_ | -1.47 | 4.54E-04 | 1.35 | Organoheterocyclic compounds |
| 28 | D-Glucurono-6,3-lactone | HMDB0006355 | [M−H]⁻ | 11.80 | 175.02403 | -4.46 | C_6_H_8_O_6_ | 2.03 | 2.57E-04 | 1.14 | Organoheterocyclic compounds |
| 29 | Uric acid | HMDB0000289 | [M−H]⁻ | 1.82 | 167.02040 | -3.97 | C_5_H_4_N_4_O_3_ | 1.22 | 1.13E-06 | 1.52 | Organoheterocyclic compounds |
| 30 | xanthurenic acid 8-O-sulfate | HMDB0059741 | [M−H]⁻ | 5.16 | 283.98706 | 0.05 | C_10_H_7_NO_7_S | -1.02 | 6.83E-05 | 1.39 | Organoheterocyclic compounds |
| 31 | 2,4,6-Octatriynoic acid | HMDB0030967 | [M+H]⁺ | 12.57 | 133.02834 | -0.50 | C_8_H_4_O_2_ | 7.44 | 4.57E-04 | 1.01 | Lipids and lipid-like molecules |
| 32 | Itaconic acid | HMDB0002092 | [M−H]⁻ | 1.00 | 129.01808 | -9.70 | C_5_H_6_O_4_ | 1.92 | 3.69E-05 | 3.35 | Lipids and lipid-like molecules |
| 33 | 2-Isopropylmalic acid | HMDB0000402 | [M−H]⁻ | 5.26 | 175.06012 | -6.15 | C_7_H_12_O_5_ | 3.05 | 1.68E-09 | 4.37 | Lipids and lipid-like molecules |
| 34 | 9-Oxo-nonanoic acid | HMDB0094711 | [M−H]⁻ | 6.97 | 171.10175 | -5.36 | C_9_H_16_O_3_ | 1.44 | 6.66E-03 | 2.16 | Lipids and lipid-like molecules |
| 35 | 3,3-Dimethylglutaric acid | HMDB0002441 | [M−H]⁻ | 6.30 | 159.06537 | -5.73 | C_7_H_12_O_4_ | 1.2 | 2.98E-04 | 1.72 | Lipids and lipid-like molecules |
| 36 | Galacturonic acid | HMDB0002545 | [M−H]⁻ | 7.91 | 193.03482 | -2.88 | C_6_H_10_O_7_ | 4.9 | 3.47E-07 | 1.32 | Organic oxygen compounds |
| 37 | N-Acetylneuraminic acid | HMDB0000230 | [M−H]⁻ | 1.06 | 308.09906 | 1.15 | C_11_H_19_NO_9_ | -1.17 | 3.72E-08 | 1.59 | Organic oxygen compounds |
| 38 | Cinnamic acid | HMDB0000567 | [M+H]⁺ | 5.02 | 149.05942 | -1.92 | C_9_H_8_O_2_ | 3.9 | 9.52E-04 | 3.06 | Phenylpropanoids and polyketides |
| 39 | 3'-AMP | HMDB0003540 | [M+H]⁺ | 1.40 | 348.07028 | -0.23 | C_10_H_14_N_5_O_7_P | -1.78 | 2.85E-04 | 1.02 | Nucleosides, nucleotides, and analogues |
| 40 | Histamine | HMDB0000870 | [M+H]⁺ | 1.39 | 112.08703 | 0.95 | C_5_H_9_N_3_ | 6.14 | 3.30E-06 | 1.8 | [Organic nitrogen compounds](http://classyfire.wishartlab.com/tax_nodes/C0004707) |
|  | C：JBHD-H *vs.* Model |  |  |  |  |  |  |  |  |  |  |
| 1 | D-Pipecolic acid | HMDB0005960 | [M+H]⁺ | 1.32 | 130.08609 | -1.27 | C_6_H_11_NO_2_ | 2.62 | 6.43E-09 | 9.46 | Organic acids and derivatives |
| 2 | 4-Guanidinobutanoic acid | HMDB0003464 | [M+H]⁺ | 1.32 | 146.09209 | -2.14 | C_5_H_11_N_3_O_2_ | 1.26 | 1.49E-05 | 7.01 | [Organic acids and derivatives](http://classyfire.wishartlab.com/tax_nodes/C0000264) |
| 3 | Carnosine | HMDB0000033 | [M+H]⁺ | 4.52 | 227.11328 | -2.58 | C_9_H_14_N_4_O_3_ | 10.53 | 2.88E-09 | 3.05 | Organic acids and derivatives |
| 4 | L-Cysteine | HMDB0000574 | [M+H]⁺ | 12.58 | 122.02711 | 0.69 | C_3_H_7_NO_2_S | 9.58 | 4.93E-05 | 1.49 | [Organic acids and derivatives](http://classyfire.wishartlab.com/tax_nodes/C0000264) |
| 5 | L-Tyrosine | HMDB0000158 | [M+H]⁺ | 5.25 | 182.08089 | -1.54 | C_9_H_11_NO_3_ | 1.92 | 8.55E-04 | 1.01 | Organic acids and derivatives |
| 6 | 3-Hydroxysebacic acid | HMDB0000350 | [M+H]⁺ | 4.03 | 219.12236 | -1.55 | C_10_H_18_O_5_ | 8.45 | 7.15E-05 | 1.86 | Organic acids and derivatives |
| 7 | Citrulline | HMDB0000904 | [M+H]⁺ | 0.90 | 176.10262 | -1.98 | C_6_H_13_N_3_O_3_ | 1.7 | 8.84E-06 | 1.3 | Organic acids and derivatives |
| 8 | Pipecolic acid | HMDB0000070 | [M+H]⁺ | 1.32 | 130.08609 | -1.27 | C_6_H_11_NO_2_ | 1.74 | 4.63E-06 | 1.47 | Organic acids and derivatives |
| 9 | N-Acetyl-L-glutamic acid | HMDB0001138 | [M−H]⁻ | 6.31 | 188.05656 | 0.61 | C_7_H_11_NO_5_ | 1.12 | 7.33E-05 | 1.06 | Organic acids and derivatives |
| 10 | Citric acid | HMDB0000094 | [M−H]⁻ | 0.97 | 191.01871 | -5.32 | C_6_H_8_O_7_ | -1.34 | 1.07E-05 | 16.1 | Organic acids and derivatives |
| 11 | 3-Hydroxysuberic acid | HMDB0000325 | [M−H]⁻ | 6.39 | 189.07591 | -4.95 | C_8_H_14_O_5_ | 5.47 | 2.72E-07 | 1.94 | Organic acids and derivatives |
| 12 | Fumaric acid | HMDB0000134 | [M−H]⁻ | 0.90 | 115.00237 | -1.87 | C_4_H_4_O_4_ | 1.81 | 5.17E-05 | 1.16 | Organic acids and derivatives |
| 13 | Taurine | HMDB0000251 | [M+H]⁺ | 0.85 | 126.02191 | -0.24 | C_2_H_7_NO_3_S | -1.87 | 2.36E-04 | 3.93 | Organic acids and derivatives |
| 14 | Homovanillic acid sulfate | HMDB0011719 | [M−H]⁻ | 5.07 | 261.00699 | -1.75 | C_9_H_10_O_7_S | -1.01 | 1.47E-06 | 3.17 | Organic acids and derivatives |
| 15 | Isocitric acid | HMDB0000193 | [M−H]⁻ | 0.94 | 191.01880 | -4.85 | C_6_H_8_O_7_ | -1.19 | 1.03E-02 | 13.02 | Organic acids and derivatives |
| 16 | Hippuric acid | HMDB0000714 | [M+H]⁺ | 5.56 | 180.06499 | -2.94 | C_9_H_9_NO_3_ | 1.1 | 1.98E-08 | 18.19 | Benzenoids |
| 17 | 2-Methoxybenzoic acid | HMDB0032604 | [M+H]⁺ | 6.16 | 153.05446 | -1.05 | C_8_H_8_O_3_ | 3.14 | 2.64E-12 | 2.88 | Benzenoids |
| 18 | Alpha-Hydroxyhippuric acid | HMDB0002404 | [M+H]⁺ | 6.72 | 196.06015 | -1.45 | C_9_H_9_NO_4_ | 4.36 | 1.57E-10 | 2.89 | Benzenoids |
| 19 | 2-Methylhippuric acid | HMDB0011723 | [M+H]⁺ | 6.04 | 194.08076 | -2.11 | C_10_H_11_NO_3_ | 2.66 | 2.95E-08 | 1.98 | Benzenoids |
| 20 | Vanillic acid 4-O-sulfate | HMDB0041788 | [M−H]⁻ | 5.07 | 246.99124 | -2.25 | C_8_H_8_O_7_S | 3.09 | 3.28E-06 | 11.51 | Benzenoids |
| 21 | Phenylacetaldehyde | HMDB0006236 | [M−H]⁻ | 5.61 | 119.04893 | -10.99 | C_8_H_8_O | -1.02 | 1.57E-08 | 4.13 | Benzenoids |
| 22 | Gentisic acid | HMDB0000152 | [M−H]⁻ | 6.48 | 153.01817 | -7.46 | C_7_H_6_O_4_ | 2.39 | 1.36E-07 | 3.66 | Benzenoids |
| 23 | 2-Methylbenzoic acid | HMDB0002340 | [M−H]⁻ | 9.05 | 135.04395 | -8.91 | C_8_H_8_O_2_ | 3.75 | 5.26E-05 | 1.94 | Benzenoids |
| 24 | 3,4-Dihydroxybenzeneacetic acid | HMDB0001336 | [M−H]⁻ | 5.65 | 167.03391 | -6.42 | C_8_H_8_O_4_ | 8.66 | 1.48E-06 | 2.16 | [Benzenoids](http://classyfire.wishartlab.com/tax_nodes/C0002448) |
| 25 | Gallic acid | HMDB0005807 | [M−H]⁻ | 2.54 | 169.01341 | -4.95 | C_7_H_6_O_5_ | 8.43 | 3.49E-02 | 1.25 | [Benzenoids](http://classyfire.wishartlab.com/tax_nodes/C0002448) |
| 26 | Mandelic acid | HMDB0000703 | [M−H]⁻ | 5.38 | 151.03894 | -7.46 | C_8_H_8_O_3_ | 3.04 | 7.57E-07 | 1.08 | Benzenoids |
| 27 | Pyridoxamine | HMDB0001431 | [M+H]⁺ | 3.91 | 169.09686 | -1.74 | C_8_H_12_N_2_O_2_ | 1.24 | 3.25E-05 | 3.41 | Organoheterocyclic compounds |
| 28 | Kojic acid | HMDB0032923 | [M+H]+ | 4.39 | 143.03375 | -0.95 | C_6_H_6_O_4_ | 2.08 | 9.41E-06 | 2.3 | Organoheterocyclic compounds |
| 29 | Picolinic acid | HMDB0002243 | [M+H]⁺ | 4.48 | 124.03940 | 0.77 | C_6_H_5_NO_2_ | 1.96 | 4.28E-07 | 2.23 | Organoheterocyclic compounds |
| 30 | Urocanic acid | HMDB0000301 | [M+H]⁺ | 1.32 | 139.04997 | -1.68 | C_6_H_6_N_2_O_2_ | 1.28 | 5.25E-05 | 1.59 | Organoheterocyclic compounds |
| 31 | Pyridoxal | HMDB0001545 | [M+H]⁺ | 4.27 | 168.06531 | -1.25 | C_8_H_9_NO_3_ | 1.05 | 4.45E-04 | 1.23 | Organoheterocyclic compounds |
| 32 | 5-Hydroxyindoleacetic acid | HMDB0000763 | [M+H]⁺ | 6.10 | 192.06537 | -0.78 | C_10_H_9_NO_3_ | -1.39 | 4.62E-07 | 1.61 | Organoheterocyclic compounds |
| 33 | Folinic acid | HMDB0001562 | [M+H]⁺ | 4.99 | 474.17294 | -0.49 | C_20_H_23_N_7_O_7_ | 1.6 | 9.59E-03 | 1.08 | Organoheterocyclic compounds |
| 34 | Nicotinic acid | HMDB0001488 | [M+H]⁺ | 4.48 | 124.03940 | 0.77 | C_6_H_5_NO_2_ | 2.16 | 8.58E-06 | 1.05 | Organoheterocyclic compounds |
| 35 | 4-Pyridoxic acid | HMDB0000017 | [M−H]⁻ | 2.09 | 182.04521 | -3.69 | C_8_H_9_NO_4_ | 1.18 | 1.03E-02 | 1.92 | Organoheterocyclic compounds |
| 36 | Ethylmalonic acid | HMDB0000622 | [M−H]⁻ | 4.16 | 131.03384 | -8.72 | C_5_H_8_O_4_ | 1.46 | 5.61E-06 | 1.75 | Organoheterocyclic compounds |
| 37 | Uric acid | HMDB0000289 | [M−H]⁻ | 1.82 | 167.02040 | -3.97 | C_5_H_4_N_4_O_3_ | 1.15 | 4.82E-06 | 3 | Organoheterocyclic compounds |
| 38 | Xanthurenic acid | HMDB0000881 | [M−H]⁻ | 6.52 | 204.02942 | -2.99 | C_10_H_7_NO_4_ | -1.13 | 5.40E-04 | 1.22 | Organoheterocyclic compounds |
| 39 | 9-Methyluric acid | HMDB0001973 | [M−H]⁻ | 2.52 | 181.03609 | -3.44 | C_6_H_6_N_4_O_3_ | -1.06 | 1.85E-06 | 1.44 | Organoheterocyclic compounds |
| 40 | 2,4,6-Octatriynoic acid | HMDB0030967 | [M+H]⁺ | 12.57 | 133.02834 | -0.50 | C_8_H_4_O_2_ | 8.05 | 5.74E-05 | 1.11 | Lipids and lipid-like molecules |
| 41 | Itaconic acid | HMDB0002092 | [M−H]⁻ | 1.00 | 129.01808 | -9.70 | C_5_H_6_O_4_ | 2.92 | 1.32E-04 | 3.72 | Lipids and lipid-like molecules |
| 42 | 2-Isopropylmalic acid | HMDB0000402 | [M−H]⁻ | 5.26 | 175.06012 | -6.15 | C_7_H_12_O_5_ | 3.83 | 1.72E-03 | 3.84 | Lipids and lipid-like molecules |
| 43 | 3,3-Dimethylglutaric acid | HMDB0002441 | [M−H]⁻ | 6.30 | 159.06537 | -5.73 | C_7_H_12_O_4_ | 1.37 | 1.20E-05 | 1.38 | Lipids and lipid-like molecules |
| 44 | Citraconic acid | HMDB0000634 | [M−H]⁻ | 3.65 | 129.01825 | -8.39 | C_5_H_6_O_4_ | 1.92 | 1.24E-04 | 1.1 | Lipids and lipid-like molecules |
| 45 | Sebacic acid | HMDB0000792 | [M−H]⁻ | 11.66 | 201.11250 | -3.64 | C_10_H_18_O_4_ | 1.45 | 1.61E-06 | 1.47 | Lipids and lipid-like molecules |
| 46 | Suberic acid | HMDB0000893 | [M−H]⁻ | 6.62 | 173.08107 | -4.98 | C_8_H_14_O_4_ | 1.32 | 2.95E-02 | 1.46 | Lipids and lipid-like molecules |
| 47 | Trehalose | HMDB0000975 | [M−H]⁻ | 0.91 | 341.10855 | -1.13 | C_12_H_22_O_11_ | 5.65 | 1.70E-04 | 1.7 | Organic oxygen compounds |
| 48 | Galacturonic acid | HMDB0002545 | [M−H]⁻ | 7.91 | 193.03482 | -2.88 | C_6_H_10_O_7_ | 6.09 | 5.37E-07 | 1.45 | Organic oxygen compounds |
| 49 | Benzoyl glucuronide (Benzoic acid) | HMDB0010324 | [M−H]⁻ | 4.73 | 297.06146 | 3.25 | C_13_H_14_O_8_ | 1.42 | 5.76E-06 | 1.56 | Organic oxygen compounds |
| 50 | N-Acetylgalactosamine 4-sulphate | HMDB0000781 | [M−H]⁻ | 11.04 | 300.03949 | 0.05 | C_8_H_15_NO_9_S | -1.41 | 3.79E-07 | 1.2 | Organic oxygen compounds |
| 51 | Glucaric acid | HMDB0000663 | [M−H]⁻ | 0.87 | 209.02942 | 1.08 | C_6_H_10_O_8_ | -2.37 | 2.22E-06 | 1.9 | Organic oxygen compounds |
| 52 | 4-(2-Aminophenyl)-2,4-dioxobutanoic acid | HMDB0000978 | [M−H]⁻ | 5.33 | 206.04523 | 2.16 | C_10_H_9_NO_4_ | -1.44 | 1.53E-05 | 1.23 | Organic oxygen compounds |
| 53 | 5'-Methylthioadenosine | HMDB0001173 | [M+H]⁺ | 4.95 | 298.09671 | -0.42 | C_11_H_15_N_5_O_3_S | 1.42 | 2.73E-03 | 1.39 | Nucleosides, nucleotides, and analogues |
| 54 | Pseudouridine | HMDB0000767 | [M−H]⁻ | 1.27 | 243.06232 | 0.25 | C_9_H_12_N_2_O_6_ | -1.62 | 9.54E-08 | 1.59 | Nucleosides, nucleotides, and analogues |
| 55 | Cinnamic acid | HMDB0000567 | [M+H]⁺ | 5.02 | 149.05942 | -1.92 | C_9_H_8_O_2_ | 3.97 | 7.62E-04 | 3.4 | Phenylpropanoids and polyketides |
| 56 | Histamine | HMDB0000870 | [M+H]⁺ | 1.39 | 112.08703 | 0.95 | C_5_H_9_N_3_ | 6.47 | 3.80E-07 | 1.44 | [Organic nitrogen compounds](http://classyfire.wishartlab.com/tax_nodes/C0004707) |

Abbreviations: DEMs, differentially expressed metabolites; JBHD-L and JBHD-H, Jiawei Buyang Huanwu Decoction at low (10 g/kg) and high (20 g/kg) doses, respectively.

Supplementary Table 4 DEMs composition in serum samples

| Metabolite Superclass | Model *vs.* Control | JBHD-L *vs.* Model | JBHD-H *vs.* Model |
| --- | --- | --- | --- |
|  | (Count ^a^/ Percent ^b^) | | |
| Lipids and lipid-like molecules | 23 / 57.50% | 16 / 50.00% | 15 / 44.12% |
| Organic acids and derivatives | 9/ 22.50% | 10 / 31.25% | 13 / 38.24% |
| Organoheterocyclic compounds | 6 / 15.00% | 3 / 9.38% | 1 / 2.94% |
| Organic nitrogen compounds | 2 / 5.00% | / | 2/ 5.88% |
| Benzenoids | / | 3 / 9.38% | 2 / 5.88% |
| Organic oxygen compounds | / | / | 1 / 2.94% |

Notes: a, number of metabolites contained in a super class; b, number of DEMs belonging to a given classification as a percentage of the total number of DEMs in two comparison groups.

Abbreviations: DEMs, differentially expressed metabolites; JBHD-L and JBHD-H, Jiawei Buyang Huanwu Decoction at low (10 g/kg) and high (20 g/kg) doses, respectively.

Supplementary Table 5 DEMs composition in urine samples

| Metabolite Superclass | Model vs. Control | JBHD-L vs. Model | JBHD-H vs. Model |
| --- | --- | --- | --- |
|  | (Count ^a^ / Percent ^b^) | | |
| [Organic acids and derivatives](http://classyfire.wishartlab.com/tax_nodes/C0000264) | 22 / 35.48% | 11 / 27.50% | 15 / 26.79% |
| Organoheterocyclic compounds | 17 / 27.42% | 9 / 22.50% | 13 / 23.21% |
| Nucleosides, nucleotides, and analogues | 8/ 12.90% | 1 / 2.50% | 2 / 3.57% |
| Lipids and lipid-like molecules | 6/ 9.68% | 5 / 12.50% | 7 / 12.50% |
| Organic oxygen compounds | 6/ 9.68% | 2 / 5.00% | 6 / 10.71% |
| Benzenoids | 3 / 4.84% | 10 / 25.00% | 11 / 19.64% |
| [Organic nitrogen compounds](http://classyfire.wishartlab.com/tax_nodes/C0004707) | / | 1 / 2.50% | 1 / 1.79% |
| Phenylpropanoids and polyketides | / | 1 / 2.50% | 1 / 1.79% |

Notes: a, number of metabolites contained in a super class; b, number of DEMs belonging to a given classification as a percentage of the total number of DEMs in two comparison groups.

Abbreviations: DEMs, differentially expressed metabolites;JBHD-L and JBHD-H, Jiawei Buyang Huanwu Decoction at low (10 g/kg) and high (20 g/kg) doses, respectively.

Supplementary Table 6 Network centrality of DAFMs correlated with serum DEMs

| Node_Name | Degree_Centrality | Closeness_Centrality | Betweenness_Centrality |
| --- | --- | --- | --- |
| *Lactobacillus* | 0.6154 | 0.65 | 0.2248 |
| *Clostridium* sensu stricto 1 | 0.5385 | 0.5909 | 0.2536 |
| *Turicibacter* | 0.5385 | 0.5909 | 0.1608 |
| Christensenellaceae R-7 group | 0.3846 | 0.5 | 0.1942 |
| L-Norleucine | 0.2308 | 0.5 | 0.0208 |
| LysoPC(22:5(7Z,10Z,13Z,16Z,19Z)/0:0) | 0.2308 | 0.5 | 0.0208 |
| Platelet-activating factor | 0.2308 | 0.5 | 0.0208 |
| Citrulline | 0.2308 | 0.5 | 0.0413 |
| Stearic acid | 0.2308 | 0.5 | 0.0413 |
| Muricholic acid | 0.3077 | 0.5909 | 0.1004 |
| Deoxycholic acid | 0.2308 | 0.5417 | 0.067 |
| 7-ketodeoxycholic acid | 0.2308 | 0.5 | 0.0208 |
| Dihydrothymine | 0.0769 | 0.3824 | 0 |
| Cholic acid | 0.0769 | 0.3421 | 0 |

Supplementary Table 7 Network centrality of DAFMs correlated with urine DEMs

| Node_Name | Degree_Centrality | Closeness_Centrality | Betweenness_Centrality |
| --- | --- | --- | --- |
| *Lactobacillus* | 0.75 | 0.7619 | 0.3165 |
| *Clostridium* sensu stricto 1 | 0.6875 | 0.6957 | 0.1947 |
| *Turicibacter* | 0.6875 | 0.6957 | 0.1802 |
| Christensenellaceae R-7 group | 0.4375 | 0.5161 | 0.0586 |
| 3'-AMP | 0.125 | 0.5 | 0.0026 |
| Pyridoxal | 0.1875 | 0.5333 | 0.0069 |
| Xanthurenic acid | 0.0625 | 0.4444 | 0 |
| N-Acetylneuraminic acid | 0.1875 | 0.5333 | 0.0069 |
| Pseudouridine | 0.25 | 0.5714 | 0.0199 |
| Uric acid | 0.25 | 0.5714 | 0.0199 |
| 9-Methyluric acid | 0.1875 | 0.5333 | 0.0115 |
| Gentisic acid | 0.1875 | 0.5333 | 0.0069 |
| Homovanillic acid sulfate | 0.25 | 0.5714 | 0.0199 |
| 5-Hydroxyindoleacetic acid | 0.25 | 0.5714 | 0.0199 |
| N-Acetylgalactosamine 4-sulphate | 0.1875 | 0.5333 | 0.0069 |
| L-Tyrosine | 0.25 | 0.5714 | 0.0199 |
| xanthurenic acid 8-O-sulfate | 0.1875 | 0.5 | 0.0087 |


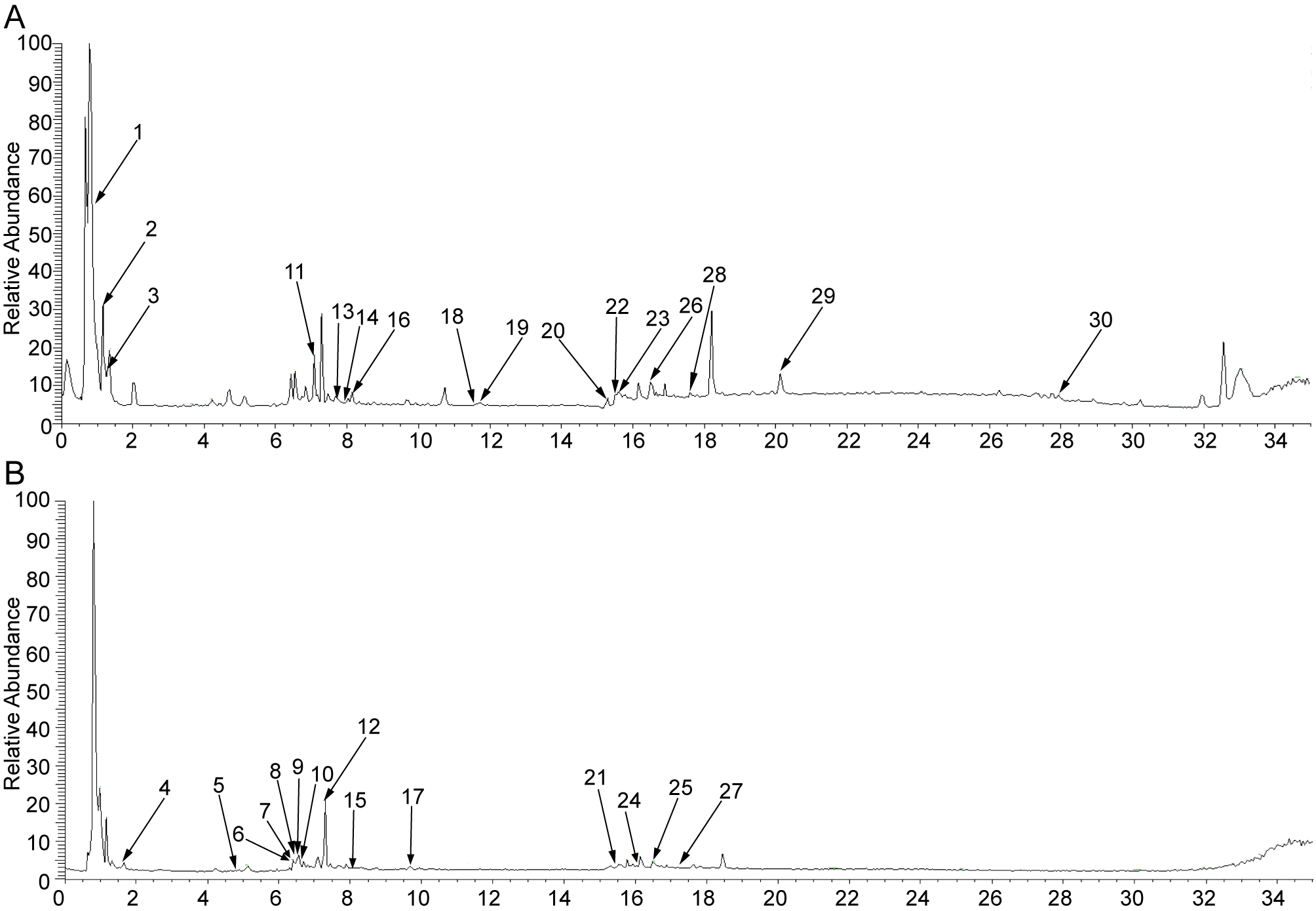


Supplementary Figure 1 Total ion chromatogram of JBHD by UPLC-MS/MS. (A) Positive ion mode. (B) Negative ion mode. For details of the 30 authentic standards, see the Supplementary Table S1.


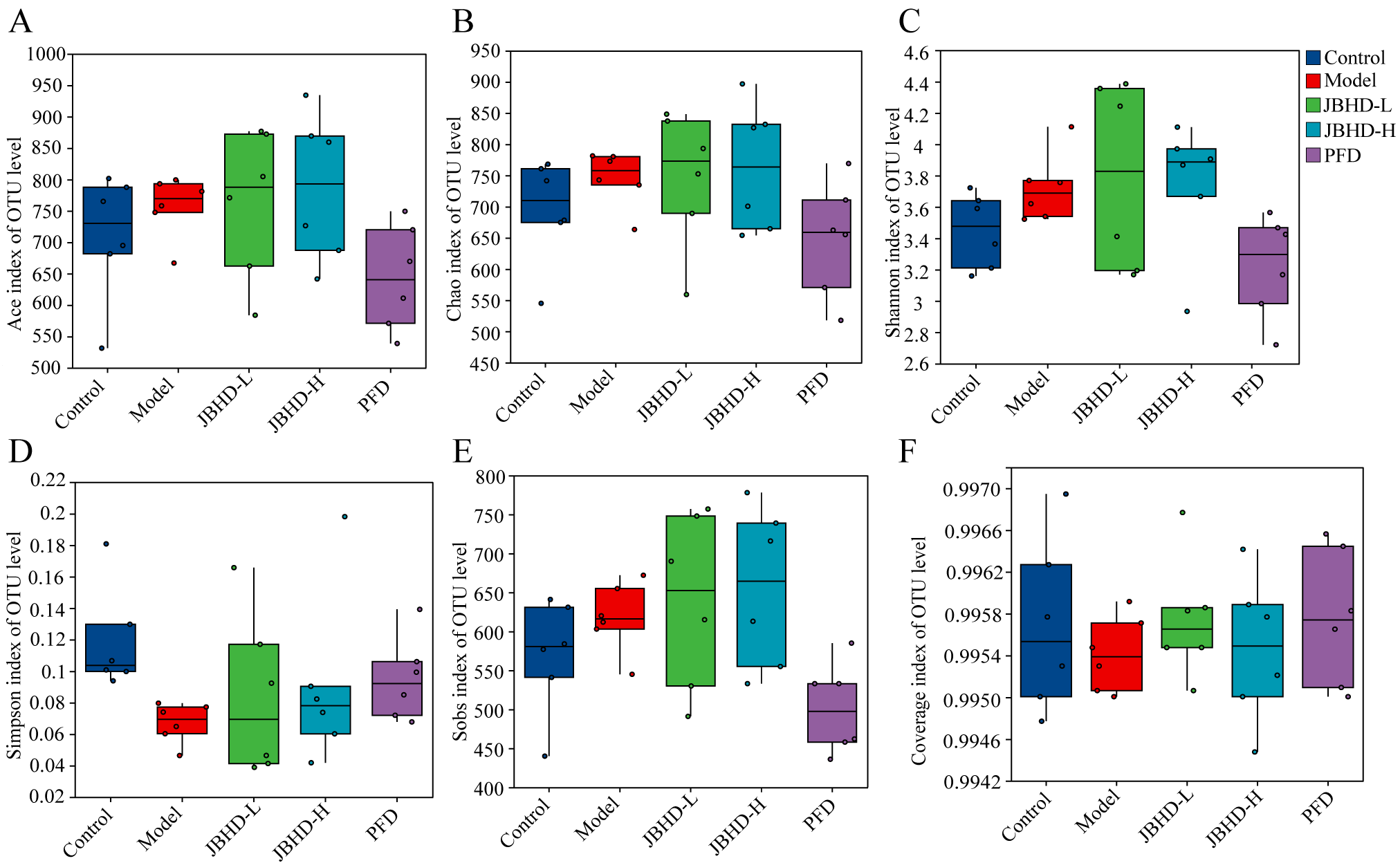


Supplementary Figure 2 Alpha diversity analysis. (A) Ace index. (B) Chao index. (C) Shannon index. (D) Simpson index. (E) Sobs index. (F) Coverage index. JBHD-L and JBHD-H, Jiawei Buyang Huanwu Decoction at low (10 g/kg) and high (20 g/kg) doses, respectively; PFD, Pirfenidone capsules.


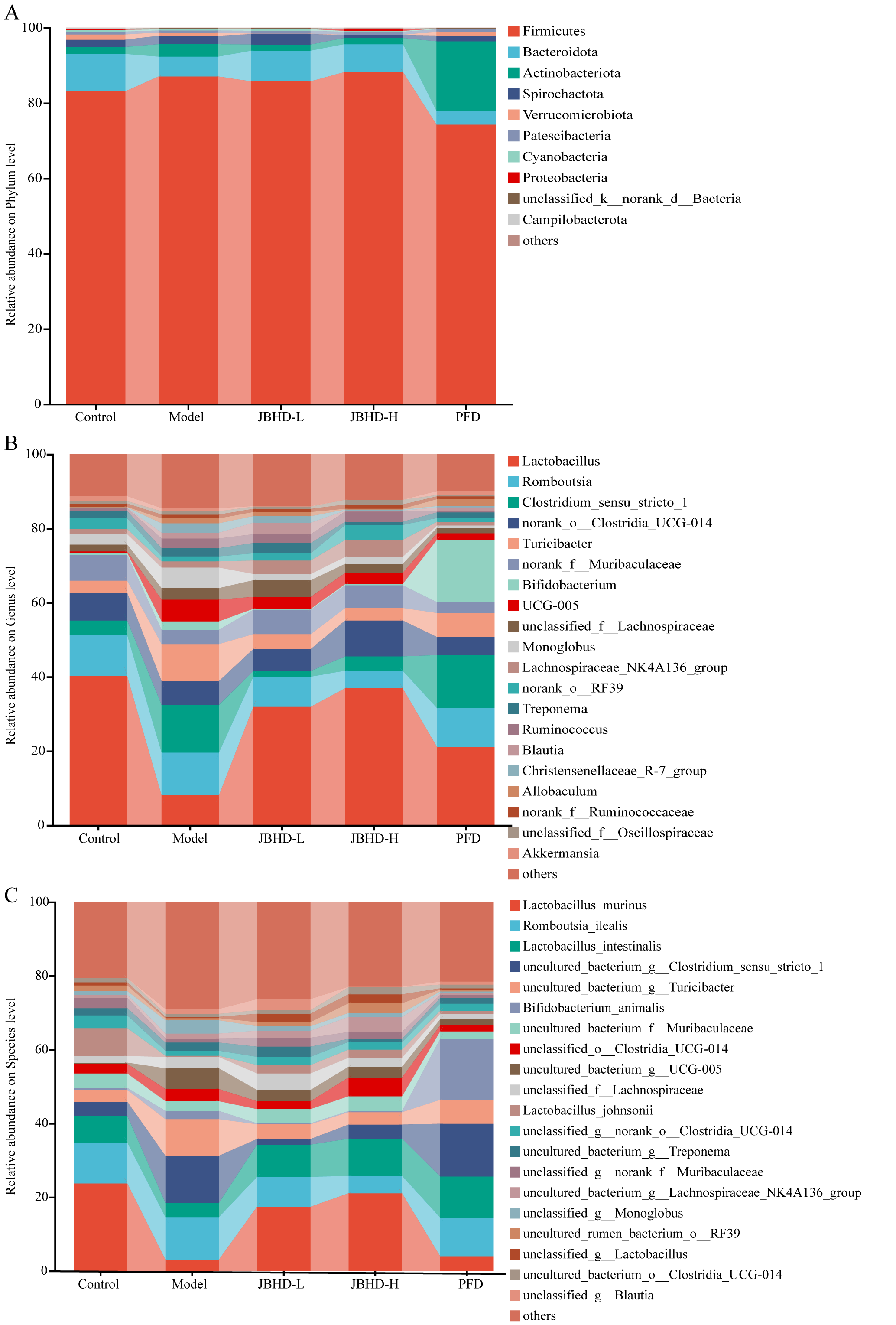


Supplementary Figure 3 Microbial community bar plot at (A) phylum level, (B) genus level, (C) species level. JBHD-L and JBHD-H, Jiawei Buyang Huanwu Decoction at low (10 g/kg) and high (20 g/kg) doses, respectively; PFD, Pirfenidone capsules.


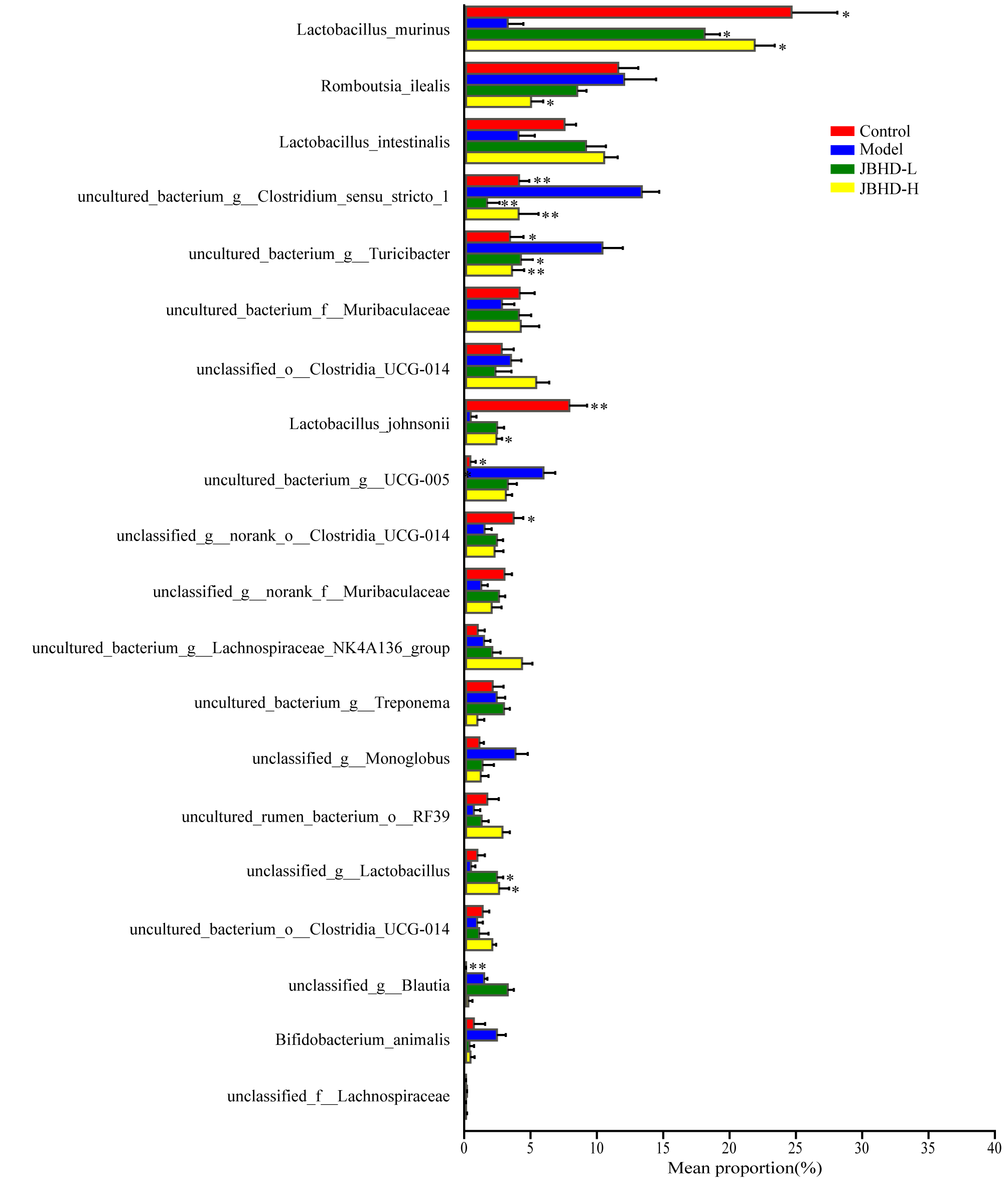


Supplementary Figure 4 Species-level differential abundance analysis among groups. **p* < 0.05 and ***p* < 0.01 *vs.* Model group. Data were shown as mean ± SD. JBHD-L and JBHD-H, Jiawei Buyang Huanwu Decoction at low (10 g/kg) and high (20 g/kg) doses, respectively.


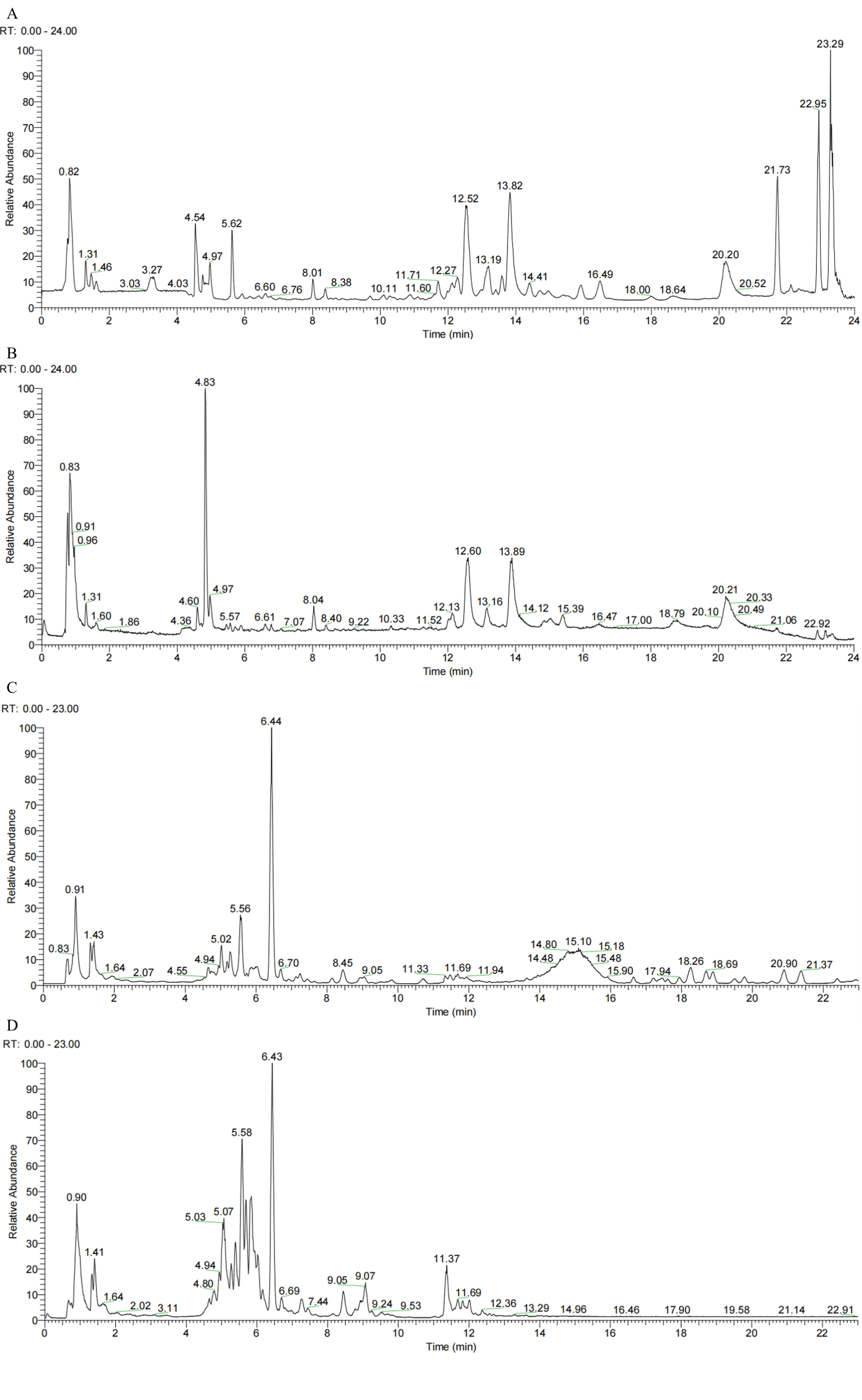


Supplementary Figure 5 The total ion chromatogram of the QC samples in serum samples (A) positive mode, (B) negative mode; urine samples (C) positive mode, (D) negative mode.


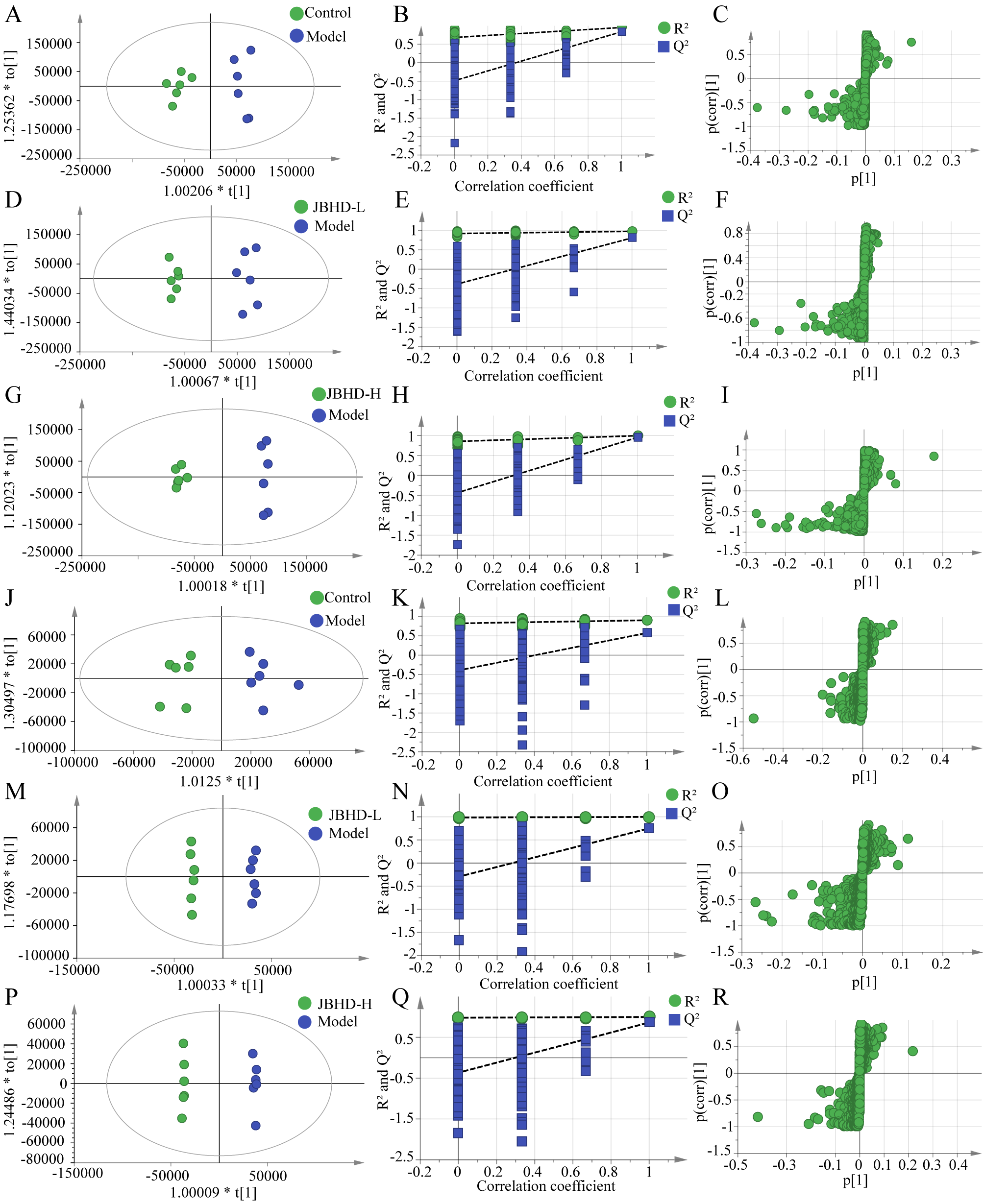


Supplementary Figure 6 OPLS-DA analysis of serum samples. OPLS-DA score plots, permutation tests and S-plots of Model vs. Control in positive (A, B, C) and negative (J, K, L) ion mode. OPLS-DA score plots, permutation tests and S-plots of JBHD-L vs. Model in positive (D, E, F) and negative (M, N, O) ion mode. OPLS-DA score plots, permutation tests and S-plots of JBHD-H vs. Model in positive (G, H, I) and negative (P, Q, R) ion mode.JBHD-L and JBHD-H, Jiawei Buyang Huanwu Decoction at low (10 g/kg) and high (20 g/kg) doses, respectively.


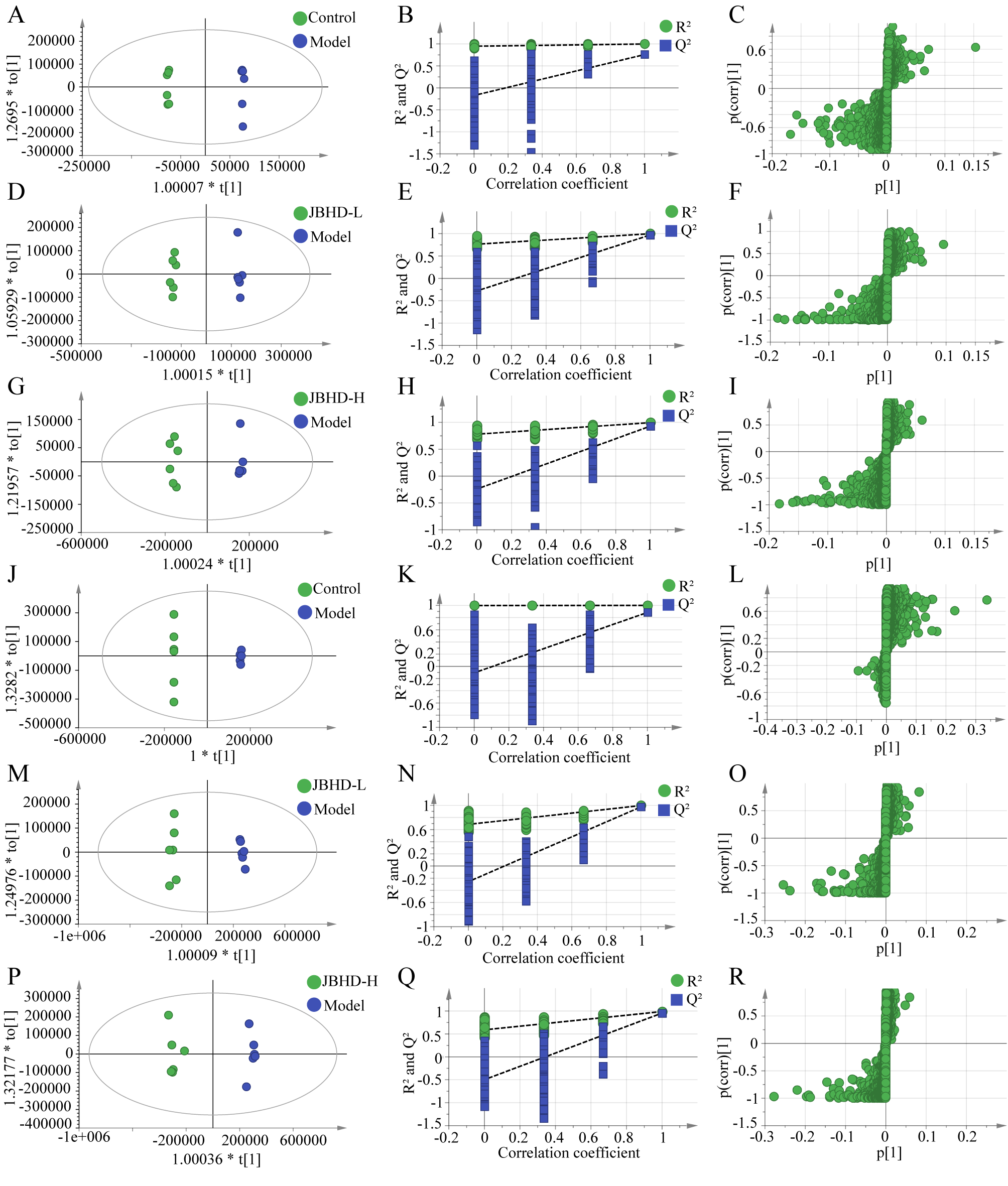


Supplementary Figure 7 OPLS-DA analysis of urine samples. OPLS-DA score plots, permutation tests and S-plots of Model *vs.* Control in positive (A, B, C) and negative (J, K, L) ion mode. OPLS-DA score plots, permutation tests and S-plots of JBHD-L *vs.* Model in positive (D, E, F) and negative (M, N, O) ion mode. OPLS-DA score plots, permutation tests and S-plots of JBHD-H *vs.* Model in positive (G, H, I) and negative (P, Q, R) ion mode. JBHD-L and JBHD-H, Jiawei Buyang Huanwu Decoction at low (10 g/kg) and high (20 g/kg) doses, respectively.


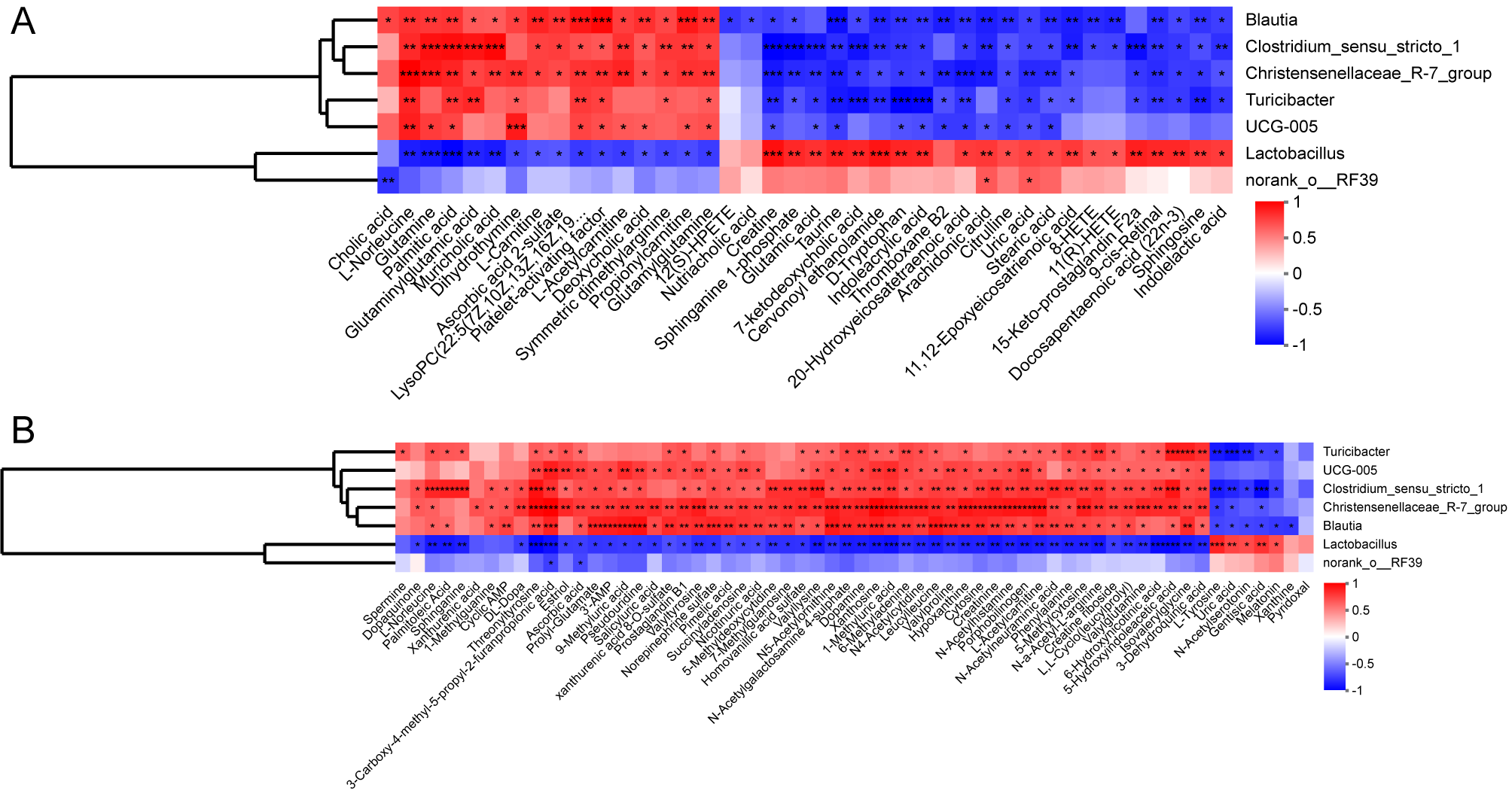


Supplementary Figure 8 The Spearman correlation analysis between the DAMFs and DEMs. Red and blue indicate positive and negative correlations, respectively. **p* < 0.05, ***p* < 0.01, ****p* < 0.001. DAFMs, differentially altered fecal microbes; DEMs, differentially expressed metabolites.
